# Exploring Neuronal Exosome miRNAs as Biomarkers of Neuroinflammation and Neuroplasticity in Amateur Boxers After Repetitive Head Trauma

**DOI:** 10.1007/s12035-026-05799-8

**Published:** 2026-03-15

**Authors:** Marica Pagliarini, Valentina Selleri, Luana Forleo, Alice Gualerzi, Caterina Ciacci, Roberta Saltarelli, Noemi Pappagallo, Andrea Minelli, Marcello Pinti, Marzia Bedoni, Gustavo Savino, Roberta D’Alisera, Maria Cristina Albertini, Milena Nasi, Patrizia Ambrogini

**Affiliations:** 1 Department of Biomolecular Sciences, University of Urbino Carlo Bo, 61029 Urbino, Italy; 2 Department of Neurology, Ulm University, 89081 Ulm, Germany; 3 Department of Life Sciences, University of Modena and Reggio Emilia, 41125 Modena, Italy; 4 IRCCS Fondazione Don Carlo Gnocchi, 20148 Milan, Italy; 5 Department of Public Healthcare, Sports Medicine Service, Azienda USL of Modena, 41121 Modena, Italy; 6 Department of Surgical, Medical, Dental and Morphological Sciences, University of Modena and Reggio Emilia, 41124 Modena, Italy

**Keywords:** Sports-related brain injury, MicroRNAs, Neuronal exosomes, Biomarkers, Neuroinflammation, Neural plasticity

## Abstract

Repetitive mild traumatic brain injuries, common in contact sports like boxing, are recognized risk factors for long-term neurodegenerative conditions. There remains a clinical need for reliable, non-invasive biomarkers capable of detecting early brain alterations induced by repeated head trauma. This study evaluated the potential of neuron-derived exosomal microRNAs (miRNAs) as indicators of neuroinflammatory and neuroplastic changes in amateur boxers exposed to recurrent mild head impacts. Head impacts were measured across three weekly sparring sessions in ten male athletes. Neuron-derived exosomes were isolated from plasma samples collected both before (T0) and after (T2) the sparring period. The expression of eight miRNAs implicated in neuroplasticity and inflammation (miR-34a, miR-9, miR-124a, miR-223, miR-132, miR-126, miR-146a, and miR-146b) was analyzed using RT-qPCR. Sparring elicited a marked decrease in miR-126 levels and a significant increase in miR-34a, miR-132, miR-223, miR-146a, and miR-146b, suggesting a coordinated molecular response involving neuroinflammatory signaling, vascular dysregulation, and altered neuroplastic processes. Notably, the number of recorded head impacts correlated positively with miR-146a expression, supporting its potential role as a sensitive marker of neuronal stress. Bioinformatic analysis of miRNA target genes revealed enrichment in pathways associated with neurogenesis, axonal repair, synaptic remodeling, and brain-region–specific expression patterns characteristic of neural stem cells. Together, these findings support the potential use of neuron-derived exosomal miRNAs as peripheral biomarkers for early detection of sports-related brain injury, offering mechanistic insight into subclinical neural adaptations induced by repeated head trauma. Their implementation may contribute to minimally invasive monitoring strategies for athletes at risk.

**Supplementary Information:**

The online version contains supplementary material available at 10.1007/s12035-026-05799-8.

## Introduction

Contact sports such as boxing, rugby, football, and martial arts expose athletes to repeated head impacts, thereby increasing the risk of concussions—commonly classified as mild traumatic brain injuries (mTBIs) [1]. These injuries result from linear and/or rotational forces acting on the brain, leading to axonal stretching and microstructural damage. Among traumatic brain injuries, mTBI represents the most prevalent form in athletic settings [2]. Although the majority of affected individuals recover within a few weeks, approximately 10–15% develop post-concussive syndrome (PCS), characterized by persistent cognitive, emotional, and somatic symptoms [3].

There is growing concern over the long-term consequences of repetitive mTBI, which has been associated with an increased risk for neurodegenerative diseases such as Alzheimer’s disease, Parkinson’s disease, and chronic traumatic encephalopathy [4].

Mechanistically, mTBI involves non-penetrating trauma that compromises the blood–brain barrier (BBB) integrity and induces diffuse vascular and tissue damage [5]. These events initiate a cascade of cellular and molecular responses that include oxidative stress, metabolic disruption, and widespread neuroinflammation [6].

Neuroinflammation is now recognized as a central driver of post-traumatic pathology. The initial mechanical insult activates resident glial cells—particularly microglia and astrocytes—leading to the release of pro-inflammatory cytokines, chemokines, and danger signals [7]. While acute inflammation may serve protective roles, its persistence can become maladaptive, exacerbating neuronal vulnerability and interfering with brain repair mechanisms [8]. Importantly, chronic or dysregulated neuroinflammation has been shown to impair adult hippocampal neurogenesis (AHN) [9]—a key neuroplasticity process involving the generation and functional integration of new neurons from neural stem/progenitor cells (NSPCs), primarily within the dentate gyrus of the hippocampus.

AHN is critical for memory formation, emotional regulation, and cognitive flexibility, functions that are frequently impaired following mTBI. Experimental models have shown that inflammatory mediators can alter NSPC proliferation, differentiation, and survival, potentially disrupting functional recovery after brain injury [10]. Understanding the interaction between neuroinflammation and neuroplasticity processes, such as neurogenesis, is crucial for identifying early biomarkers of cognitive risk and resilience in patients with mild traumatic brain injury.

In addition to local neuroinflammatory processes, systemic inflammation also contributes to the long-term effects of brain injury. In a previous study on the same cohort of amateur boxers [11], we found that circulating mitochondrial DNA (mtDNA) levels increased after each match and over the weeks, in patterns similar to neurofilament light [12]. MtDNA, as a damage-associated molecular pattern [13], increases with age and has been linked to neurodegenerative risk [14].

In this context, neuron-derived exosomes (NDEs)—small extracellular vesicles released by neurons—offer a promising non-invasive tool for monitoring brain-specific changes [15]. NDEs can cross the BBB and carry a molecular cargo (proteins, lipids, RNAs) that reflects the physiological state of their parent cells [16]. Of particular interest are microRNAs (miRNAs), short non-coding RNAs involved in post-transcriptional gene regulation. Enriched in exosomes, miRNAs have been implicated in both neuroplasticity processes [17] and neuroinflammatory signaling [18].

Several miRNAs are emerging as key regulators at the intersection of inflammation and neuroplasticity. For instance, miR-34a is upregulated in response to oxidative stress and brain injury promoting neuronal apoptosis and impairing NSPC proliferation [19]; miR-126 contributes to BBB integrity, exerts anti-inflammatory effects in TBI models [20] and its upregulation has been associated with neuronal recovery after injury [21]; miR-146a and miR-146b, known NF-κB targets, are induced in glial cells and modulate the innate immune response during CNS inflammation [21], while also playing a role in the regulation of adult neurogenesis [22]; and miR-223 expressed in both microglia and neurons is involved in immune regulation [23] and neural differentiation [24].

We hypothesize that repeated mild head trauma in boxers induces early alterations in the expression patterns of trauma-responsive neuronal miRNAs involved in neuroinflammation and neuroplasticity, including neurogenesis, which may be reflected in the molecular cargo of peripheral NDEs. Specifically, we expect upregulation of inflammation-associated miRNAs, indicative of immune stress, along with modulation of neurogenesis-related miRNAs, reflecting either an attempt to engage compensatory plasticity or an impairment of neurogenic potential. These exosomal miRNA signatures may represent early peripheral indicators of functional changes in the brain and provide insights into mechanisms underlying post-concussive symptoms. Ultimately, identifying reliable biomarkers of brain function and dysfunction could enhance diagnosis, prognosis, and treatment strategies, including personalized rehabilitation strategies, in sports-related brain injury.

## Material and Methods

### Subject Enrollment

Ten male amateur boxers, aged 18–39 years, were enrolled to participate in one sparring match per week for three consecutive weeks. Each match consisted of three rounds lasting 5 min each. Before the first match (T0) and within 2/3 h after the third match (T2), we collected venous blood samples for the subsequent analyses. A “controlled” boxing session (sparring) refers to a supervised training bout conducted under standardized conditions, in which athletes are matched by weight class and experience level and follow a predefined duration.

The subjects enrolled in the study were selected from those who had a period of rest before the T0 blood samples were collected, so we considered the first measurement at rest. The study was conducted at the beginning of the competitive season, during a period without official matches, when training primarily consisted of individual, no-contact training sessions. This timing minimized prior exposure to recent head impacts and reduced the potential confounding effects of cumulative head injuries before study enrollment. 

During the sessions, the number of hits each participant received to the head was recorded, as detailed in Table 1. Participant recruitment was conducted at the Sports Medicine Department of the AUSL of Modena. All enrolled athletes were certified as fit for sports activity by the Sports Medicine Service. The only exclusion criterion was the presence of inflammatory conditions or injuries occurring immediately before or during the study.

**Table 1:** Number of head impacts sustained by boxers in each match

Participants	1 st match	2nd match	3rd match
	nr of head blows	nr of head blows	nr of head blows
1	43	58	83
2	7	28	42
3	91	124	122
4	96	92	59
5	3	33	23
6	2	88	66
7	49	35	37
8	10	58	55
9	18	17	45
10	146	167	130

The study was conducted in accordance with the ethical recommendations of the Declaration of Helsinki and was approved by the Ethics Committee of the Emilia Nord Area (protocol number 1104/2021/SPER/AUSLMO — 0007077/22). The privacy rights of all participants were respected, and written informed consent was obtained from all individuals prior to participation.

### Two-Step Isolation of Neuron-Derived Exosomes

Neuron-derived exosomes were isolated using a validated L1CAM (CD171)-based immunoaffinity capture strategy. L1CAM/CD171 is a neuronal cell adhesion molecule, and its use enables the selective enrichment of NDEs while minimizing contamination from vesicles originating from non-neuronal cell populations [25, 26].

Venous blood samples were collected before the start of the sparring sessions (T0) and within 2/3 h after the third match (T2) and processed within 12 h to isolate plasma; plasma was aliquoted and then stored at − 80 °C.

Neuron-derived exosomes were isolated from 0.75 mL of frozen human plasma containing EDTA according to previous protocols [27, 28]. Briefly, samples were defibrinated using thrombin (System Biosciences, Inc., Mountain View, CA, USA) for 30 min at room temperature. The defibrinated samples were then treated with ExoQuick exosome precipitation solution (System Biosciences, Inc., Mountain View, CA). The ExoQuick-containing suspensions were incubated at 4 °C for 60 min to promote precipitation of total exosomes prior to centrifugation.

To enrich for exosomes containing the L1CAM, the suspensions were incubated overnight with 4 µg of biotinylated anti-human CD171 mouse antibody (CD171, clone 5G3, eBioscience, San Diego, CA, USA).

Ultralink streptavidin-agarose resin (Thermo Scientific, Rockford, IL, USA) in 3% BSA was then added, and incubation continued for 30 min at 4 °C with continuous mixing. Following centrifugation and removal of the supernatant, each pellet was resuspended in IgG elution buffer (Pierce™ IgG Elution Buffer, Thermo Fisher Scientific), mixed, centrifuged to separate L1CAM + exosomes from the bead–antibody complex, and neutralized with Tris–HCl at pH 8. The final suspensions containing neuronal exosomes were stored at − 80 °C.

### Nanoparticle Tracking Analysis

The NanoSight ns300 (Malvern Pananalytical) was used to assess exosome size distribution and concentration. Briefly, exosomes were diluted in pure PBS to obtain a 1 mL solution with a concentration of approximately 10^8^ particles per mL. The diluted sample was injected into the sample chamber via the injection tube, taking care to avoid introducing air bubbles. The sample was visualized under the camera, ensuring that laser illumination was evenly distributed across the field. Repeated measurements were taken, and each recording lasted 60 s.

### Immunoblotting of Neuronal Exosomes

Protein quantification of isolated exosomes was performed using the Pierce BCA Protein Assay Kit (Thermo Fisher Scientific), according to the manufacturer’s instructions. Subsequently, an equivalent amount of neuronal exosomes (17.6 µg of total protein for L1CAM and 5 µg for CD63), resuspended in a solution containing β-mercaptoethanol (βME) at 1:40, Laemmli buffer at 1:4 [29], and RIPA buffer [30], was denatured by sonication (10 pulses at 600 W), followed by incubation at 95 °C for 10 min.

Samples were then resolved by electrophoresis on 12% Tris–glycine precast gels (12% Mini-PROTEAN® TGX™ Precast Protein Gels). Membranes were blocked with 5% BSA in Tris-buffered saline (TBS) and incubated overnight with primary antibodies diluted in 5% BSA in TTBS (TBS and 0.5% Tween-20) at the following concentrations: 0.5 µg/mL for CD171 (Invitrogen Catalog #13–1719-82) or 2 µg/mL for CD63 (Invitrogen Catalog #10628D), under continuous agitation.

Blots were subsequently washed with 1X TBS, and then incubated in 5% BSA containing 0.5% Tween-20 and a horseradish peroxidase-conjugated goat anti-mouse secondary antibody. Unbound antibodies were removed by washing with TTBS buffer, and signal detection was carried out using the Clarity Western ECL Substrate kit and imaged with the Bio-Rad ChemiDoc Imager (Bio-Rad Laboratories, Hercules, CA, USA).

### Real-Time PCR (RT-qPCR)

Total RNA was isolated using the Total RNA Isolation Kit (Norgen Biotek, Thorold, ON, Canada) according to the manufacturer’s instructions. RNA concentration and purity were assessed spectrophotometrically using the NanoDrop ND 2000 (Thermo Scientific, San Jose, CA, USA). Isolated RNA was stored at − 80 °C until analysis.

For quantification, human miRNAs (miR-126, miR-132, miR-223, miR-34a, miR-124a, miR-146a, and miR-146b) were analyzed by RT-qPCR using the TaqMan MicroRNA Assay (Applied Biosystems, Foster City, CA, USA), following the manufacturer’s protocol. RT-qPCR data were normalized using the exogenous spike-in control cel-miR-39. The relative expression was calculated using the delta Ct method (ΔCt).

### Functional Enrichment Analysis

A comprehensive in silico functional enrichment analysis was performed using the Enrichr platform [31] to explore potential functional associations of the differentially expressed microRNAs identified in NDEs and their predicted downstream target genes. Target genes were obtained from the miRDB database [32], and only high-confidence targets with a prediction score ≥ 70 were included (S1 Table). Enrichment analyses were conducted across multiple annotation resources, including Gene Ontology (Biological Process and Cellular Component), KEGG pathways, the Jensen Diseases database, and the ARCHS4 tissue expression database to assess brain-related enrichment. Statistical significance was defined as *p* < 0.05. The pathways highlighted in the main figures and discussion were selected based on statistical significance and relevance to neuronal and brain-related biological processes, consistent with the neuronal origin of the analyzed exosomes. The complete enrichment results, including all significantly enriched terms, are provided in the Supplementary Material (S2 Table) to ensure transparency and reproducibility.

### Statistical Analysis

The data are presented as mean ± SEM or SD as specified in the figure legends. Spearman’s rank correlation was used to assess the association between the number of head impacts and microRNA expression levels. Outliers were identified using the ROUT method (*Q* = 1%), based on the false discovery rate (FDR). Data normality was assessed using the Shapiro–Wilk test. The Wilcoxon paired *t*-test was performed for comparisons between the T0 and T2 groups. Statistical analyses were carried out using GraphPad Prism version 9.5.1 (GraphPad Software, Boston, MA, USA). The significance threshold was set at *p* = 0.05.

## Results

### Characterization of Neuron-Derived Exosomes Isolated from Peripheral Plasma

**Fig. 1:**
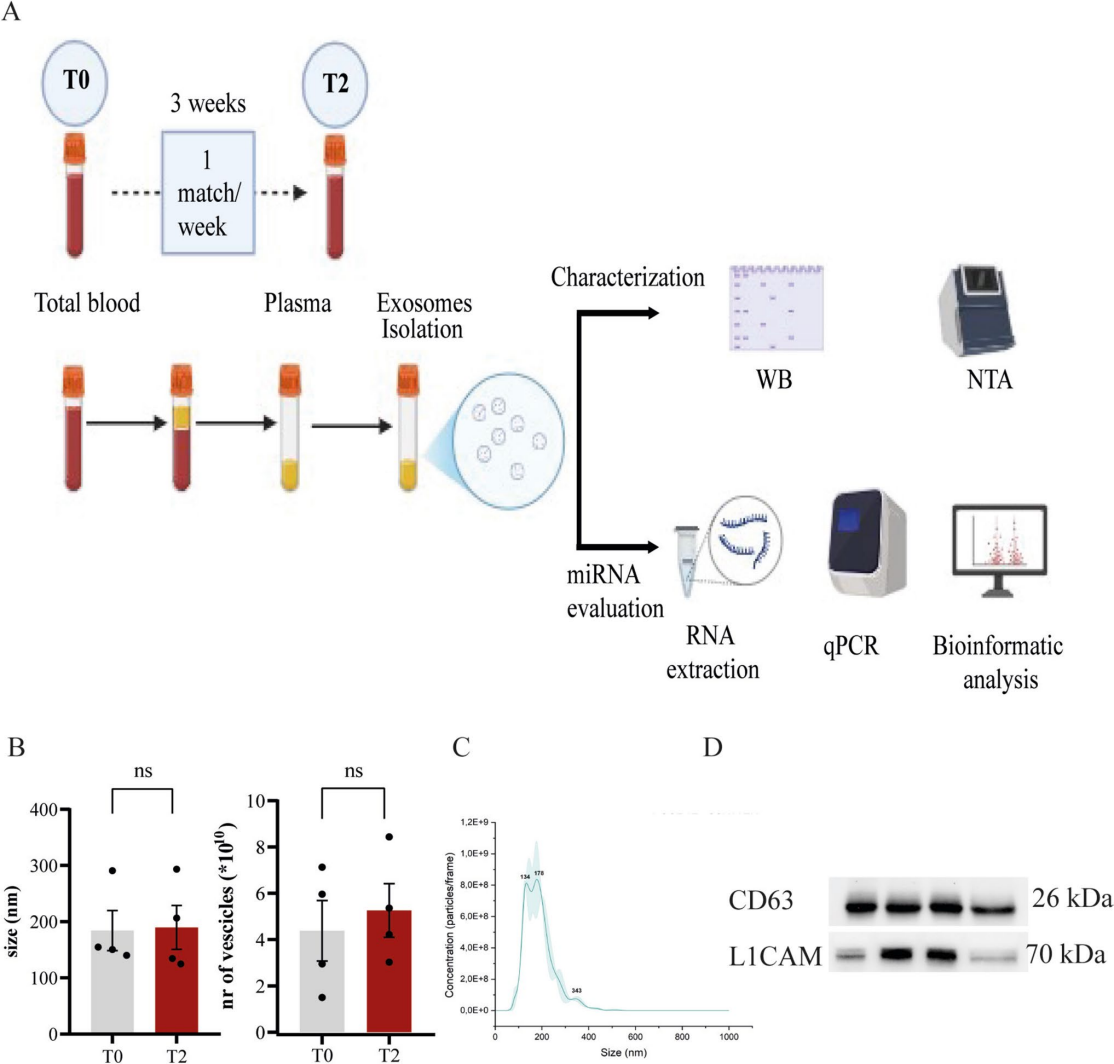
Experimental protocol and characterization of neuron-derived exosomes isolated from plasma. **A** Schematic representation of the experimental design: boxers participated in three sparring sessions, each spaced one week apart, over a total period of 3 weeks. Plasma samples were collected both before (T0) and after (T2) the sparring period. Subsequently, NDEs were isolated from plasma, and the expression of the indicated miRNAs was evaluated. **B** Quantification and dimension of circulating neuron-derived exosomes across samples revealed comparable concentrations, supporting the reproducibility and reliability of the isolation protocol. Data are expressed as mean ± SD. Number of samples: T0 (4), T2 (4). Mann–Whitney *U* test was performed. **C** Representative image of nanoparticle tracking analysis (NTA) of isolated extracellular vesicles showing a size distribution between 50 and 150 nm, consistent with canonical exosomal profiles. **D** Molecular characterization of vesicles: Western blot analysis confirms the presence of CD63, a classical exosomal marker, and enrichment for L1CAM, a neuron-specific exosomal marker. SDS-PAGE gels were cropped to show the precise localization of the band according to the molecular weight

The experimental protocol is outlined in Fig. 1A. Briefly, participants engaged in three controlled boxing sessions, each separated by a 1-week interval, over a total period of 3 weeks. This time window was strategically selected to align with the temporal dynamics of adult hippocampal neurogenesis, a process that spans approximately 4 weeks from the proliferation of radial glia-like neural precursors to the synaptic integration of newly generated neurons within the hippocampal circuitry [33].

To quantitatively assess the concentration and size distribution of NDEs in plasma, we employed nanoparticle tracking analysis, a method widely used for high-resolution characterization of extracellular vesicles. The isolated vesicles exhibited a size profile predominantly between 50 and 150 nm, consistent with the canonical size range of exosomes [34]. Notably, particle counts were consistent across individual samples and experimental time points, supporting the reproducibility and technical robustness of the isolation protocol (Fig. 1B, C).

Immunophenotyping of the vesicles revealed expression of CD63, a tetraspanin and canonical exosomal marker [35], confirming the exosomal nature of the isolated particles. Moreover, the vesicles were enriched for L1CAM, a neuronal cell adhesion molecule selectively expressed on NDEs [36], further validating the neuronal origin of the isolated fraction (Fig. 1D). These findings are in line with prior reports characterizing exosome populations derived from neuronal sources [27].

Collectively, these results indicate that the adopted isolation and characterization pipeline enables the reliable recovery of neuron-derived extracellular vesicles from peripheral blood. The morphological and molecular features of the isolated vesicles are consistent with neuronally derived exosomes.

### Analysis of miRNA Content in Circulating Neuron-Derived Exosomes

We focused on a panel of eight miRNAs (miR-34a, miR-9, miR-124a, miR-223, miR-132, miR-126, miR-146a, and miR-146b), selected based on their established roles in neural cell function and stress responses. Expression levels were quantified in NDEs isolated from peripheral plasma, collected before (T0) and after (T2) the 3-week sparring protocol.

**Fig. 2:**
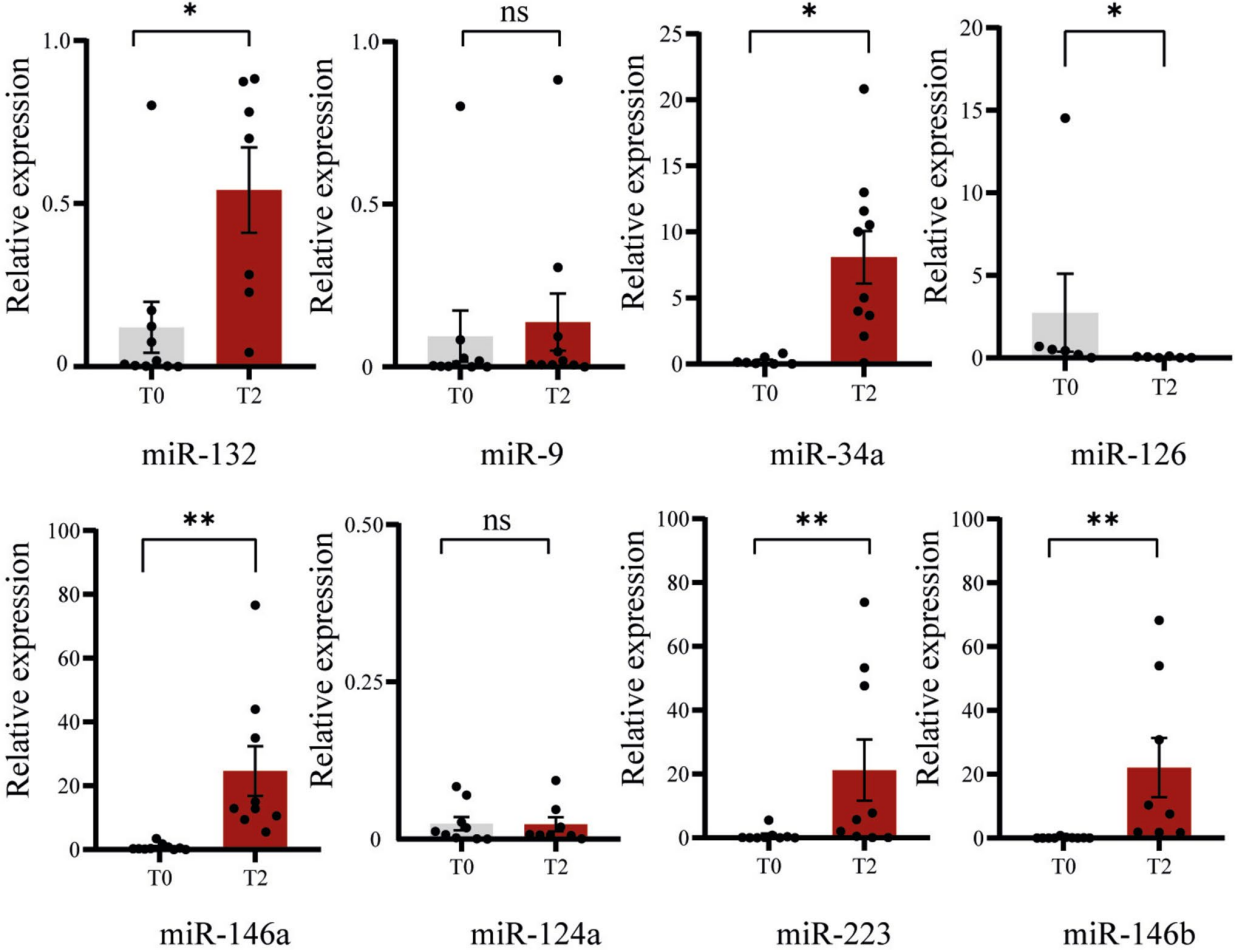
Quantitative RT-PCR analysis of neurogenesis-related miRNAs in neuron-derived exosomes before and after repeated sparring. Expression levels of eight miRNAs (miR-9, miR-132, miR-34a, miR-146a, miR-126, miR-124a, miR-223, and miR-146b) were quantified in neuron-derived exosomes isolated from plasma samples collected at baseline (T0) and after three weekly sparring sessions (T2). Data were normalized to the exogenous spike-in cel-miR-39 and expressed as mean ± SEM. Sample size: *n* = 10 per group. Statistical comparisons between time points were performed using the Wilcoxon signed-rank test. *p < 0.05; **p < 0.01

Our results revealed significant changes in miRNA expression between T0 and T2. Specifically, miR-34a, miR-223, miR-132, miR-146a, and miR-146b were significantly upregulated at T2 compared to baseline. In contrast, the expression of miR-126—which supports BBB integrity, limits inflammation in TBI, and aids neuronal recovery—was significantly downregulated at T2. No significant differences were observed for the proliferation-related miRNA miR-9 [37] and miR-124a [38] between time points (Fig. 2).

Together, these findings suggest that specific miRNA signatures in circulating neuron-derived exosomes may serve as sensitive indicators of early neurobiological signs to repetitive head impacts in athletes.

**Fig. 3:**
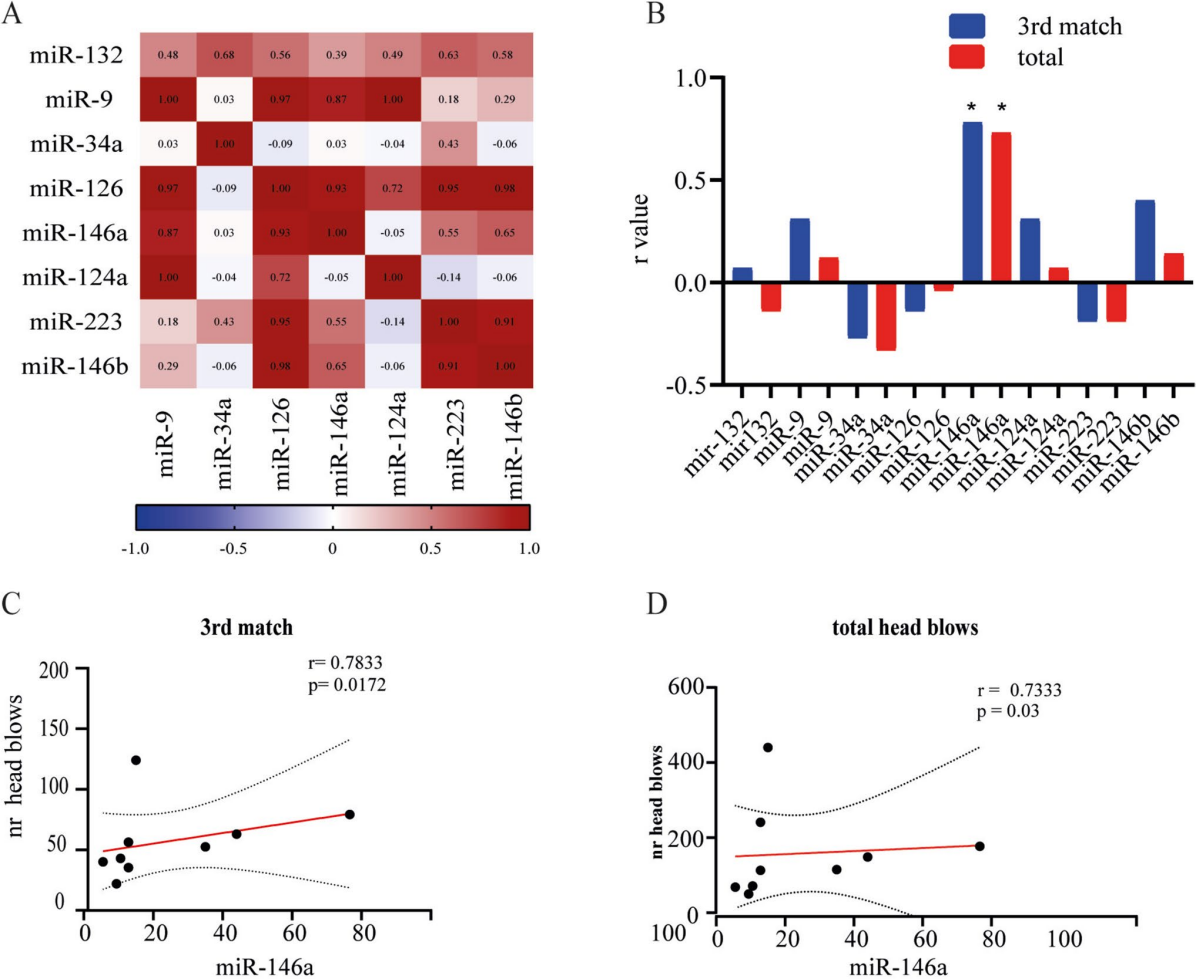
Correlation analysis of miRNA expression levels in neuron-derived exosomes. **A** Heatmap of pairwise Pearson correlation coefficients among the eight analyzed miRNAs in circulating neuron-derived exosomes. Several miRNAs showed significant positive correlations, suggesting coordinated regulation of neuroinflammatory and neurogenic processes. **B** Overall correlation of microRNA analyzed and number of head blows. **C**–**D** Scatter plot showing a significant positive correlation between miR-146a expression levels and the number of head impacts after different matches. Spearman’s correlation was used

A correlation analysis of miRNA expression levels within our panel revealed significant positive correlations among several miRNAs, suggesting a functional interplay between neuroinflammatory and neurogenic processes, and supporting the utility of this panel for integrated profiling of these mechanisms (Fig. 3A). We extended this analysis to examine the relationship between all analyzed miRNAs and the number of head impacts sustained by the boxers (Fig. 3B). miR-146a expression levels showed a significant positive correlation with head impact counts (Fig. 3B–D). In contrast, none of the other analyzed miRNAs exhibited significant associations with head impact number (Fig. 3B), although several showed significant inter-correlations with miR-146a expression (Fig. 3A).

### Gene Ontology and Pathway Analysis of miRNA Targets Implicated in Neurogenesis

**Fig. 4:**
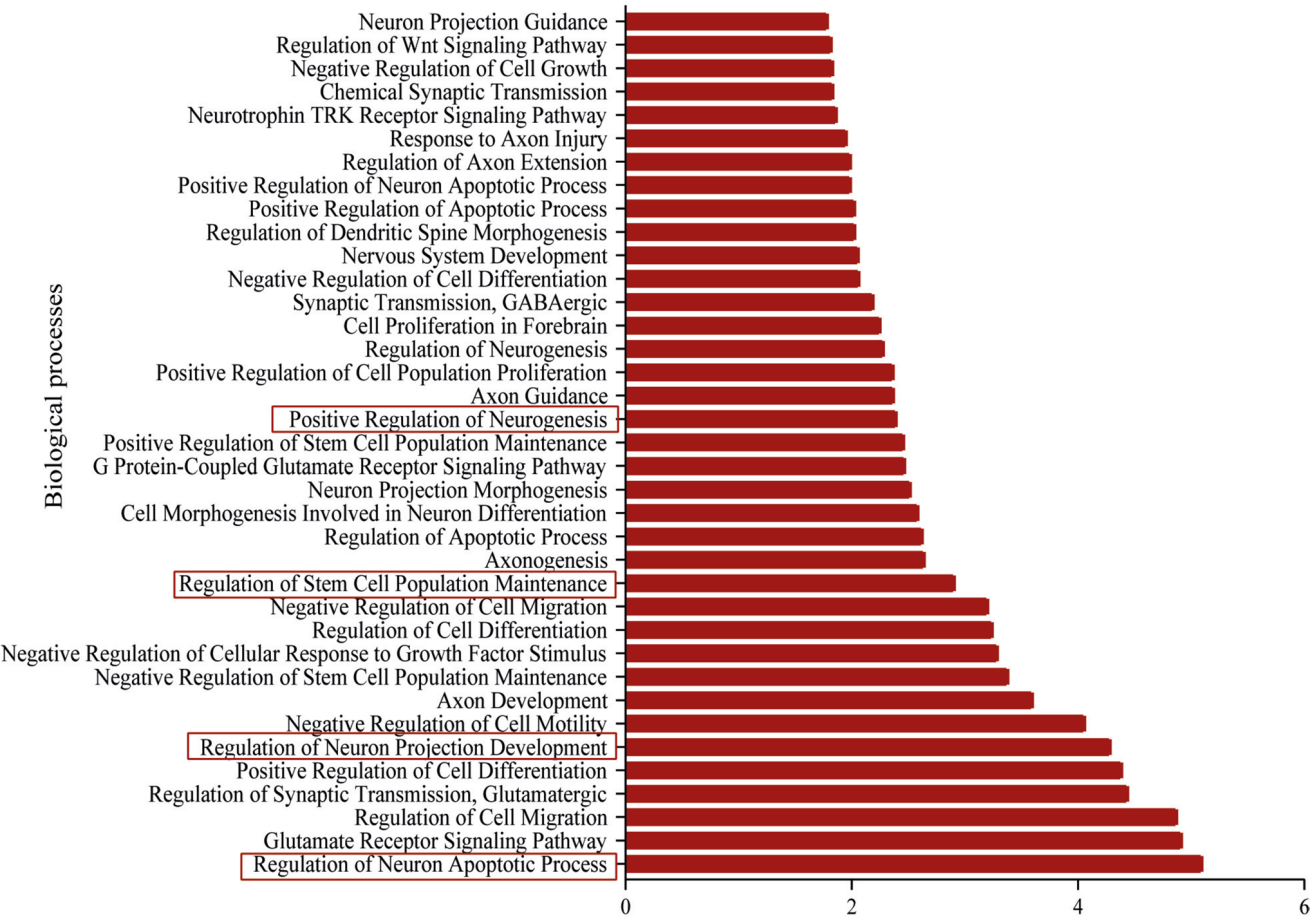
Functional enrichment analysis of predicted target genes of differentially expressed miRNAs in neuron-derived exosomes. Gene Ontology (GO) Biological Process analysis revealed significant enrichment in processes associated with neural repair and plasticity, including “positive regulation of neurogenesis,” “regulation of stem cell maintenance,” “neuronal projection development,” and pathways related to axonal injury and neuronal apoptosis

To further investigate the potential functional implications of the differentially expressed miRNAs identified in NDEs, we performed a comprehensive in silico enrichment analysis. Predicted miRNA target genes, using miRDB database [32], were subjected to Gene Ontology (GO) analysis using the EnrichR platform [31], allowing classification into biological processes (BP) and cellular components (CC). GO Biological Process enrichment (Fig. 4) revealed multiple categories highly relevant to neural development and repair mechanisms. Among the most significantly enriched terms were “positive regulation of neurogenesis,” “regulation of stem cell maintenance,” and “regulation of neuronal projection development,” supporting the hypothesis that these miRNAs are involved in orchestrating neuroplasticity-related processes, such as adult neurogenesis. Additional enriched terms included biological processes associated with the cellular response to axonal injury [39], neuronal apoptosis, and growth factor-mediated signaling—pathways commonly activated in the neuronal context of repetitive mild brain trauma [40].

**Fig. 5:**
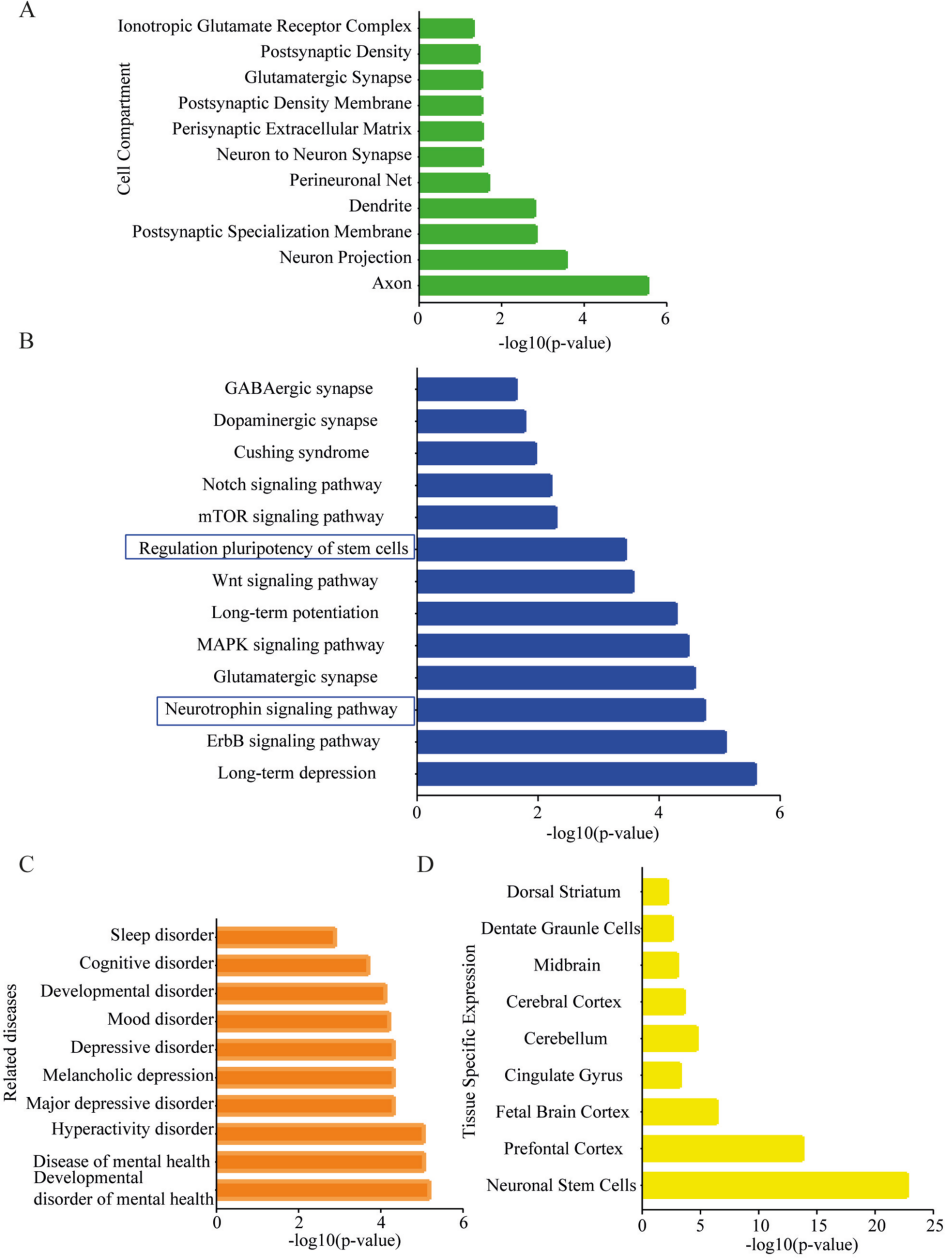
Functional enrichment analysis of predicted target genes of differentially expressed miRNAs in neuron-derived exosomes. **A** Subcellular regions critical for synaptic function, including the “postsynaptic density,” “dendrites,” and “axons,” indicating spatial relevance to synaptic plasticity. **B** KEGG pathway analysis revealed enrichment in canonical signaling cascades involved in neurogenesis and plasticity. GO Cellular Component enrichment analysis showed that the miRNA target genes were significantly associated with key pathways, most notably the Notch and Wnt signaling pathways. **C** Disease enrichment demonstrated that miRNA target genes were associated with central nervous system disorders, including neurodevelopmental syndromes, major depressive disorder, and cognitive dysfunction. **D** Brain-region enrichment analysis revealed a preferential expression of miRNA target genes in neuronal stem cells

Given the predominance of neurodevelopmental terms in the GO-BP results, we conducted a parallel enrichment analysis of Cellular Component categories (Fig. 5A) to contextualize the spatial and structural relevance of the predicted gene products. This analysis revealed strong enrichment in components related to synaptic structure and function, including “postsynaptic density,” “dendrite,” and “axon.”

To identify the broader signaling networks potentially regulated by these miRNAs, we performed KEGG pathway analysis (Fig. 5B). This revealed a prominent enrichment of canonical pathways involved in neuroplasticity, particularly the Notch and Wnt signaling pathways. Both are well-established regulators of neural stem cell fate decisions [41], dendritic arborization [42], and synapse formation [43] and have been implicated in repair processes following central nervous system injury [41].

To assess whether the enriched pathways and biological processes were relevant to neuropathological conditions, we employed the Jensen Disease database for disease enrichment analysis (Fig. 5C). The top ten disease associations were predominantly CNS-related and included neurodevelopmental disorders, cognitive impairment, and major depressive disorder—conditions commonly linked to impaired neurogenesis and frequently reported in individuals with neurodegenerative diseases.

Finally, to evaluate the anatomical specificity of the miRNA-targeted gene expression, we performed a brain-region enrichment analysis using ARCHS4 database (Fig. 5D). The majority of the predicted gene targets were found to be preferentially expressed in neuronal stem cells and the prefrontal cortex. However, the downstream enrichment analysis is exploratory in nature and leverages existing databases of predicted or validated targets. This approach enables the identification of biologically relevant pathways associated with the selected miRNAs and provides valuable biological context for hypothesis generation, while future functional studies will help further validate these findings.

## Discussion

In the present study, we demonstrate that repeated traumatic brain injuries sustained during boxing matches are associated with specific changes in circulating neuron-derived exosomal miRNAs, reflecting neuroinflammatory signaling, vascular compromise, and altered neuroplasticity. These findings support the possibility of using exosomal miRNAs as non-invasive biomarkers for monitoring subclinical brain injury in athletes engaged in contact sports, consistent with evidence reported in the current literature [44]. Although exosomal miRNAs have been investigated in TBI, our study expands their application to the sub‑concussive domain by focusing specifically on neuron‑derived exosomes and by identifying an integrated miRNA signature that reflects neuroinflammation, vascular alterations, and neuroplasticity. The significant association between head‑impact count and miR‑146a further suggests that NDE‑miRNAs may serve as quantitative, dose‑responsive biomarkers of mechanical stress to the brain. These findings highlight the potential clinical relevance of NDE‑miRNAs as non‑invasive indicators for monitoring athlete brain health, guiding return‑to‑play decisions, and identifying individuals who may benefit from early intervention or longitudinal surveillance.

Among the differentially expressed miRNAs identified in our study, miR-34a emerged as particularly relevant due to its established roles in key neural processes. Following competition, there was a consistent upregulation of miR-34a, a well-known regulator of apoptosis, neuronal differentiation, and synaptic remodeling [45]. Its sustained expression aligns with prior observations linking miR-34a to pro-apoptotic signaling cascades [46] and suggests that neurotrauma may impair neuronal survival and plasticity, interfering with mechanisms of repair and functional recovery. These alterations could underlie the cognitive and behavioral impairments frequently reported in athletes subjected to recurrent concussive and sub-concussive impacts. Our findings indicate that miR-34a may function not only as a biomarker of brain injury but also as a potential mediator of pathological neuroadaptation, potentially influencing long-term neurological outcomes in athletes exposed to repetitive head trauma.

Similarly, miR-223 was upregulated following bouts. This miRNA regulates the inflammatory pathway [47], acting as a negative regulator of microglial activation and pro-inflammatory signaling; specifically, it modulates key components of the NF-κB and NLRP3 inflammasome pathways in microglia, thereby limiting the production of inflammatory mediators such as IL-1β, IL-6, and TNF-α. In addition, miR-223 has been implicated in neuronal differentiation, with evidence showing that its overexpression can inhibit cytoskeletal dynamics and the migration of neural precursors [48]. Conversely, miR-223 has also been reported to exert a neuroprotective effect by regulating glutamate receptor subunits GluR2 and NR2B, thereby reducing NMDA receptor-mediated calcium influx, limiting excitotoxic neuronal injury, and preserving synaptic function [49]. Taken together, the observed increase in miR-223 may reflect a complex, dual role, simultaneously modulating neuroinflammatory responses while influencing mechanisms of neuronal plasticity and regeneration.

Within the subset of miRNAs exhibiting differential expression, miR-132 was notably upregulated. Previous studies have demonstrated that overexpression of miR-132 in the rodent brain facilitates endogenous tissue repair and mitigates clinical symptoms following traumatic brain injury [50]. Specifically, miR-132 overexpression has been shown to reduce lesion volume, enhance neurogenesis in the dentate gyrus, and facilitate neuroblast migration to the injury site. In addition to these effects, miR-132 is well known for its role in dendritic branching and synaptic remodeling [51], suggesting it may participate in a compensatory plastic response following injury [52]. This is further supported by evidence that physical exercise induces miR-132 expression, which in turn is associated with enhanced synaptic plasticity, improved cognitive function, and neuroprotection.

Altered expression of miR-146a and miR-146b—two key regulators of innate immunity, microglial activation, and cytokine production[53]—was also evident. Excessive miR-146a and miR-146b expression has previously been linked to reduced synaptic plasticity [54] and impaired neurogenesis [22]. miR-146b, expressed in neurons and involved in stem cell differentiation [55], also exhibited expression changes consistent with neuronal injury-induced dysregulation [56]. Notably, in our cohort, neuron-derived exosome miR-146a levels were significantly elevated following repeated head impacts during boxing matches, with expression positively correlating with the number of impacts sustained. miR-146a expression was measured after the third match, and correlations were performed both with the number of head impacts in the third match and the total number of hits sustained across the three matches. While the results were consistent between these approaches, we cannot exclude that the measured miRNA levels may be more influenced by the most recent (third) match, reflecting an acute response. Nevertheless, this indicates a dynamic, neuron-intrinsic regulation of miR-146a in response to mechanical trauma. The selective enrichment of miR-146a in neuronal exosomes further supports its potential as a biomarker of subclinical neuronal injury and impaired neurogenesis in athletes exposed to repetitive mTBI.

In parallel, we observed a downregulation of miR-126, a miRNA essential for regulating blood–brain barrier permeability [57]. In the context of cerebral injury, miR-126 has been shown to support neurogenesis, as its reduced expression following ischemic stroke correlates with a decrease in DCX⁺ neuroblasts and NeuN⁺/BrdU⁺ neurons in affected brain regions [58]. In addition to its neuroregenerative function, miR-126 also plays a key role in modulating neuroinflammation by preserving BBB integrity and limiting vascular damage. These dual functions are likely interdependent, as the maintenance of BBB homeostasis helps restrict peripheral immune cell infiltration and dampen inflammatory signaling within the central nervous system, thereby fostering an environment conducive to tissue repair. Consequently, the observed downregulation of miR-126 may contribute both to impaired neural regeneration and to the amplification of neuroinflammatory responses, ultimately hindering recovery after brain injury.

Intriguingly, miR-9 and miR-124a—two miRNAs essential for neural stem cell self-renewal and early differentiation [38, 59]—did not show significant downregulation. This pattern may reflect the pro-neurogenic effects of physical activity, which is known to promote proliferation within adult neurogenic niches [60] by modulating the balance between neurogenic and anti-neurogenic signals. Therefore, rather than indicating a depletion of neural stem cell pools, the observed changes suggest the occurrence in mild brain trauma of a dynamic rebalancing of cellular turnover, potentially favoring the removal of mature or damaged neurons through apoptosis, while simultaneously integrating newly generated neurons. This interpretation is supported by the concurrent upregulation of pro-apoptotic and inflammation-associated microRNAs, such as miR-34a and miR-223.

Taken together, these findings highlight the complex interplay between vascular impairment, neuroinflammatory signaling, and neuroplastic adaptation following repeated head trauma. Due to their specificity and accessibility, exosomal miRNAs hold strong potential as candidate biomarkers for monitoring injury progression and identifying athletes at risk for long-term neurological consequences. Indeed, it is worth noting that disease enrichment analysis revealed that the miRNA target genes are associated with central nervous system disorders, including neurodevelopmental syndromes, major depressive disorder, and cognitive dysfunction. Furthermore, brain-region enrichment analysis indicated a preferential expression of these genes in the prefrontal cortex, a region critically involved in higher-order cognitive functions, in emotion regulation, and in orchestrating complex behaviors such as goal-directed planning, inhibitory control, and adaptive decision-making. These capacities are profoundly disrupted in the pathological sequelae of mild traumatic brain injuries.

This study has several limitations that should be acknowledged. First, it included only male amateur boxers due to the limited availability of female participants in the local amateur categories. We acknowledge that this limits the generalizability of the findings to female athletes, and future studies should include female boxers to explore potential sex differences. Second, the sample size was relatively small, potentially restricting the generalizability of the findings. To confirm these results, future studies should aim to include larger cohorts of athletes. Moreover, a comprehensive understanding of the temporal regulation of miRNA expression following repeated head impacts will require longitudinal sampling designs incorporating multiple post-injury intervals. In addition, pairing circulating biomarker profiles with high-resolution imaging modalities—such as 7 Tesla MRI for hippocampal volume assessment [61] and hyperpolarized 13 C MRI to evaluate regional metabolic shifts [62]—could enhance mechanistic interpretation and improve diagnostic sensitivity. A further limitation of this study is the lack of detailed information regarding each athlete’s previous exposure to head impacts, including concussion history and cumulative sub-concussive trauma. These factors may influence baseline levels of neuroinflammatory and neuroplasticity-related biomarkers, including exosomal miRNAs, and therefore represent potential sources of inter-individual variability. Although the study design relied primarily on within-subject comparisons (T0 vs. T2), which reduces—but does not eliminate—the influence of pre-existing differences, the absence of comprehensive retrospective exposure data limits the interpretation of the variability between subjects. Future investigations would benefit from standardized assessments of lifetime head impact burden to better characterize how prior trauma shapes biomarker profiles and modulates the neurobiological response to new impacts. Finally, a limitation of the present study is the two-time-point design, which captures overall changes between measurements but does not allow detailed characterization of the temporal dynamics of the miRNA response. Additional intermediate and follow-up time points would be necessary to better define the kinetics and trajectory of miRNA expression following impact exposure. Future studies incorporating more comprehensive longitudinal sampling may help clarify the timing, peak response, and resolution patterns of these molecular signals.

Elucidating the temporal dynamics and functional relevance of these molecular changes through expanded longitudinal studies may pave the way for early detection strategies and targeted interventions aimed at mitigating long-term neurological consequences of repetitive head trauma. From a translational perspective, our findings suggest a dual potential application: (i) miR-146a emerges as a promising candidate for biological “dosimetry,” providing a sensitive index of neuronal stress related to head injury; (ii) the coordinated modulation of multiple neuron-derived exosomal miRNAs appears to reflect broader neuroinflammatory and neuroplastic processes, indicating that a multi-miRNA signature may ultimately offer greater specificity and predictive value for downstream functional and clinical outcomes than any single biomarker alone. Collectively, these results provide strong bioinformatic evidence that the miRNA signature identified in NDEs is functionally linked to neurogenic and neuroinflammatory processes activated by subclinical brain trauma and may offer valuable insight into early molecular events underlying maladaptive plasticity and long-term cognitive vulnerability in contact sport athletes.

## Conclusion

This study shows that neuron-derived exosomal miRNAs involved in neuroinflammation and neuroplasticity—particularly those linked to neurogenesis—are significantly dysregulated following repetitive mild head trauma in amateur boxers. These alterations, including the impact-related increase of miR-146a, suggest early neurobiological stress responses and a potential reduction in neurogenic capacity in vulnerable brain regions such as the hippocampus. Such mechanisms may contribute to the subtle cognitive and emotional changes reported over time in contact sport athletes. Overall, our findings highlight the promise of circulating exosomal miRNAs as minimally invasive biomarkers for detecting early, subclinical brain alterations induced by repeated head impacts.

## Supplementary Information

Below is the link to the electronic supplementary material.

ESM 1 (144 KB PDF)

ESM 2 (405 KB PDF)

ESM 3 (324 KB PDF)

## Data Availability

The data used to support the findings of this study are available from the corresponding author upon request.
