# Supplementary material for: Exploring Neuronal Exosome miRNAs as Biomarkers of Neuroinflammation and Neuroplasticity in Amateur Boxers After Repetitive Head Trauma
Source: Mol Neurobiol. 2026 Mar 15;63(1):501. doi: 10.1007/s12035-026-05799-8 (PMC12989019; doi:10.1007/s12035-026-05799-8)
Supplement: Supplementary file 1 — (144 KB PDF) [file 12035_2026_5799_MOESM1_ESM.pdf]

Article title:

Exploring Neuronal Exosome miRNAs as biomarkers of Neuroinflammation and Neuroplasticity in Amateur Boxers After Repetitive Head Trauma

Journal name

Molecular Neurobiology

Author names

Marica Pagliarini[#], Valentina Selleri[#], Luana Forleo, Alice Gualerzi, Caterina Ciacci, Roberta Saltarelli, Noemi Pappagallo, Andrea Minelli, Marcello Pinti, Marzia Bedoni, Gustavo Savino, Roberta D'Alisera, Maria Cristina Albertini, Milena Nasi, Patrizia Ambrogini*

# Marica Pagliarini and Valentina Selleri contributed equally to the study.

Affiliation and e-mail address of the corresponding author

*Department of Biomolecular Sciences, University of Urbino Carlo Bo, 61029 Urbino, Italy

patrizia.ambrogini@uniurb.it

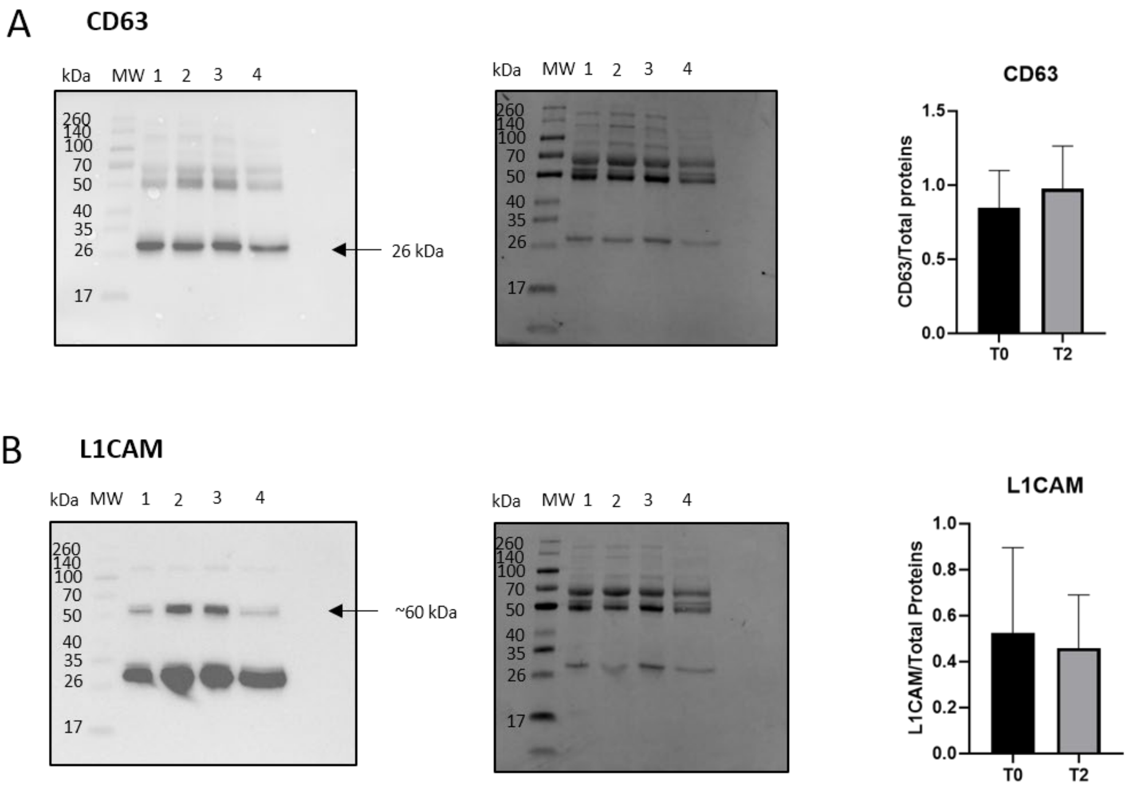

Figure 1S. Western blot analysis of neuron-derived exosomes isolated from plasma samples of amateur boxers

Representative original western blot images and corresponding Coomassie stained blots along with densitometric analysis for (A) CD63 and (B) L1CAM in exosomes isolated from plasma samples collected both before (T0) and after (T2) the sparring period. The total amount of proteins in each lane was used for the normalization of targets proteins. Data are represented as mean ± SD. The experiments were repeated independently with similar results. MW, molecular weight ladder peqGold pre-stained protein Marker VI (260-10kDa); lines1and 2:T0 samples; lines 3 and 4: T2 samples.
