# Supplementary material for: Exploring Neuronal Exosome miRNAs as Biomarkers of Neuroinflammation and Neuroplasticity in Amateur Boxers After Repetitive Head Trauma
Source: Mol Neurobiol. 2026 Mar 15;63(1):501. doi: 10.1007/s12035-026-05799-8 (PMC12989019; doi:10.1007/s12035-026-05799-8)
Supplement: Supplementary file 2 — (405 KB PDF) [file 12035_2026_5799_MOESM2_ESM.pdf]

miRRBD

	miR-34a	miR-223	miR-132	**mir-126**	mir-146a	mir-146b	Common list
1	MSR1	FBXW7	CDK19	HSPB8	TRAF6	TRAF6	MSR1
2	ADGRF4	SP3	TIMM9	PDE7B	IRAK1	IRAK1	ADGRF4
3	TNF	KPNA3	LEMD3	FBXO6	SEC23IP	SEC23IP	TNF
4	CAB39	RRAS2	SOX5	TRMT9B	NOVA1	NOVA1	CAB39
5	SCYL2	SLC4A4	CBLL1	TNFAIP8L3	PPP1R11	UPP2	SCYL2
6	PDCD6IP	CRIM1	MIA3	HOXB6	UPP2	WWC2	PDCD6IP
7	SHCBP1	ATP7A	RGS7BP	GNE	WWC2	PPP1R11	SHCBP1
8	KDM5B	PAX6	MIER1	NAT1	BCORL1	BCORL1	KDM5B
9	ARHGEF35	GALNT18	METTL25	CASP9	ZNF649	SORT1	ARHGEF35
10	CADM1	FOXO1	MAPK1	TRIM8	SORT1	ZNF649	CADM1
11	YAP1	SIAH1	GTF2H1	TMEM182	NUMB	NUMB	YAP1
12	G3BP2	F3	RASA1	MR1	USP32	USP32	G3BP2
13	TMEM176B	UBR5	MEX3C	ESRRG	PLSCR4	PLSCR4	TMEM176B
14	DEUP1	PTBP2	TUT4	S1PR3	CARD10	GDAP1L1	DEUP1
15	TET1	INPP5B	SGK3	A2ML1	SRSF6	SIAH2	TET1
16	KIDINS220	CBX5	CHD1	MDM4	APPL1	SRSF6	KIDINS220
17	GALNT1	HSP90B1	USP38	BRWD3	ZNF652	APPL1	GALNT1
18	WDR72	ARMCX1	DAZAP2	RFX4	SIAH2	ZNF652	WDR72
19	RMI1	FBXO8	VDAC2	TRPS1	GDAP1L1	CARD10	RMI1
20	MRC1	SLC35G2	MELK	ARL11	SLC10A3	MRS2	MRC1
21	MIEF1	RHOB	FEM1C	FAM168A	MRS2	EIF4G2	MIEF1
22	RUNX1	SEPT6	SSH2	CASK	ZNF354B	ZNF540	RUNX1
23	ZC3H15	ADCY7	BRWD1	DENND1B	C9orf72	SLC10A3	ZC3H15
24	SNX4	LMO2	ZBTB20	GRIK2	EIF4G2	C9orf72	SNX4
25	PARP16	KAT6A	CSDE1	TBCA	ZNF540	ZNF354B	PARP16
26	NSD1	RUBCNL	MYCBP2	HOXC8	GRIA3	GRIA3	NSD1
27	GORAB	ACSL3	HBEGF	INPP5D	LOC388813	DDHD1	GORAB
28	HSDL1	FEZ2	CCDC88A	CAMK2A	DDHD1	LOC388813	HSDL1
29	EIF3CL	ANKS1B	PTBP2	GABRA4	BRK1	TDRKH	EIF3CL
30	PALLD	HMGCS1	MTF2	CD84	TDRKH	BRK1	PALLD
31	ZNF407	NDNF	ZNF521	GGT6	ZBTB2	NEMP1	ZNF407
32	SCRN3	LSM8	SOD2	TSC22D4	CD80	CD80	SCRN3
33	SLC22A5	TENT5A	SETD5	MACC1	FBXW2	DCAF12	SLC22A5
34	EIF3C	HARBI1	SERP1	GABRB2	POFUT2	ZBTB2	EIF3C
35	XRN2	INPP4A	NFE2L2	RORA	NEMP1	CXXC4	XRN2
36	TACC2	SLC8A1	KLF7	MAP3K2	DCAF12	FBXW2	TACC2
37	CERS6	SLC7A8	OSBPL8	FGF7	CXXC4	POFUT2	CERS6
38	PTAR1	TMEM143	ZNF652	CNTN1	KCNJ16	ABL2	PTAR1
39	PRSS12	ZNF772	TJAP1	SETD9	USP6	KCNJ16	PRSS12
40	LRP2	RBPJ	ETNK1	CHST11	ABL2	USP6	LRP2
41	RAB3IP	SMIM15	PCDH10	PI15	HNRNPD	HNRNPD	RAB3IP
42	HDAC2	TWF1	ZNF516	VPS13A	MMP16	MMP16	HDAC2
43	MAP3K4	FKBP5	DAAM1	YIPF6	RHOBTB3	HIPK3	MAP3K4
44	APOBEC4	LAMB1	HHIP	PNRC1	CTAGE9	CTAGE4	APOBEC4
45	EHMT1	NFIB	SLC6A1	MYEF2	HIPK3	CTAGE9	EHMT1
46	SUMO1	INHBB	NOVA1	KCNB1	RARB	CTAGE8	SUMO1
47	RNF44	LACC1	CCN2	COMMD2	FOXR2	RHOBTB3	RNF44
48	ECHS1	PARP1	NMNAT2	SHISAL1	CTAGE8	RARB	ECHS1
49	RNPEP	PDS5B	SPPL3	FXN	CTAGE4	FOXR2	RNPEP
50	RFFL	CDK17	HNRNPH1	TUBGCP4	SRP72	SLC38A1	RFFL
51	SPX	KIAA1841	FBXL20	MMRN1	VASN	SRP72	SPX
52	ZNF480	RBSN	SKAP2	ZDHHC15	FBXO4	VASN	ZNF480
53	ADSS	SDC2	TLN2	PKHD1	SLC38A1	FBXO4	ADSS
54	TCAIM	RASA1	GMFB	KIRREL1	ZNF275	TMEM19	TCAIM
55	SH3GLB1	USP40	SAP30L	STEAP2	SLC12A6	GPM6B	SH3GLB1
56	PCDH19	SLC37A3	PPM1G	RABL3	TMEM19	C3orf38	PCDH19
57	SELENOI	OLFM1	SLC25A28	EBF1	ZNF90	C8orf88	SELENOI
58	NMT2	SNX6	ELMSAN1	ZIC5	NOS1	YWHAB	NMT2

59	CRIPAK	IGF1R	NVL	DBNDD2	ZNF506	SLC12A6	CRIPAK
60	DEPDC5	TMEM178B	RUFY3	C5orf47	GPM6B	ZNF90	DEPDC5
61	TMEM74	ARHGEF38	RPP14	ZBTB41	LRCH1	NOS1	TMEM74
62	ABHD10	SORBS1	DCC	SLC25A53	C3orf38	ZNF275	ABHD10
63	USP37	ZBTB42	L3MBTL3	TSHZ3	MAP3K8	ZNF506	USP37
64	PHF6	MAEL	FAM227A	TTF2	C8orf88	LRCH1	PHF6
65	ANTXR2	C18orf54	ASF1A	AMFR	YWHAB	MAP3K8	ANTXR2
66	DDX27	TSPAN7	PHF12	SRSF12	TCF20	MED20	DDX27
67	GPN1	NFIA	USP9X	TECPR2	CD96	YES1	GPN1
68	NTRK2	MYO5B	STX16	ACAN	MED20	TCF20	NTRK2
69	RNF135	PRDM1	RB1	SYT10	YES1	CD96	RNF135
70	UQCC1	NUP210	ACVR2B	ADH4	ARL10	ARL10	UQCC1
71	ZHX1	CYTIP	NACC2	VWC2	STRBP	STRBP	ZHX1
72	PAXBP1	SREK1	TMEM164	RINL	PTPRA	PTPRA	PAXBP1
73	HRH4	RIBC1	KCMF1	NDUFS1	GALNT10	CCK	HRH4
74	RSRC1	KPNA1	MEF2A	NWD1	CCK	PPM1K	RSRC1
75	DYRK1A	FOXP1	BRI3	CHRNA5	PPM1K	NEB	DYRK1A
76	JUN	HOOK1	ATXN1	EREG	PPBP	AMPH	JUN
77	FOXP2	ACTA1	MIS12	CCDC32	THAP5	SLC16A14	FOXP2
78	PIGK	TBC1D17	PAM	TMEM41B	VPS54	MBNL3	PIGK
79	LCE1C	HLF	C8orf44-SGK3	GUCY1A2	BIVM	GATAD1	LCE1C
80	FGF14	ATG7	SIRT1	RAB30	FLOT2	ZNF257	FGF14
81	OLA1	ASXL3	ALKAL1	WFDC13	TMEM120B	THAP5	OLA1
82	DENND5A	FAM13C	GRM3	L2HGDH	GATAD1	VPS54	DENND5A
83	FAM122C	MYBL1	SEPT8	LDLRAD2	ERBB4	BIVM	FAM122C
84	IFRD2	SACS	ENPP4	DLG1	NEB	TMEM120B	IFRD2
85	GK5	USP42	SLC26A7	TBC1D15	AMPH	PPBP	GK5
86	ELAVL2	DENND5B	FAM167A	TMEM56	SLC16A14	FLOT2	ELAVL2
87	ENPP3	CDH12	CAMSAP2	SULT6B1	ZNF257	ERBB4	ENPP3
88	RYR2	CSNK1A1L	KDM5A	PPFIA2	RPF1	RPF1	RYR2
89	LURAP1L	GRIK1	SOX11	MEX3A	MBNL3	LRRTM2	LURAP1L
90	KBTBD3	DDIT4	CELSR3	METTL17	LRP2	ZFYVE1	KBTBD3
91	PLPPR4	SOD2	COL11A1	FAM111A	ZNF676	LRP2	PLPPR4
92	UTS2B	WDR43	NLK	NCOA7	LRRTM2	ZNF676	UTS2B
93	WRAP73	PRKCE	DPYSL3	C2CD4A	ZFYVE1	MOCS2	WRAP73
94	CUL4B	G3BP1	KCNK2	SAMSN1	MOCS2	CNTF	CUL4B
95	MXI1	SH2B3	FBXO9	MFAP4	CNTF	SHCBP1	MXI1
96	PDK3	ADGRB3	SYN2	TRIP6	SHCBP1	ZNF493	PDK3
97	FREM1	ANAPC10	LIN28B	THAP3	CCL5	CPM	FREM1
98	ATP6V0A2	MPZ	CLMN	CHMP5	FLNA	LANCL1	ATP6V0A2
99	SYT11	LELP1	FAM91A1	SENP7	CXADR	FLNA	SYT11
100	AMMECR1L	TBC1D12	NREP	NFIA	ZNF367	BHLHE41	AMMECR1L
101	GSG1	GMFB	SEC16A	CCP110	ZNF493	MED1	GSG1
102	LAMP2	RAB8B	BOLL	ELAVL4	BHLHE41	CASP7	LAMP2
103	JOSD2	FOXO3	SALL4	SOX6	LANCL1	CCL5	JOSD2
104	ILDR2	ANKRD40	SALL1	ADAM9	MED1	CXADR	ILDR2
105	FBXL20	BRMS1L	ARID2	ZNF135	CPM	ZNF367	FBXL20
106	CXorf40A	SPTLC2	RAD54L2	CHST7	CASP7	FZD1	CXorf40A
107	ITGB8	RERG	SCN3A	SRD5A3	FZD1	SRD5A2	ITGB8
108	TMEM167A	SLC29A1	HNRNPM	KL	SRD5A2	MPHOSPH6	TMEM167A
109	ATRIP	WBP1L	ARMC8	VCPIP1	TOR1A	DCDC1	ATRIP
110	STRBP	HDAC4	SEMA6A	MAT2B	DCDC1	TOR1A	STRBP
111	RBBP9	ZKSCAN5	INPP5K	NFAT5	ZNF253	ZNF253	RBBP9
112	ANGPTL1	NOTCH2	LRRC58	F9	MPHOSPH6	HDAC8	ANGPTL1
113	OIP5	ELK4	CCDC71L	JPH1	ZNF136	ZNF662	OIP5
114	NAA25	UBE2A	CALU	BICD2	TRMT6	PHKB	NAA25
115	LSAMP	DBT	TMEM64	FGD2	SEMA3G	DTNA	LSAMP
116	MEF2D	ATP2B1	SPRED1	LPP	COLEC10	ZNF136	MEF2D

117	SPECC1	GPM6B	ADAMTS5	PCBD2	CYP27B1	SEMA3G	SPECC1
118	RNF212	TGFBR3	CACNG2	PTPN20	PHKB	TRMT6	RNF212
119	HCCS	PAX5	PSMD12	ATP8B1	DTNA	COLEC10	HCCS
120	TBL1XR1	ALCAM	GPD2	MMP16	ESYT2	CYP27B1	TBL1XR1
121	ALDH7A1	PDS5A	KRTAP4-1	HECTD2	HDAC8	ESYT2	ALDH7A1
122	SPESP1	RCN2	NFATC2	SYNPO2	ZNF662	AVL9	SPESP1
123	MEX3D	SMAD1	SRGAP1	CCL28	TMEM185B	TMEM185B	MEX3D
124	CALB1	PDLIM3	ARID1B	CDCA7	JAG1	CDKN2AIP	CALB1
125	EDC3	CALML4	ZHX1	NOVA1	AVL9	SIX4	EDC3
126	ATXN7L3B	RPS6KA6	PGM5	C8orf44-SGK3	GDNF	GDNF	ATXN7L3B
127	CXCR2	SEPT10	WDR5B	SGK3	CDKN2AIP	JAG1	CXCR2
128	FASTKD2	STAM	AZIN1	CAMK4	SIX4	MCTP2	FASTKD2
129	SNN	GTPBP8	RICTOR	FRYL	PAQR5	TSPYL1	SNN
130	NR4A1	CCKBR	ZNF451	CCR1	MCTP2	CNIH4	NR4A1
131	HNRNPF	RBM20	EP300	NUP43	MARCH6	ROR1	HNRNPF
132	SLC31A2	ACVR2A	SPATA13	FUT9	NPTN	MARCH6	SLC31A2
133	DMXL2	EBNA1BP2	CD300LF	SPAST	INO80D	KLF7	DMXL2
134	YIPF6	PRKCB	MED9	CALHM1	RGS5	PAQR5	YIPF6
135	ZEB1	MTSS1	CCDC34	ARSK	CCN4	INO80D	ZEB1
136	NEXMIF	SNCA	AMD1	MCTP1	TSPYL1	RGS5	NEXMIF
137	ZDHHC15	CLEC14A	CACNB1	UBE2W	CNIH4	CEP170B	ZDHHC15
138	SNRK	PHF20L1	GPATCH2L	KCNN3	ROR1	FAM210A	SNRK
139	C5orf51	RNF168	CBWD1	HNMT	CEP170B	NPTN	C5orf51
140	PDCD2	PKNOX1	SLC1A3	RFTN2	KLF7	CCN4	PDCD2
141	SP4	DESI2	TSPAN6	PREX2	FAM210A	CXorf40A	SP4
142	XBP1	TAOK3	SS18	HOXB5	KRTAP13-1	C6orf203	XBP1
143	CD93	PEX16	AGO1	MAPK10	PCSK6	ZNF436	CD93
144	LPP	STK39	GDF5	PROCR	C6orf203	WDFY4	LPP
145	CGA	HHEX	E2F5	C5orf34	CXorf40A	GALNT10	CGA
146	DUSP1	DUSP10	MUC13	PDZD8	ZNF436	KRTAP13-1	DUSP1
147	TMEM107	ZNF391	G3BP2	ZNF695	WDFY4	PCSK6	TMEM107
148	LARP1B	PHLPP1	NTNG1	ETV1	SLC38A2	SLC38A2	LARP1B
149	F13A1	ZNF706	KCNA6	IDS	PTPN1	CACNA2D1	F13A1
150	CDC14A	CTSV	KCNN3	SPATA2	CACNA2D1	MOB1B	CDC14A
151	KRTAP13-1	FUBP3	POM121	MBNL3	FAF2	PTPN1	KRTAP13-1
152	C9orf170	ABI2	FOXO3	TET2	LRRC15	FAF2	C9orf170
153	CAMSAP2	SLC39A1	H2AFZ	LONRF3	CCDC6	FAM122C	CAMSAP2
154	PLXNC1	PHIP	TRDN	HPS5	FAM122C	LRRC15	PLXNC1
155	PFN2	ICE2	DDX5	CYP7B1	HMBOX1	HMBOX1	PFN2
156	ANKRD46	MARCH3	TMEM136	ZNF703	TFCP2L1	TFCP2L1	ANKRD46
157	ZNF711	ZBTB26	SIX4	NCAN	MTR	PUM1	ZNF711
158	BCL11A	ZNF365	DYRK2	EDA2R	BZW1	MTR	BCL11A
159	GNAQ	LCORL	RPL31	SLC49A4	ITCH	ITCH	GNAQ
160	RHOBTB1	MEF2D	SLC30A6	DTD1	ZDHHC13	ZNF229	RHOBTB1
161	TGOLN2	PSIP1	TMEM178B	DHX35	INTS2	BZW1	TGOLN2
162	NCOA4	CAPRIN1	CBWD3	AMACR	PUM1	ZDHHC13	NCOA4
		CCDC96	ZYG11A	MDH1	ZNF229	INTS2	FBXW7
		ITPR3	TRNAU1AP	SETBP1	LFNG	SV2C	SP3
		DCAF12	RHOQ	CCDC13	SV2C	C7orf57	KPNA3
		RNF34	LSM11	USP14	POLR3A	B3GNT5	RRAS2
		METAP1	CBWD2	CLEC1A	C7orf57	LFNG	SLC4A4
		ZNF500	CNR1	PLCB1	RBPMS2	TRIP12	CRIM1
		SLC39A8	SIPA1L2	RGR	TRIP12	KCNMA1	ATP7A
		IFIH1	AEBP2	GLYAT	B3GNT5	PRCP	PAX6
		ATP5IF1	DIPK2A	MGAT3	ELAVL1	POLR3A	GALNT18
		ECT2	DCUN1D4	TRPC5	KCNMA1	RBPMS2	FOXO1
		FAM199X	KIAA2026	JARID2	PRCP	ELAVL1	SIAH1
		SYNCRIP	DUSP9	DYRK2	ROBO1	GJC1	F3

LRRC34	FAM76B	ERGIC2	GCFC2	DCAF7	UBR5
C5orf34	ATP10D	PSMA1	NUP58	CCDC89	PTBP2
RNF145	CC2D1B	ADGRB3	GJC1	ROBO1	INPP5B
GDPD1	EFCAB9	TERB2	DCAF7	GCFC2	CBX5
SMARCD1	GVQW2	ARL5A	RAB8B	NUP58	HSP90B1
SLC23A2	ADCY3	ZNF687	CCDC89	SLC1A1	ARMCX1
NMNAT2	AMER2	GRIK1	SLC1A1	SET	FBXO8
DLGAP1	FRS2	RWDD4	TRIM36	TRIM36	SLC35G2
UHMK1	MTMR10	CELF2	SET	UMPS	RHOB
MPP5	LRRFIP1	SH3BGR	UMPS	RAB8B	SEPT6
ICAM1	MAOA	AP3B1	ST7L	ST7L	ADCY7
CXXC4	INAFM2	SLC7A14	ZNF365	ZNF737	LMO2
IL6ST	ARNT	GABRA1	PIP5K1B	IER5L	KAT6A
STIM1	PBOV1	CCDC186	PTGS2	REEP5	RUBCNL
APC	ZBTB18	HOXB2	ZNF737	ZNF365	ACSL3
GPX8	TGS1	BLZF1	IER5L	CCDC6	FEZ2
PKP4	CASP7	HOXA13	REEP5	PIP5K1B	ANKS1B
MDK	EPB41L5	MCHR2	NSD1	PTGS2	HMGCS1
NFIC	MAP3K3	SCEL	ARL8A	XKR4	NDNF
JAKMIP2	STAG1	WRB-SH3BGR	FMNL3	GPX6	LSM8
RAP1GAP2	PYURF	QPRT	FBXO28	PRKCE	TENT5A
UBXN1	TMEM106B	NFXL1	ZDHHC7	GABRA1	HARBI1
ELP4	ZFP3	CNOT4	RBL1	NSD1	INPP4A
ATP1B1	MTMR1	KMT5A	RABGAP1	ARL8A	SLC8A1
RWDD4	HIC2	QRSL1	XKR4	FBXO28	SLC7A8
CSNK1A1	TTK	GASK1B	GPX6	RABGAP1	TMEM143
PAX9	ELOC	RECK	KDM2B	KDM2B	ZNF772
CNOT6L	CXorf40A	WDHD1	PRKCE	FMNL3	RBPJ
GUCY1A1	ATP6AP1L	PDLIM5	GABRA1	ZDHHC7	SMIM15
MTM1	ISL1	RASA2	SEPT14	RBL1	TWF1
SLC2A11	RFX3	PIAS2	NFAT5	SEPT14	FKBP5
GPR155	FMN1	CDC14B	TET2	TET2	LAMB1
CBLB	MAPKAP1	AK2	ZNF117	ZNF117	NFIB
PTPN2	HYDIN	ERICH4	IGSF1	KRT6B	INHBB
MID1IP1	FAM184A	CHIC1	MINDY2	NFAT5	LACC1
ANKRD17	NDUFAF6	KRAS	PHF20L1	PHF20L1	PARP1
RUBCN	FOXA1	KLHL32	KRT6B	FANCM	PDS5B
ZBTB18	CNIH1	ABCA5	FANCM	IGSF1	CDK17
MRPS17	USP9Y	SHISA3	DCAF17	MINDY2	KIAA1841
DNAJB13	PNKD	SLC2A11		DCAF17	RBSN
VNN1	SMAD2	SRFBP1			SDC2
ZCCHC14	MISP3	BDH2			RASA1
STRAP	EPM2AIP1	EGFL6			USP40
MINDY3	MCOLN3	GRM8			SLC37A3
TRIM37	PIK3R1	PTPN12			OLFM1
ANKS6	CERS2	RP2			SNX6
DTX4	PPP2CB	KAT2B			IGF1R
RASSF6	RTN4	EIF4A2			TMEM178B
FHL1	ARID4B	SF3A1			ARHGEF38
KL	PSMA2	CSF1			SORBS1
ERO1B	HAO1	DGKI			ZBTB42
RELCH	SLK	PWWP2A			MAEL
CYB5A	SDF2	IKZF2			C18orf54
ARHGAP44	APLP2	RBM41			TSPAN7
DIPK1A	TMEM47	AHR			NFIA
ZZZ3	DNAJC10	LRRTM3			MYO5B
KLHL14	MRM2	PHKB			PRDM1
PELI1	SEC62	HSD11B1			NUP210

TP53	HECTD1	FPGT	CYTIP
HAL	GRIP1	DCAF13	SREK1
RIF1	PPAT	ZNF33B	RIBC1
MYH10	C9orf72	FABP3	KPNA1
PEX13	ACSL4	WASHC4	FOXP1
DCUN1D3	TRIM2	GOSR1	HOOK1
ARL13B	RPL13A	REPS2	ACTA1
PURA	TC2N	SP5	TBC1D17
UBR1	HAPLN1	MAATS1	HLF
NXF1	DYNC1LI2	PAX2	ATG7
MMP16	MINDY3	ABCD3	ASXL3
CEACAM5	TMEFF1	PATE1	FAM13C
ZBTB40	LZTS3	CHURC1	MYBL1
	C22orf39	TMEM33	SACS
	KIAA1211L	SLC41A2	USP42
	ABHD5	PEX13	DENND5B
	SRP72	HYDIN	CDH12
	ATXN7	PPP1R10	CSNK1A1L
	BCAN	FGG	GRIK1
	NFAT5	PMS1	DDIT4
	ANP32A	ARID2	SOD2
	PHF20L1	TCHH	WDR43
	PRDM15	FMC1-LUC7L2	PRKCE
	SLBP	LUC7L2	G3BP1
	KLHL11	DLL4	SH2B3
	HRNR	SSBP3	ADGRB3
	HEXB	C22orf39	ANAPC10
	PRPF4B	TRAF6	MPZ
	PDE7A	TRDN	LELP1
	WDCP	UTRN	TBC1D12
	CNTNAP3B	VWA8	GMFB
	SCN1A	PRPF8	RAB8B
	KCNH5	SEMA6D	FOXO3
	MEX3A	CSF2RA	ANKRD40
	SOS1	JPT2	BRMS1L
	BICD2	EPOR	SPTLC2
	TMEM41B	C21orf91	RERG
	PRDM6	ZIC2	SLC29A1
	DENND1B	PF4	WBP1L
	FANK1	GMPPB	HDAC4
	COL5A2	FNDC9	ZKSCAN5
	HS2ST1	TEP1	NOTCH2
	PRKD3	APOPT1	ELK4
	SLC38A10	THRB	UBE2A
	GNA12	PTGS2	DBT
	PBDC1	HERC3	ATP2B1
	EPC1	EFR3A	GPM6B
	MAP3K20	GINS1	TGFBR3
	MFSD11	PRAMEF2	PAX5
	MECP2	AIMP1	ALCAM
	DNAJA2	BCAP29	PDS5A
	CNTNAP3	DENND6A	RCN2
	ZNF236	KIF14	SMAD1
	EIF4A2	NRG1	PDLIM3
	SOWAHA	ICK	CALML4
	RAD21	VPS13C	RPS6KA6
	PPM1E	CTNNA3	SEPT10
	EGR1	KLHL7	STAM

SFMBT1	LRRC74B	GTPBP8
PTAR1	UBA3	CCKBR
SLC20A1	MBLAC2	RBM20
BCL2L11	MED11	ACVR2A
RAB6B	ANTXR2	EBNA1BP2
NEUROD4	ODR4	PRKCB
CAPRIN1	RGS5	MTSS1
NCKAP5	PGAM4	SNCA
OPRM1	CACNB4	CLEC14A
TET2	P2RY12	PHF20L1
SEPHS1	ZBTB20	RNF168
SMAD5	ZNF333	PKNOX1
PAIP2	CALHM3	DESI2
FBXO28	SLC16A7	TAOK3
TSPYL5	KBTBD6	PEX16
AMOT	IL7	STK39
YIPF5	PLAG1	HHEX
RAB28	LTN1	DUSP10
ATL3	PRKCA	ZNF391
SEMA4G	FSIP1	PHLPP1
RNASEH2B	GPBP1	ZNF706
SRGAP2B	PHF23	CTSV
PKD1L1	SLIT2	FUBP3
CENPQ	ARID1A	ABI2
KITLG	ANTXR1	SLC39A1
WT1	FAM8A1	PHIP
REEP3	DLK1	ICE2
MTPN	FAR2	MARCH3
PTGS2	TMEM65	ZBTB26
UBE2D3	EXD2	ZNF365
SEC14L2	SLC26A7	LCORL
ZNF644	ELK4	MEF2D
CCDC169	ELAVL3	PSIP1
GPBP1	EPGN	CAPRIN1
PTPRD	ZBTB7C	CCDC96
CLDN16	FAXC	ITPR3
UHMK1	UBN2	DCAF12
MTCL1	PTPN4	RNF34
ADCYAP1	B4GALT4	METAP1
UNC13A	PPP6R3	ZNF500
GABRG1	GABRA5	SLC39A8
CTCFL	ZIC1	IFIH1
ARHGEF40	MEF2D	ATP5IF1
BTBD1	ZKSCAN8	ECT2
ARHGAP32	HMCN1	FAM199X
C18orf25	TCP10L2	SYNCRIP
FZD6	ST8SIA3	LRRC34
ANKRD29	CYS1	C5orf34
	GRIN2A	RNF145
	TGFBI	GDPD1
	CPEB4	SMARCD1
	L3MBTL4	SLC23A2
	SGPP1	NMNAT2
	IAH1	DLGAP1
	ARL13B	UHMK1
	PPIF	MPP5
	CD2AP	ICAM1
	TRMT10A	CXXC4
	COLEC12	IL6ST

C17orf75	STIM1
DCDC2	APC
PNLIPRP3	GPX8
FAM199X	PKP4
IFI44	MDK
LARP4	NFIC
ZNF10	JAKMIP2
MOG	RAP1GAP2
PGC	UBXN1
CUBN	ELP4
OSTF1	ATP1B1
MSR1	RWDD4
PPARGC1A	CSNK1A1
COL11A1	PAX9
DAPP1	CNOT6L
NEGR1	GUCY1A1
KRT78	MTM1
SMCHD1	SLC2A11
WNT3	GPR155
USP12	CBLB
NACC2	PTPN2
LETM2	MID1IP1
GSK3B	ANKRD17
STX17	RUBCN
CDK19	ZBTB18
FAM129A	MRPS17
PEX5L	DNAJB13
TGFBR1	VNN1
RSPO3	ZCCHC14
DNAJB4	STRAP
LRRC3B	MINDY3
TANK	TRIM37
PAN3	ANKS6
LIN9	DTX4
GRB2	RASSF6
DKK1	FHL1
CTBS	KL
SPRY4	ERO1B
INAFM2	RELCH
MAP3K13	CYB5A
WASHC3	ARHGAP44
EIF3J	DIPK1A
LRCH2	ZZZ3
CALCB	KLHL14
KPNA4	PELI1
UNC5D	TP53
DMBX1	HAL
KCNV1	RIF1
UGT2B17	MYH10
MAN1A1	PEX13
NXT2	DCUN1D3
KIAA1549L	ARL13B
GPSM1	PURA
SLITRK4	UBR1
ODAPH	NXF1
RGS4	MMP16
ITGB6	CEACAM5
TAOK3	ZBTB40
NLK	CDK19

ZBTB18	TIMM9
ACVR2B	LEMD3
CREB1	SOX5
ENOX2	CBLL1
ADCYAP1	MIA3
PRAMEF13	RGS7BP
GLE1	MIER1
TMX4	METTL25
FAM78A	MAPK1
RAB31	GTF2H1
WDR64	RASA1
PPP1R1C	MEX3C
PRAMEF14	TUT4
RSBN1	SGK3
NHLRC2	CHD1
PRAMEF1	USP38
ADGRG2	DAZAP2
ZDHHC20	VDAC2
CAVIN2	MELK
DPY19L2	FEM1C
KMT2A	SSH2
NDUFAF3	BRWD1
LRRC8D	ZBTB20
HPSE	CSDE1
DICER1	MYCBP2
OCLN	HBEGF
IMPAD1	CCDC88A
MLPH	PTBP2
EPS15	MTF2
UHRF1BP1L	ZNF521
CA1	SOD2
RXFP1	SETD5
XIRP2	SERP1
PLIN1	NFE2L2
RTN4RL1	KLF7
FRMPD4	OSBPL8
USP10	ZNF652
SLC19A2	TJAP1
PRXL2A	ETNK1
ZNF33A	PCDH10
MORC1	ZNF516
MMS22L	DAAM1
PCDH7	HHIP
HPN	SLC6A1
UBE3C	NOVA1
C6orf136	CCN2
AHCTF1	NMNAT2
COMMD10	SPPL3
WDR5B	HNRNPH1
BRAT1	FBXL20
CHMP3	SKAP2
MSRB3	TLN2
TAF9	GMFB
GTF2F1	SAP30L
SLC6A15	PPM1G
ANKRD20A1	SLC25A28
HMGXB4	ELMSAN1
SNRPN	NVL
LONRF2	RUFY3

CCDC149	RPP14
TNFSF4	DCC
SOGA1	L3MBTL3
CCNT2	FAM227A
NF1	ASF1A
AASDHPPT	PHF12
ANKRD20A4	USP9X
RNF103-CHMP3	STX16
POFUT1	RB1
MINDY2	ACVR2B
DUSP28	NACC2
MAP3K7	TMEM164
ANKRD20A2	KCMF1
C21orf62	MEF2A
RASEF	BRI3
NXT1	ATXN1
SMS	MIS12
REG3G	PAM
MINAR1	C8orf44-SGK3
CHUK	SIRT1
STARD4	ALKAL1
CLEC12B	GRM3
VGLL1	SEPT8
CCDC14	ENPP4
CDK13	SLC26A7
IFFO1	FAM167A
STRN	CAMSAP2
AKAP4	KDM5A
GATA3	SOX11
ZFAND5	CELSR3
CASP3	COL11A1
STK17B	NLK
PHC3	DPYSL3
TMEM266	KCNK2
LMO4	FBXO9
C2orf69	SYN2
TOB1	LIN28B
NR1D2	CLMN
SFRP4	FAM91A1
NUDT12	NREP
PHLPP1	SEC16A
NHSL1	BOLL
IL17D	SALL4
ASCC3	SALL1
BAIAP2L1	ARID2
FGF2	RAD54L2
SELENOT	SCN3A
LACC1	HNRNPM
LOC100130451	ARMC8
TFEC	SEMA6A
POLR2K	INPP5K
ASTN1	LRRC58
GAS2L3	CCDC71L
TMEM209	CALU
GALNT4	TMEM64
MED14	SPRED1

FKTN	ADAMTS5
KLF17	CACNG2
AFG1L	PSMD12
GRIA2	GPD2
USP47	KRTAP4-1
ZRANB1	NFATC2
SSX2IP	SRGAP1
POC1B-	
GALNT4	ARID1B
SYS1	ZHX1
DAP3	PGM5
NSL1	WDR5B
NCAPH	AZIN1
CADM2	RICTOR
C16orf72	ZNF451
CEP126	EP300
SLC35A3	SPATA13
BMP3	CD300LF
FCHO2	MED9
TMEM59	CCDC34
CRBN	AMD1
CADM1	CACNB1
DBR1	GPATCH2L
C11orf87	CBWD1
ST8SIA4	SLC1A3
NAPG	TSPAN6
UPRT	SS18
CACNA1A	AGO1
SH3BGRL2	GDF5
ACER3	E2F5
EYA4	MUC13
SLC25A15	G3BP2
LRCH1	NTNG1
CEP170	KCNA6
GPATCH2L	KCNN3
PLEKHH2	POM121
BTC	FOXO3
RASSF6	H2AFZ
MAP2	TRDN
PTPN22	DDX5
SMIM11B	TMEM136
C10orf126	SIX4
HOMEZ	DYRK2
FBN2	RPL31
GLIPR1	SLC30A6
RO60	TMEM178B
ATP1A2	CBWD3
CERS6	ZYG11A
SOX13	TRNAU1AP
C9orf72	RHOQ
SDHAF3	LSM11
ZKSCAN4	CBWD2
IL36G	CNR1
GFRAL	SIPA1L2
TRIL	AEBP2
GRM7	DIPK2A
SMIM11A	DCUN1D4
ZNF148	KIAA2026
MYBL1	DUSP9

CRLS1	FAM76B
EIF4A3	ATP10D
PLEKHB2	CC2D1B
HOOK3	EFCAB9
IREB2	GVQW2
CIAO2A	ADCY3
TNS2	AMER2
TMTC1	FRS2
SS18L1	MTMR10
EPHA5	LRRFIP1
CPED1	MAOA
CDH20	INAFM2
MSH4	ARNT
MDGA2	PBOV1
FXR1	ZBTB18
GNL1	TGS1
NCK1	CASP7
GUCY1A1	EPB41L5
HSPA4L	MAP3K3
MARCH3	STAG1
PPP1R12B	PYURF
PDZRN4	TMEM106B
IFT57	ZFP3
GRM5	MTMR1
KIRREL2	HIC2
ZBTB1	TTK
POLK	ELOC
ZNF777	CXorf40A
TCTN1	ATP6AP1L
RABGEF1	ISL1
FEM1B	RFX3
PTPN14	FMN1
NUDT7	MAPKAP1
TTC7B	HYDIN
HELZ	FAM184A
MYOZ3	NDUFAF6
SETD5	FOXA1
GHR	CNIH1
SMIM8	USP9Y
RBM19	PNKD
RPRD1A	SMAD2
FYB1	MISP3
EFCAB7	EPM2AIP1
PANK3	MCOLN3
TSHZ1	PIK3R1
ZNF197	CERS2
RCN2	PPP2CB
DIP2B	RTN4
ESM1	ARID4B
UBIAD1	PSMA2
ABHD18	HAO1
E2F7	SLK
WIPF2	SDF2
ZNF208	APLP2
TNFAIP2	TMEM47
CHAMP1	DNAJC10
PCDH10	MRM2
VAPA	SEC62
PF4V1	HECTD1

ZNF624	GRIP1
ADAMTSL1	PPAT
DDIAS	C9orf72
MYD88	ACSL4
ASAH2B	TRIM2
DBF4	RPL13A
PHACTR2	TC2N
ARHGAP20	HAPLN1
DCAF10	DYNC1LI2
SYT14	MINDY3
MRTFB	TMEFF1
WWC1	LZTS3
NECAB1	C22orf39
EFCAB11	KIAA1211L
EVI2A	ABHD5
EXOC5	SRP72
ASPH	ATXN7
SLC25A30	BCAN
SNAI2	NFAT5
SRGAP2B	ANP32A
ANO5	PHF20L1
METTL15	PRDM15
DNAJC3	SLBP
MBOAT1	KLHL11
PUS10	HRNR
ANGPTL1	HEXB
JAG1	PRPF4B
LRRC55	PDE7A
ZNF224	WDCP
SNURF	CNTNAP3B
ZNF680	SCN1A
PPP2R2B	KCNH5
C3orf80	MEX3A
LAMTOR3	SOS1
DPH6	BICD2
GCLM	TMEM41B
NLGN4Y	PRDM6
GJB2	DENND1B
SMIM25	FANK1
ITGB3BP	COL5A2
PTPRB	HS2ST1
TGS1	PRKD3
SNX14	SLC38A10
KCNMA1	GNA12
OGT	PBDC1
MPC1	EPC1
COL12A1	MAP3K20
RUNX1T1	MFSD11
DAB2IP	MECP2
FMO2	DNAJA2
AP1G1	CNTNAP3
TMCO4	ZNF236
BAG3	EIF4A2
GPR88	SOWAHA
COBLL1	RAD21
BARD1	PPM1E
ABRA	EGR1
CHCHD3	SFMBT1
HRH4	PTAR1

ADAMTS6	SLC20A1
ZNF354C	BCL2L11
ZNF407	RAB6B
ANK3	NEUROD4
EBPL	CAPRIN1
LDB1	NCKAP5
C2	OPRM1
RBM26	TET2
SREK1	SEPHS1
AGTR2	SMAD5
STC1	PAIP2
CHD1L	FBXO28
TSG101	TSPYL5
SPOUT1	AMOT
MIPOL1	YIPF5
ATG3	RAB28
SPATA6	ATL3
RAB11FIP2	SEMA4G
DOCK7	RNASEH2B
BRAF	SRGAP2B
MCTS1	PKD1L1
TRAPPC13	CENPQ
PCSK2	KITLG
PCLO	WT1
SCAP	REEP3
SLC7A4	MTPN
PALM2	PTGS2
ALDH1L2	UBE2D3
AKIRIN1	SEC14L2
MRPL57	ZNF644
POU2F1	CCDC169
AASS	GPBP1
CALCRL	PTPRD
CNEP1R1	CLDN16
RAB22A	UHMK1
DNAH5	MTCL1
PCSK5	ADCYAP1
PPAT	UNC13A
NR3C1	GABRG1
GLCE	CTCFL
NEUROG2	ARHGEF40
RC3H1	BTBD1
PTPN11	ARHGAP32
MCM5	C18orf25
CCDC179	FZD6
TTC9	ANKRD29
GPATCH8	HSPB8
CDYL2	PDE7B
	FBXO6
	TRMT9B
	TNFAIP8L3
	HOXB6
	GNE
	NAT1
	CASP9
	TRIM8
	TMEM182
	MR1
	ESRRG

S1PR3
A2ML1
MDM4
BRWD3
RFX4
TRPS1
ARL11
FAM168A
CASK
DENND1B
GRIK2
TBCA
HOXC8
INPP5D
CAMK2A
GABRA4
CD84
GGT6
TSC22D4
MACC1
GABRB2
RORA
MAP3K2
FGF7
CNTN1
SETD9
CHST11
PI15
VPS13A
YIPF6
PNRC1
MYEF2
KCNB1
COMMD2
SHISAL1
FXN
TUBGCP4
MMRN1
ZDHHC15
PKHD1
KIRREL1
STEAP2
RABL3
EBF1
ZIC5
DBNDD2
C5orf47
ZBTB41
SLC25A53
TSHZ3
TTF2
AMFR
SRSF12
TECPR2
ACAN
SYT10
ADH4
VWC2
RINL

NDUFS1
NWD1
CHRNA5
EREG
CCDC32
TMEM41B
GUCY1A2
RAB30
WFDC13
L2HGDH
LDLRAD2
DLG1
TBC1D15
TMEM56
SULT6B1
PPFIA2
MEX3A
METTL17
FAM111A
NCOA7
C2CD4A
SAMSN1
MFAP4
TRIP6
THAP3
CHMP5
SENP7
NFIA
CCP110
ELAVL4
SOX6
ADAM9
ZNF135
CHST7
SRD5A3
KL
VCPIP1
MAT2B
NFAT5
F9
JPH1
BICD2
FGD2
LPP
PCBD2
PTPN20
ATP8B1
MMP16
HECTD2
SYNPO2
CCL28
CDCA7
NOVA1
C8orf44-
SGK3
SGK3
CAMK4
FRYL
CCR1

NUP43
FUT9
SPAST
CALHM1
ARSK
MCTP1
UBE2W
KCNN3
HNMT
RFTN2
PREX2
HOXB5
MAPK10
PROCR
C5orf34
PDZD8
ZNF695
ETV1
IDS
SPATA2
MBNL3
TET2
LONRF3
HPS5
CYP7B1
ZNF703
NCAN
EDA2R
SLC49A4
DTD1
DHX35
AMACR
MDH1
SETBP1
CCDC13
USP14
CLEC1A
PLCB1
RGR
GLYAT
MGAT3
TRPC5
JARID2
DYRK2
ERGIC2
PSMA1
ADGRB3
TERB2
ARL5A
ZNF687
GRIK1
RWDD4
CELF2
SH3BGR
AP3B1
SLC7A14
GABRA1
CCDC186
HOXB2

BLZF1
HOXA13
MCHR2
SCEL
WRB-
SH3BGR
QPRT
NFXL1
CNOT4
KMT5A
QRSL1
GASK1B
RECK
WDHD1
PDLIM5
RASA2
PIAS2
CDC14B
AK2
ERICH4
CHIC1
KRAS
KLHL32
ABCA5
SHISA3
SLC2A11
SRFBP1
BDH2
EGFL6
GRM8
PTPN12
RP2
KAT2B
EIF4A2
SF3A1
CSF1
DGKI
PWWP2A
IKZF2
RBM41
AHR
LRRTM3
PHKB
HSD11B1
FPGT
DCAF13
ZNF33B
FABP3
WASHC4
GOSR1
REPS2
SP5
MAATS1
PAX2
ABCD3
PATE1
CHURC1
TMEM33
SLC41A2

PEX13
HYDIN
PPP1R10
FGG
PMS1
ARID2
TCHH
FMC1-
LUC7L2
LUC7L2
DLL4
SSBP3
C22orf39
TRAF6
TRDN
UTRN
VWA8
PRPF8
SEMA6D
CSF2RA
JPT2
EPOR
C21orf91
ZIC2
PF4
GMPPB
FNDC9
TEP1
APOPT1
THRB
PTGS2
HERC3
EFR3A
GINS1
PRAMEF2
AIMP1
BCAP29
DENND6A
KIF14
NRG1
ICK
VPS13C
CTNNA3
KLHL7
LRRC74B
UBA3
MBLAC2
MED11
ANTXR2
ODR4
RGS5
PGAM4
CACNB4
P2RY12
ZBTB20
ZNF333
CALHM3
SLC16A7
KBTBD6

IL7
PLAG1
LTN1
PRKCA
FSIP1
GPBP1
PHF23
SLIT2
ARID1A
ANTXR1
FAM8A1
DLK1
FAR2
TMEM65
EXD2
SLC26A7
ELK4
ELAVL3
EPGN
ZBTB7C
FAXC
UBN2
PTPN4
B4GALT4
PPP6R3
GABRA5
ZIC1
MEF2D
ZKSCAN8
HMCN1
TCP10L2
ST8SIA3
CYS1
GRIN2A
TGFBI
CPEB4
L3MBTL4
SGPP1
IAH1
ARL13B
PPIF
CD2AP
TRMT10A
COLEC12
C17orf75
DCDC2
PNLIPRP3
FAM199X
IFI44
LARP4
ZNF10
MOG
PGC
CUBN
OSTF1
MSR1
PPARGC1A
COL11A1
DAPP1

NEGR1
KRT78
SMCHD1
WNT3
USP12
NACC2
LETM2
GSK3B
STX17
CDK19
FAM129A
PEX5L
TGFBR1
RSPO3
DNAJB4
LRRC3B
TANK
PAN3
LIN9
GRB2
DKK1
CTBS
SPRY4
INAFM2
MAP3K13
WASHC3
EIF3J
LRCH2
CALCB
KPNA4
UNC5D
DMBX1
KCNV1
UGT2B17
MAN1A1
NXT2
KIAA1549L
GPSM1
SLITRK4
ODAPH
RGS4
ITGB6
TAOK3
NLK
ZBTB18
ACVR2B
CREB1
ENOX2
ADCYAP1
PRAMEF13
GLE1
TMX4
FAM78A
RAB31
WDR64
PPP1R1C
PRAMEF14
RSBN1
NHLRC2

PRAMEF1
ADGRG2
ZDHHC20
CAVIN2
DPY19L2
KMT2A
NDUFAF3
LRRC8D
HPSE
DICER1
OCLN
IMPAD1
MLPH
EPS15
UHRF1BP1L
CA1
RXFP1
XIRP2
PLIN1
RTN4RL1
FRMPD4
USP10
SLC19A2
PRXL2A
ZNF33A
MORC1
MMS22L
PCDH7
HPN
UBE3C
C6orf136
AHCTF1
COMMD10
WDR5B
BRAT1
CHMP3
MSRB3
TAF9
GTF2F1
SLC6A15
ANKRD20A1
HMGXB4
SNRPN
LONRF2
CCDC149
TNFSF4
SOGA1
CCNT2
NF1
AASDHPPT
ANKRD20A4
RNF103-
CHMP3
POFUT1
MINDY2
DUSP28
MAP3K7
ANKRD20A2
C21orf62

RASEF
NXT1
SMS
REG3G
MINAR1
CHUK
STARD4
CLEC12B
VGLL1
CCDC14
CDK13
IFFO1
STRN
AKAP4
GATA3
ZFAND5
CASP3
STK17B
PHC3
TMEM266
LMO4
C2orf69
TOB1
NR1D2
SFRP4
NUDT12
PHLPP1
NHSL1
IL17D
ASCC3
BAIAP2L1
FGF2
SELENOT
LACC1
LOC10013045
1
TFEC
POLR2K
ASTN1
GAS2L3
TMEM209
GALNT4
MED14
FKTN
KLF17
AFG1L
GRIA2
USP47
ZRANB1
SSX2IP
POC1B-
GALNT4
SYS1
DAP3
NSL1
NCAPH
CADM2
C16orf72
CEP126

SLC35A3
BMP3
FCHO2
TMEM59
CRBN
CADM1
DBR1
C11orf87
ST8SIA4
NAPG
UPRT
CACNA1A
SH3BGRL2
ACER3
EYA4
SLC25A15
LRCH1
CEP170
GPATCH2L
PLEKHH2
BTC
RASSF6
MAP2
PTPN22
SMIM11B
C10orf126
HOMEZ
FBN2
GLIPR1
RO60
ATP1A2
CERS6
SOX13
C9orf72
SDHAF3
ZKSCAN4
IL36G
GFRAL
TRIL
GRM7
SMIM11A
ZNF148
MYBL1
CRLS1
EIF4A3
PLEKHB2
HOOK3
IREB2
CIAO2A
TNS2
TMTC1
SS18L1
EPHA5
CPED1
CDH20
MSH4
MDGA2
FXR1
GNL1

NCK1
GUCY1A1
HSPA4L
MARCH3
PPP1R12B
PDZRN4
IFT57
GRM5
KIRREL2
ZBTB1
POLK
ZNF777
TCTN1
RABGEF1
FEM1B
PTPN14
NUDT7
TTC7B
HELZ
MYOZ3
SETD5
GHR
SMIM8
RBM19
RPRD1A
FYB1
EFCAB7
PANK3
TSHZ1
ZNF197
RCN2
DIP2B
ESM1
UBIAD1
ABHD18
E2F7
WIPF2
ZNF208
TNFAIP2
CHAMP1
PCDH10
VAPA
PF4V1
ZNF624
ADAMTSL1
DDIAS
MYD88
ASAH2B
DBF4
PHACTR2
ARHGAP20
DCAF10
SYT14
MRTFB
WWC1
NECAB1
EFCAB11
EVI2A
EXOC5

ASPH
SLC25A30
SNAI2
SRGAP2B
ANO5
METTL15
DNAJC3
MBOAT1
PUS10
ANGPTL1
JAG1
LRRC55
ZNF224
SNURF
ZNF680
PPP2R2B
C3orf80
LAMTOR3
DPH6
GCLM
NLGN4Y
GJB2
SMIM25
ITGB3BP
PTPRB
TGS1
SNX14
KCNMA1
OGT
MPC1
COL12A1
RUNX1T1
DAB2IP
FMO2
AP1G1
TMCO4
BAG3
GPR88
COBLL1
BARD1
ABRA
CHCHD3
HRH4
ADAMTS6
ZNF354C
ZNF407
ANK3
EBPL
LDB1
C2
RBM26
SREK1
AGTR2
STC1
CHD1L
TSG101
SPOUT1
MIPOL1
ATG3

SPATA6
RAB11FIP2
DOCK7
BRAF
MCTS1
TRAPPC13
PCSK2
PCLO
SCAP
SLC7A4
PALM2
ALDH1L2
AKIRIN1
MRPL57
POU2F1
AASS
CALCRL
CNEP1R1
RAB22A
DNAH5
PCSK5
PPAT
NR3C1
GLCE
NEUROG2
RC3H1
PTPN11
MCM5
CCDC179
TTC9
GPATCH8
CDYL2
TRAF6
IRAK1
SEC23IP
NOVA1
PPP1R11
UPP2
WWC2
BCORL1
ZNF649
SORT1
NUMB
USP32
PLSCR4
CARD10
SRSF6
APPL1
ZNF652
SIAH2
GDAP1L1
SLC10A3
MRS2
ZNF354B
C9orf72
EIF4G2
ZNF540
GRIA3
LOC388813

DDHD1
BRK1
TDRKH
ZBTB2
CD80
FBXW2
POFUT2
NEMP1
DCAF12
CXXC4
KCNJ16
USP6
ABL2
HNRNPD
MMP16
RHOBTB3
CTAGE9
HIPK3
RARB
FOXR2
CTAGE8
CTAGE4
SRP72
VASN
FBXO4
SLC38A1
ZNF275
SLC12A6
TMEM19
ZNF90
NOS1
ZNF506
GPM6B
LRCH1
C3orf38
MAP3K8
C8orf88
YWHAB
TCF20
CD96
MED20
YES1
ARL10
STRBP
PTPRA
GALNT10
CCK
PPM1K
PPBP
THAP5
VPS54
BIVM
FLOT2
TMEM120B
GATAD1
ERBB4
NEB
AMPH
SLC16A14

ZNF257
RPF1
MBNL3
LRP2
ZNF676
LRRTM2
ZFYVE1
MOCS2
CNTF
SHCBP1
CCL5
FLNA
CXADR
ZNF367
ZNF493
BHLHE41
LANCL1
MED1
CPM
CASP7
FZD1
SRD5A2
TOR1A
DCDC1
ZNF253
MPHOSPH6
ZNF136
TRMT6
SEMA3G
COLEC10
CYP27B1
PHKB
DTNA
ESYT2
HDAC8
ZNF662
TMEM185B
JAG1
AVL9
GDNF
CDKN2AIP
SIX4
PAQR5
MCTP2
MARCH6
NPTN
INO80D
RGS5
CCN4
TSPYL1
CNIH4
ROR1
CEP170B
KLF7
FAM210A
KRTAP13-1
PCSK6
C6orf203
CXorf40A

ZNF436
WDFY4
SLC38A2
PTPN1
CACNA2D1
FAF2
LRRC15
CCDC6
FAM122C
HMBOX1
TFCP2L1
MTR
BZW1
ITCH
ZDHHC13
INTS2
PUM1
ZNF229
LFNG
SV2C
POLR3A
C7orf57
RBPMS2
TRIP12
B3GNT5
ELAVL1
KCNMA1
PRCP
ROBO1
GCFC2
NUP58
GJC1
DCAF7
RAB8B
CCDC89
SLC1A1
TRIM36
SET
UMPS
ST7L
ZNF365
PIP5K1B
PTGS2
ZNF737
IER5L
REEP5
NSD1
ARL8A
FMNL3
FBXO28
ZDHHC7
RBL1
RABGAP1
XKR4
GPX6
KDM2B
PRKCE
GABRA1
SEPT14

NFAT5
TET2
ZNF117
IGSF1
MINDY2
PHF20L1
KRT6B
FANCM
DCAF17
TRAF6
IRAK1
SEC23IP
NOVA1
UPP2
WWC2
PPP1R11
BCORL1
SORT1
ZNF649
NUMB
USP32
PLSCR4
GDAP1L1
SIAH2
SRSF6
APPL1
ZNF652
CARD10
MRS2
EIF4G2
ZNF540
SLC10A3
C9orf72
ZNF354B
GRIA3
DDHD1
LOC388813
TDRKH
BRK1
NEMP1
CD80
DCAF12
ZBTB2
CXXC4
FBXW2
POFUT2
ABL2
KCNJ16
USP6
HNRNPD
MMP16
HIPK3
CTAGE4
CTAGE9
CTAGE8
RHOBTB3
RARB
FOXR2
SLC38A1

SRP72
VASN
FBXO4
TMEM19
GPM6B
C3orf38
C8orf88
YWHAB
SLC12A6
ZNF90
NOS1
ZNF275
ZNF506
LRCH1
MAP3K8
MED20
YES1
TCF20
CD96
ARL10
STRBP
PTPRA
CCK
PPM1K
NEB
AMPH
SLC16A14
MBNL3
GATAD1
ZNF257
THAP5
VPS54
BIVM
TMEM120B
PPBP
FLOT2
ERBB4
RPF1
LRRTM2
ZFYVE1
LRP2
ZNF676
MOCS2
CNTF
SHCBP1
ZNF493
CPM
LANCL1
FLNA
BHLHE41
MED1
CASP7
CCL5
CXADR
ZNF367
FZD1
SRD5A2
MPHOSPH6
DCDC1

TOR1A
ZNF253
HDAC8
ZNF662
PHKB
DTNA
ZNF136
SEMA3G
TRMT6
COLEC10
CYP27B1
ESYT2
AVL9
TMEM185B
CDKN2AIP
SIX4
GDNF
JAG1
MCTP2
TSPYL1
CNIH4
ROR1
MARCH6
KLF7
PAQR5
INO80D
RGS5
CEP170B
FAM210A
NPTN
CCN4
CXorf40A
C6orf203
ZNF436
WDFY4
GALNT10
KRTAP13-1
PCSK6
SLC38A2
CACNA2D1
MOB1B
PTPN1
FAF2
FAM122C
LRRC15
HMBOX1
TFCP2L1
PUM1
MTR
ITCH
ZNF229
BZW1
ZDHHC13
INTS2
SV2C
C7orf57
B3GNT5
LFNG
TRIP12

KCNMA1
PRCP
POLR3A
RBPMS2
ELAVL1
GJC1
DCAF7
CCDC89
ROBO1
GCFC2
NUP58
SLC1A1
SET
TRIM36
UMPS
RAB8B
ST7L
ZNF737
IER5L
REEP5
ZNF365
CCDC6
PIP5K1B
PTGS2
XKR4
GPX6
PRKCE
GABRA1
NSD1
ARL8A
FBXO28
RABGAP1
KDM2B
FMNL3
ZDHHC7
RBL1
SEPT14
TET2
ZNF117
KRT6B
NFAT5
PHF20L1
FANCM
IGSF1
MINDY2
DCAF17
