# Supplementary material for: Exploring Neuronal Exosome miRNAs as Biomarkers of Neuroinflammation and Neuroplasticity in Amateur Boxers After Repetitive Head Trauma
Source: Mol Neurobiol. 2026 Mar 15;63(1):501. doi: 10.1007/s12035-026-05799-8 (PMC12989019; doi:10.1007/s12035-026-05799-8)
Supplement: Supplementary file 3 — (324 KB PDF) [file 12035_2026_5799_MOESM3_ESM.pdf]

BP

Term	P-value	Adjusted P-	Genes
Regulation of DNA-templated Transcription (GO:0006355)	1,14E+07	2,42E+10	RB1;EHMT1;IKZF2;RBPJ;ELK4;GPBP1;EPC1;SOX6;SOX5;PRKCB;SFMBT1;ZFP3;SOX13;EBF1;ZNF10;RFX3;EREG;RUNX1;RFX4;ZNF711;HOXB2;TP53;HOXB6;HOXB5;ZNF275;L3MBTL3;SET;TSHZ3;ZNF391;TSHZ1;IGSF1;PRDM15;GATA3;NLK;SEC14L2;ZNF706;RAD21;MIER1;ZNF703;INPP5K;ZNF148;PPARGC1A;HOXC8;ZBTB7C;SNX6;BARD1;FZD1;EGR1;JUN;XBP1;BCL11A;SS18L1;NFATC2;NR1D2;PHC3;ZZZ3;FUBP3;TNFSF4;HNRNPD;SNAI2;ZNF257;ZNF136;ZNF135;CC2D1B;FANK1;SETD5;ZNF253;ZNF493;BHLHE41;MIA3;TCF20;TFCP2L1;MECP2;DMBX1;MED14;ELOC;ZNF367;SS18;TSC22D4;PARP1;ZNF480;PAX6;PAX5;ZHX1;SIRT1;PAX2;PHF20L1;KAT2B;ZEB1;AEBP2;MED20;PAX9;TFEC;NCOA7;ZNF117;ZNF236;PLCB1;VGLL1;YAP1;PURA;NSD1;ZNF229;ZNF224;RUNX1T1;DAB2IP;GTF2H1;ELP4;ZNF33B;ZNF33A;NFIA;WT1;NFIB;CAMK4;ZNF333;ZNF354C;ZNF695;ZNF354B;FOXA1;WWC1;CD80;WWC2;RORA;ARID4B;PRDM1;AHR;NR3C1;TNF;HOXA13;SALL1;POFUT1;SALL4;ZNF208;MAEL;EP300;SLC39A8;KMT5A;ARID2;ZNF680;KDM2B;DYRK1A;PIAS2;FOXP2;FOXP1;ZNF90;HIC2;TBL1XR1;KAT6A;ZNF436;PHIP;ZNF676;HOMEZ;DDX5;INO80D;KMT2A;FOXO3;FOXO1;HMBOX1;ATXN1;ATXN7;NACC2;ZKSCAN8;ZKSCAN5;ZKSCAN4;ZNF662;ZBTB18;ZNF540;SMAD2;SMAD1;CBX5;POU2F1;ATP8B1;PHF12;NFXL1;ESRRG;SMAD5;NR4A1;GDNF;SP4;SP3;SP5;ZNF777;MDM4;BRWD1;OGT;BRWD3;ZNF652;ZNF772;NFE2L2;KDM5A;KDM5B;ELAVL2;HHEX;ZIC2;SIX4;SUMO1;TRPS1;ZNF649;ZIC1;ZNF644;LRRFIP1;MYBL1;ZNF521;NRG1;ETV1;ARID1A;ARID1B;TGFBR1;KLF17;CREB1;SETBP1;ASPH;TRAF6;ZNF516;LCORL;SOS1;L3MBTL4;HDAC4;SMARCD1;HDAC2;ZNF197;HLF;PLAG1;TAF9;ZBTB42;LIN9;ZBTB41;ZBTB40;ERBB4;SAP30L;ZNF506;MXI1;ZNF624;PCBD2;E2F5;ZNF500;E2F7;POLR2K;ZIC5;SOD2;ACVR2B;CTCFL;KLF7;ZNF737;BRMS1L
Negative Regulation of DNA-templated Transcription (GO:0045892)	1,20E+07	2,42E+10	RB1;PRDM6;WWC1;UBE2D3;ZBTB26;WWC2;EHMT1;ZBTB20;ARID4B;PRDM1;AHR;RBPJ;NR3C1;TNF;AMOT;ELK4;SALL1;MAEL;EPC1;EP300;KMT5A;MEF2A;KDM2B;SFMBT1;SOX13;RFX3;SOX11;DICER1;ISL1;DKK1;FOXP2;FOXP1;EREG;RUNX1;HIC2;THAP5;TBL1XR1;KAT6A;BCORL1;TP53;L3MBTL3;DDX5;SET;TSHZ3;ZBTB1;GATA3;FOXO3;FOXO1;ZBTB2;HMBOX1;ATXN1;ZNF706;NACC2;MIER1;ZNF703;INPP5K;ZNF148;SNX6;ZBTB18;ZNF540;SMAD2;JUN;XBP1;CBX5;POU2F1;ATP8B1;BCL11A;PHF12;NR1D2;PHC3;DLG1;GDNF;TNFSF4;SP3;SNAI2;MDM4;ZNF777;OGT;ZNF136;ZNF253;THRB;BHLHE41;PHF6;MECP2;DMBX1;HHEX;SUMO1;ZIC2;SIX4;TRPS1;LRRFIP1;NCK1;TSC22D4;PARP1;TET1;NRG1;PAX6;DUSP28;SIRT1;ZHX1;PAX2;KAT2B;CREB1;ZEB1;TRAF6;PAX9;TFEC;RARB;PLCB1;L3MBTL4;YAP1;HDAC4;HDAC2;HDAC8;DLL4;PURA;NSD1;DNAJB4;SAP30L;MXI1;FLNA;ZNF224;E2F7;RUNX1T1;SNCA;USP9X;DAB2IP;ACVR2B;KLF7;WT1;NFIB;BRMS1L;TRIM37;ZNF354B
Regulation of Transcription by RNA Polymerase II (GO:0006357)	5,19E+06	7,00E+10	RB1;EHMT1;IKZF2;RBPJ;ABRA;ELK4;EPC1;SOX6;SOX5;MEF2A;PRKCB;ZFP3;MRTFB;SOX13;EBF1;ZNF10;RFX3;SOX11;DICER1;ISL1;DKK1;RUNX1;THAP3;THAP5;RFX4;ZNF711;HOXB2;BCORL1;TP53;HOXB6;HOXB5;ZNF275;TSHZ3;ZNF391;TSHZ1;ZBTB1;PRDM15;GATA3;PIK3R1;ZBTB2;RAD21;MIER1;ZNF148;PPARGC1A;HOXC8;ZBTB7C;BARD1;EGR1;JUN;XBP1;JAG1;BCL11A;SS18L1;NFATC2;NR1D2;ZZZ3;FUBP3;AGO1;HNRNPD;SNAI2;CDK13;ZNF136;ZNF135;CC2D1B;ZNF493;BHLHE41;MIA3;TCF20;TFCP2L1;EFCAB7;PHF6;MECP2;DMBX1;MED14;ELOC;ZNF367;SS18;TSC22D4;PARP1;ZNF480;TET2;TET1;PAX6;PAX5;ZHX1;SIRT1;PAX2;PHF20L1;KAT2B;ZEB1;AEBP2;MED20;PAX9;TFEC;RARB;NCOA7;ZNF236;VGLL1;RHOQ;YAP1;PURA;NSD1;ZNF229;MEF2D;ZNF224;USP9X;DAB2IP;ELP4;ZNF33B;ZNF33A;NFIA;WT1;NFIB;ZNF333;ZNF354C;NEUROG2;PF4;ZNF354B;FOXA1;CCNT2;PRDM6;WWC1;ZBTB26;UBE2D3;WWC2;ZBTB20;RORA;ARID4B;PRDM1;AHR;NR3C1;TNF;HOXA13;AMOT;SALL1;SALL4;ZNF208;EP300;KMT5A;ARID2;KDM2B;LMO2;GTF2F1;PIAS2;FOXP2;FOXP1;HIC2;ADCYAP1;TBL1XR1;KAT6A;ZNF436;PHIP;HOMEZ;NOTCH2;DDX5;KMT2A;FOXO3;FOXO1;HMBOX1;ATXN1;NEUROD4;ATXN7;NACC2;ZKSCAN8;ZKSCAN5;ZKSCAN4;ZNF662;ZBTB18;ZNF540;SMAD2;SMAD1;AKIRIN1;CBX5;POU2F1;PHF12;ESRRG;SMAD5;NR4A1;DLG1;GDNF;SP4;SP3;SP5;ZNF777;MDM4;BRWD1;OGT;BRWD3;ZNF652;ZNF772;NFE2L2;THRB;YWHAB;PSIP1;HHEX;ZIC2;SIX4;ZNF407;TRPS1;ZNF649;ZIC1;SPX;ZNF644;LRRFIP1;MYBL1;ZNF521;NCK1;CHUK;ETV1;DUSP28;ARID1A;ARID1B;KLF17;CREB1;TRAF6;LCORL;SOS1;HDAC4;SMARCD1;NFAT5;HDAC2;ZNF197;HLF;PLAG1;TAF9;ZBTB42;LIN9;HDAC8;ZBTB41;ZBTB40;DLL4;DNAJB4;SAP30L;MXI1;ZNF624;E2F5;ZNF500;E2F7;SNCA;MACC1;ZIC5;SOD2;ACVR2B;ACVR2A;CTCFL;KLF7;ASXL3;BRMS1L;TRIM37;PAXBP1;SSBP3
Negative Regulation of Multicellular Organismal Process (GO:0051241)	6,78E+07	6,86E+10	RB1;FOXA1;GSK3B;HDAC2;PPP1R11;WWC1;HPN;WWC2;DOCK7;STC1;ARID4B;GATA3;REG3G;RBPJ;TNF;ROBO1;ADAMTS5;RPS6KA6;SPRED1;DUSP10;ZNF706;MDK;CASP3;SAP30L;NPTN;SPX;SMAD2;PARP1;SFMBT1;DAB2IP;TET1;ATP2B1;DKK1;ACVR2B;VASN;PROCR;BMP3;KLF7;NFIB;NOVA1;BRMS1L;IL17D;SRSF6;OGT;PFN2
Negative Regulation of RNA Biosynthetic Process (GO:1902679)	9,58E+07	7,75E+10	RB1;ZNF253;BHLHE41;EHMT1;AHR;RBPJ;NR3C1;TNF;MECP2;DMBX1;HHEX;SALL1;SUMO1;SIX4;ZIC2;MAEL;EPC1;KMT5A;TSC22D4;PARP1;SFMBT1;SOX13;RFX3;NRG1;SIRT1;ZHX1;PAX2;FOXP2;FOXP1;EREG;HIC2;ZEB1;KAT6A;TRAF6;PAX9;PLCB1;L3MBTL4;TP53;HDAC2;L3MBTL3;SET;TSHZ3;GATA3;FOXO1;HMBOX1;ATXN1;ZNF706;NACC2;ZNF703;INPP5K;ZNF148;ZNF224;RUNX1T1;SNX6;ZBTB18;ZNF540;SMAD2;JUN;CBX5;POU2F1;ATP8B1;PHF12;DAB2IP;NR1D2;PHC3;WT1;TNFSF4;BRMS1L;SP3;MDM4;ZNF777
Negative Regulation of Transcription by RNA Polymerase II (GO:0000122)	1,73E+08	1,17E+12	RB1;ZNF451;PRDM6;WWC1;UBE2D3;ZBTB26;WWC2;EHMT1;ZBTB20;ARID4B;PRDM1;RBPJ;NR3C1;TNF;AMOT;ELK4;SALL1;EPC1;EP300;KMT5A;MEF2A;KDM2B;SOX13;SOX11;DICER1;DKK1;ISL1;RUNX1;HIC2;THAP5;TBL1XR1;BCORL1;TP53;DDX5;ZBTB1;GATA3;FOXO3;ZBTB2;HMBOX1;ATXN1;NACC2;MIER1;ZNF148;ZBTB18;JUN;XBP1;CBX5;BCL11A;PHF12;NR1D2;DLG1;GDNF;SNAI2;MDM4;ZNF136;OGT;THRB;BHLHE41;PHF6;MECP2;HHEX;TRPS1;LRRFIP1;NCK1;TSC22D4;PARP1;TET1;PAX6;DUSP28;SIRT1;ZHX1;KAT2B;ZEB1;TRAF6;TFEC;RARB;HDAC4;YAP1;HDAC2;HDAC8;DLL4;PURA;NSD1;DNAJB4;SAP30L;MXI1;ZNF224;E2F7;SNCA;USP9X;DAB2IP;ACVR2B;KLF7;NFIB;WT1;BRMS1L;TRIM37;ZNF354B
Regulation of Gene Expression (GO:0010468)	1,07E+10	6,16E+11	RB1;CNTF;CSF1;PRDM6;WWC1;ZBTB26;WWC2;EHMT1;ZBTB20;RORA;AHR;NR3C1;TNF;SLC8A1;POFUT1;GPBP1;CCN4;SLC39A8;SOX6;ZNF680;FBXW7;SOX13;RC3H1;RFX3;SOX11;DICER1;ANK3;ATP1B1;DKK1;FOXP1;OLFM1;SFRP4;ZNF90;HIC2;KAT6A;ZNF711;ZNF676;TP53;NOTCH2;L3MBTL3;IGSF1;HPN;ZBTB1;ILDR2;ATXN7L3B;GATA3;NLK;ZBTB2;ATXN7;MIER1;ZNF703;UBR5;ZKSCAN5;ZNF148;PPARGC1A;SLC38A2;WNT3;CCR1;SMAD2;EGR1;MSR1;NFXL1;NFATC2;BRAF;NR1D2;ESRRG;LARP4;PUM1;SMAD5;OCLN;ZZZ3;FUBP3;GDNF;HNRNPF;SP3;HNRNPD;SNAI2;ZNF257;MYD88;NFE2L2;KDM5A;ZNF253;GSK3B;SETD5;KDM5B;PTPN22;SPOUT1;ELAVL2;ROBO1;MECP2;RO60;ITGB8;FLOT2;JARID2;PAX6;F3;SIRT1;PTBP2;TGFBR1;RNF168;TGFBR3;ACTA1;KAT2B;RBL1;SETBP1;HNRNPH1;MTF2;ZNF516;RBM20;ZNF117;VGLL1;YAP1;HDAC4;PLAG1;TAF9;TOB1;DLL4;PPP2CB;NWD1;NSD1;ZNF506;SAP30L;MBNL3;NTRK2;MOG;SORT1;ACVR2B;CTCFL;ZNF33A;NFIA;WT1;ZNF737;ZNF695;PF4
Regulation of Neuron Apoptotic Process (GO:0043523)	8,01E+09	0.0036966	GSK3B;CNTF;SET;KIF14;NDNF;FOXO3;TNF;CASP9;CASP7;SIX4;MDK;CASP3;TRIM2;PPARGC1A;SNX6;EGR1;NTRK2;KDM2B;KCNB1;SOD2;DKK1;ISL1;GDNF;NF1;IL6ST;CPEB4
Positive Regulation of Transcription by RNA Polymerase II (GO:0045944)	8,23E+09	0.0036966	FOXA1;CCNT2;RORA;AHR;RBPJ;NR3C1;TNF;ABRA;ELK4;SALL1;PWWP2A;EPC1;EP300;MEF2A;LMO2;MRTFB;RFX3;MED9;SOX11;GTF2F1;ISL1;RUNX1;ADCYAP1;THAP3;TBL1XR1;RFX4;ZNF711;HOXB2;PHIP;TP53;NOTCH2;KMT2A;PRDM15;GATA3;PIK3R1;FOXO3;FOXO1;NEUROD4;PPARGC1A;ZBTB18;SMAD2;EGR1;SMAD1;JUN;XBP1;AKIRIN1;JAG1;POU2F1;BCL11A;SS18L1;NFATC2;NR1D2;ESRRG;SMAD5;NR4A1;FUBP3;GDNF;AGO1;SP3;HNRNPD;CDK13;OGT;NFE2L2;THRB;YWHAB;PSIP1;MIA3;TCF20;EFCAB7;MED11;MED14;HHEX;ZNF407;SPX;MYBL1;NCK1;SS18;CHUK;PARP1;TET2;TET1;ETV1;PAX6;SIRT1;PAX2;KAT2B;CREB1;TRAF6;MED20;TFEC;RARB;NCOA7;SOS1;RHOQ;YAP1;HDAC4;NFAT5;HDAC2;HLF;PLAG1;TAF9;MEF2D;E2F7;MACC1;DAB2IP;ACVR2A;CTCFL;KLF7;NFIA;WT1;NFIB;ASXL3;PAXBP1;SSBP3;NEUROG2;PF4

Intracellular Glucose Homeostasis (GO:0001678)	1,02E+11	0.0041150	XBP1;KCNB1;ZBTB20;PRKCA;PIK3R1;HOOK3;SIRT1;FOXO1;PAX2;IGF1R;LRRC8D;ZNF236;PPARGC1A;OGT
Glutamate Receptor Signaling Pathway (GO:0007215)	1,21E+11	0.0044626	GRM3;GRIA2;GRIN2A;GRM5;GRM7;KCNB1;GNAQ;GRM8;GRIK1;PLCB1;GRIA3;CPEB4
Regulation of Cell Migration (GO:0030334)	1,33E+11	0.0044953	GSK3B;MCTP1;CSF1;KIF14;RORA;MIA3;ARID4B;ROBO1;AMOT;IGF1R;FGF7;SLK;DUSP10;MDK;DPYSL3;PLXNC1;ARID2;SRGAP2B;APPL1;LMO4;SEMA6D;TET1;PRKCA;F3;TGFBR1;RHOB;TGFBR3;SPATA13;NUMB;ADAM9;SGK3;SOS1;CBLL1;HBEGF;FBN2;LRRC15;HDAC2;SYNPO2;CAMK2A;SEMA3G;STC1;FOXO3;RTN4;ERBB4;CCL5;SAP30L;ZNF703;GNA12;FLNA;RICTOR;SLIT2;RFFL;SRGAP1;CCR1;XBP1;JAG1;LAMB1;SEMA4G;SOD2;BRMS1L;TRIP6;NF1;SNAI2;OGT;RECK
Regulation of Hepatocyte Proliferation (GO:2000345)	1,74E+11	0.0054016	XBP1;FBXW7;MDK;HPN;RTN4
Proteasome-Mediated Ubiquitin-Dependent Protein Catabolic Process (GO:0043161)	3,22E+10	0.0087861	GSK3B;UBXN1;UBE2D3;KIF14;LTN1;TRIM2;FBXO4;FBXO6;FBXO9;KCMF1;FBXW7;ARMC8;SIRT1;PSMA1;PSMA2;TBL1XR1;PELI1;PSMD12;RNF34;RHOBTB3;KLHL11;UBR1;DTX4;ANAPC10;FBXL20;PRAMEF13;PRAMEF2;PRAMEF1;PRAMEF14;UBR5;FEM1C;ASCC3;RFFL;DCAF12;DCAF13;CRBN;CSNK1A1;FEM1B;SIAH2;SIAH1;UBE2A;KBTBD3;RNF145;UBE2W;ITCH;KBTBD6;KLHL7;TRIP12;NFE2L2
Regulation of Synaptic Transmission, Glutamatergic (GO:0051966)	3,62E+11	0.0087861	UNC13A;TSHZ3;PLPPR4;GRIK1;ATP1A2;TNF;GRM3;FXR1;GRM5;GRIN2A;GRM7;GRM8;CACNG2;SLC38A2
Negative Regulation of Apoptotic Process (GO:0043066)	3,64E+09	0.0087861	RB1;FANK1;GSK3B;CNTF;KIF14;TNF;ICAM1;IGF1R;SIX4;MDK;KDM2B;FGG;PRKCA;DKK1;ISL1;SIRT1;PAX2;DNAJC3;CREB1;CEACAM5;PPIF;PHIP;IL6ST;SOS1;FXN;TP53;NOTCH2;YAP1;HDAC2;SET;MAPKAP1;HPN;TAF9;NDNF;GATA3;LRP2;PIK3R1;GLYAT;FOXO1;HSP90B1;PKHD1;BAG3;ERBB4;FLNA;RICTOR;PPARGC1A;SNX6;SNCA;BARD1;NTRK2;XBP1;SIAH2;BRAF;SOD2;SMAD5;HIPK3;ITCH;GDNF;WT1;IL7;SNAI2;CPEB4;NFE2L2
Regulation of JUN Kinase Activity (GO:0043506)	3,85E+10	0.0087861	PTPN1;TAOK3;TRAF6;DAB2IP;PTPN22;MAP3K13;TANK;TNF;MAP3K7;MAP3K4;HIPK3
Positive Regulation of Cell Differentiation (GO:0045597)	4,10E+09	0.0087861	YAP1;FBN2;GSK3B;SMARCD1;CSF1;TNF;NEUROD4;MDK;ZNF703;CCN4;CCN2;SOX6;ARID2;ECT2;WNT3;SOX5;FZD1;SMAD2;MSR1;XBP1;AKIRIN1;SOX11;LAMB1;TMEM64;TENT5A;ARID1A;ISL1;GDF5;SMAD5;ARID1B;ACVR2B;PAX2;TGFBR1;ACVR2A;OLFM1;KITLG;ZEB1;CREB1;GDNF;VWC2;PLCB1;IL6ST;SOS1;PF4
Proteasomal Protein Catabolic Process (GO:0010498)	4,31E+10	0.0087861	GSK3B;UBXN1;FAF2;UBE2D3;KIF14;TRIM2;FBXO6;MAN1A1;FBXO9;KCMF1;FBXW7;ARMC8;SIRT1;PSMA1;PSMA2;TBL1XR1;PELI1;FAM8A1;PSMD12;RNF34;RHOBTB3;KLHL11;UBR1;DTX4;HSP90B1;PRAMEF13;PRAMEF2;PRAMEF1;PRAMEF14;UBR5;FEM1C;RFFL;DCAF13;XBP1;TOR1A;CRBN;CSNK1A1;FEM1B;SIAH2;AMFR;SIAH1;UBE2A;KBTBD3;RNF145;UBE2W;ITCH;KBTBD6;KLHL7;DNAJC10;TRIP12;NFE2L2;CUL4B
Positive Regulation of Epithelial Cell Proliferation (GO:0050679)	4,56E+10	0.0087861	NOTCH2;YAP1;XBP1;HPN;PRKCA;SOX11;MIA3;LAMB1;REG3G;TNF;F3;SIRT1;RTN4;PAX2;TGFBR1;PKHD1;NR4A1;FGF7;EPGN;MDK;ERBB4;ZNF703;SRSF6
Positive Regulation of miRNA Metabolic Process (GO:2000630)	4,56E+10	0.0087861	FOXA1;NOTCH2;EGR1;JUN;POU2F1;MRTFB;PAX6;GATA3;NR3C1;FOXO3;TNF;LIN28B;WT1;TP53
Positive Regulation of miRNA Transcription (GO:1902895)	5,02E+11	0.0088306	FOXA1;NOTCH2;EGR1;JUN;POU2F1;MRTFB;PAX6;GATA3;FOXO3;NR3C1;TNF;WT1;TP53
Regulation of Neuron Projection Development (GO:0010975)	5,17E+10	0.0088306	GSK3B;HDAC2;MYCBP2;TWF1;NDNF;CAMSAP2;ARHGAP44;FUT9;MDK;DPYSL3;NPTN;EP300;ABL2;DENND5A;ALKAL1;PDLIM5;LZTS3;PPFIA2;ZNF365;MINAR1;FZD1;NTRK2;BCL11A;DCC;KIDINS220;DAB2IP;MBOAT1;ZDHHC15;CCDC88A;RTN4RL1
Positive Regulation of JUN Kinase Activity (GO:0043507)	5,24E+10	0.0088306	PTPN1;TAOK3;TRAF6;DAB2IP;MAP3K13;TANK;TNF;MAP3K7;MAP3K4
- Reg of Transmembrane Receptor Prot Serine/Threonine Kinase Sgnlng Pway (GO:0090101)	7,83E+10	0.0126649	ZNF451;XBP1;HDAC2;IGSF1;UBE2D3;CRIM1;TET1;ARID4B;LRP2;LEMD3;DKK1;SIRT1;VASN;TGFBR3;SPRED1;SAP30L;BRMS1L;RBPMS2;VWC2;SRFBP1;OGT;GPR155;SNX6
Positive Regulation of DNA-templated Transcription (GO:0045893)	8,47E+10	0.0130581	FOXA1;CCNT2;ICE2;CD80;RORA;AHR;RBPJ;NR3C1;TNF;ABRA;ELK4;SALL1;EPC1;EP300;MEF2A;LMO2;MRTFB;DYRK1A;RFX3;SOX11;GTF2F1;ISL1;RUNX1;ADCYAP1;THAP3;TBL1XR1;RFX4;KAT6A;ZNF711;HOXB2;PHIP;TP53;NOTCH2;INO80D;KMT2A;PRDM15;GATA3;PIK3R1;FOXO3;FOXO1;SEC14L2;NEUROD4;ATXN7;INPP5K;PPARGC1A;ZBTB18;FZD1;SMAD2;EGR1;SMAD1;JUN;XBP1;AKIRIN1;JAG1;POU2F1;BCL11A;SS18L1;NFATC2;NR1D2;ESRRG;SMAD5;NR4A1;FUBP3;GDNF;AGO1;SP3;HNRNPD;CDK13;OGT;NFE2L2;FANK1;KDM5A;THRB;YWHAB;PSIP1;MIA3;TCF20;EFCAB7;WDR43;MED14;HHEX;ZIC2;SIX4;ZNF407;ZIC1;SPX;MYBL1;NCK1;SS18;CHUK;PARP1;TET2;TET1;ETV1;PAX6;ARID1A;SIRT1;ARID1B;PAX2;TGFBR1;KAT2B;CREB1;ASPH;TRAF6;ZNF516;TFEC;RARB;NCOA7;PLCB1;SOS1;RHOQ;YAP1;HDAC4;NFAT5;HDAC2;HLF;PLAG1;TAF9;ERBB4;NSD1;PCBD2;MEF2D;E2F7;MACC1;DAB2IP;GTF2H1;ACVR2A;CTCFL;KLF7;NFIA;WT1;NFIB;ASXL3;CAMK4;PAXBP1;SSBP3;NEUROG2;PF4
Negative Regulation of Cell Motility (GO:2000146)	8,72E+10	0.0130581	GSK3B;HDAC2;MCTP1;STC1;ARID4B;MIA3;GATA3;FOXO3;ROBO1;DUSP10;SAP30L;DPYSL3;SLIT2;ARID2;SRGAP1;SRGAP2B;JAG1;TET1;TGFBR1;RHOB;TGFBR3;BRMS1L;NF1;OGT;RECK
Positive Regulation of Developmental Process (GO:0051094)	1,09E+12	0.0157071	GSK3B;SMARCD1;HDAC2;ARID4B;TNF;RTN4;NEUROD4;MDK;SAP30L;ZNF703;CCN4;C21ORF91;CCN2;SOX6;ARID2;SOX5;SS18;SMAD1;XBP1;RBM19;DAB2IP;TET1;DDHD1;ARID1A;ISL1;SIRT1;ARID1B;PAX2;TGFBR1;ZDHHC15;GDNF;WT1;BRMS1L;PLCB1;MAP3K13;ODAPH;OGT
miRNA Processing (GO:0035196)	1,20E+12	0.0167983	SMAD2;SMAD1;DDX5;PUS10;LIN28B;TUT4;AGO1;DICER1;PUM1;SPOUT1
Regulation of Leukocyte Adhesion to Vascular Endothelial Cell (GO:1904994)	1,49E+12	0.0200487	NFAT5;IRAK1;FUT9;MDK;TRAF6;TNF
Regulation of miRNA Transcription (GO:1902893)	1,60E+12	0.0208413	FOXA1;NOTCH2;EGR1;DDX5;JUN;POU2F1;MRTFB;PAX6;GATA3;NR3C1;FOXO3;TNF;NFIB;WT1;TP53
Intracellular Signaling Cassette (GO:0141124)	1,75E+11	0.0221301	RYR2;MCTP1;NDNF;GATA3;PIK3R1;LRP2;TANK;RERG;IGF1R;TRIM8;IRAK1;CXCR2;MAP3K20;EP300;MAPK1;MAP3K8;MAP3K7;MAP3K4;MEF2A;CCR1;P2RY12;SMAD2;MAP3K3;GUCY1A2;SMAD1;JUN;XBP1;GUCY1A1;CHUK;SIAH2;AMFR;PRKCA;BRAF;DUSP9;SMAD5;PIAS2;CCDC88A;ARHGAP32;CREB1;SLC7A8;TAOK3;GDNF;TRAF6;NF1;PDE7B;MCTP2;PDE7A;NFE2L2
Negative Regulation of Striated Muscle Cell Differentiation (GO:0051154)	1,84E+12	0.0225962	HDAC4;XBP1;CEACAM5;BHLHE41;FRS2;SOX6;DKK1
Protein Dephosphorylation (GO:0006470)	2,11E+12	0.0248015	PTPN1;DAPP1;PTPN22;DUSP28;PTPN11;PTPN12;SSH2;DUSP9;PPM1E;PTPN14;MTM1;PPM1G;PPP2CB;PTPRB;PTPN4;ZNF367;TNS2;PTPN2
Ubiquitin-Dependent Protein Catabolic Process (GO:0006511)	2,15E+12	0.0248015	GSK3B;UBXN1;PPP1R11;UBE3C;UBE2D3;KIF14;HERC3;TRIM2;FBXO4;ELOC;FBXO8;FBXO9;KCMF1;FBXW7;RC3H1;ARMC8;SIRT1;RNF168;PSMA1;PSMA2;TBL1XR1;PELI1;CHMP5;PSMD12;RNF34;TSG101;RHOBTB3;KLHL11;UBR1;DTX4;PRAMEF2;PRAMEF13;PRAMEF1;UBR5;PRAMEF14;FEM1C;RFFL;DCAF13;XBP1;ZRANB1;CRBN;CSNK1A1;USP9X;FEM1B;AMFR;SIAH2;SIAH1;UBE2A;CNOT4;KBTBD3;RNF145;UBE2W;ITCH;KBTBD6;KLHL7;TRIP12;RNF103-CHMP3;NFE2L2
Axon Development (GO:0061564)	2,52E+12	0.0277295	DCC;APLP2;PLPPR4;DOCK7;MTR;ANK3;NREP;NMNAT2;ISL1;RTN4;PAX2;CAMSAP2;KLF7;NEUROD4;GRM7;RTN4RL1;SLITRK4;GPM6B;METAP1;NEUROG2
Regulation of Protein Import Into Nucleus (GO:0042306)	2,54E+11	0.0277295	XBP1;SUMO1;YWHAB;BAG3;UBR5;ZIC1;EP300;FLNA;PIK3R1;EFCAB7;ECT2
Positive Regulation of Osteoblast Differentiation (GO:0045669)	2,77E+11	0.0291447	FBN2;YAP1;FZD1;CCN4;SOX11;TENT5A;IL6ST;SOS1;ACVR2B;SMAD5;WNT3;ACVR2A
Regulation of Osteoblast Differentiation (GO:0045667)	2,81E+12	0.0291447	FBN2;YAP1;FZD1;GSK3B;DDX5;CRIM1;PRKCA;SOX11;TENT5A;TMEM64;ACVR2B;SMAD5;ACVR2A;CCN4;SNAI2;IL6ST;SOS1;WNT3
Modulation of Chemical Synaptic Transmission (GO:0050804)	3,19E+12	0.0322846	GRIA2;GSK3B;SLC38A1;UNC13A;MCTP1;PLPPR4;CAMK2A;CASK;CACNA1A;GRIK1;ATP1A2;TNF;FXR1;GRM3;GRM5;GRM7;GRM8;DLGAP1;MCTP2;KCNK2;GRIA3
Negative Regulation of Gene Expression (GO:0010629)	3,37E+12	0.0328347	RB1;YAP1;NOTCH2;HDAC4;GSK3B;PPP1R11;EIF4A3;PTPN22;TNF;TOB1;ROBO1;FXR1;MECP2;DLL4;SYNCRIP;PPP2CB;CASP3;MAEL;NPTN;EPC1;FLOT2;ITGB8;ZNF148;PAIP2;NUDT12;CCR1;ZNF540;MSR1;FBXW7;IFRD2;RPL13A;SOX11;DICER1;ATP2B1;MEX3D;PUM1;SIRT1;SMAD5;PTBP2;FOXP1;KAT2B;OLFM1;OCLN;RBL1;WT1;CAPRIN1;MTF2;TRIM37
Epidermal Growth Factor Receptor Signaling Pathway (GO:0007173)	3,41E+12	0.0328347	BTC;EPGN;ERBB4;REPS2;ABL2;MAPK1;BRAF;PTPN11;GRB2;SOS1;EREG;HBEGF

Positive Regulation of RNA Biosynthetic Process (GO:1902680)	3,53E+11	0.0331612	FANK1;KDM5A;CD80;RORA;AHR;TNF;MED14;HHEX;SALL1;SIX4;ZIC2;ZIC1;EPC1;EP300;MYBL1;DYRK1A;RFX3;PAX6;ARID1A;ARID1B;PAX2;TGFBR1;RUNX1;KAT2B;CREB1;ASPH;TBL1XR1;KAT6A;ZNF516;PHIP;ZNF711;PLCB1;TP53;HDAC4;YAP1;HDAC2;INO80D;KMT2A;TAF9;GATA3;FOXO3;FOXO1;SEC14L2;ATXN7;ERBB4;NSD1;INPP5K;PCBD2;PPARGC1A;FZD1;SMAD2;EGR1;JUN;SS18L1;NFATC2;NR1D2;GTF2H1;SMAD5;CTCFL;FUBP3;NFIB;WT1;CAMK4;SP3;HNRNPD;OGT;NFE2L2
Negative Regulation of MAPK Cascade (GO:0043409)	3,90E+12	0.0358656	PTPN1;XBP1;SEMA6A;SPRY4;PRDM15;DAB2IP;PTPN22;DUSP28;DUSP9;FOXO1;IGF1R;RPS6KA6;DLG1;SPRED1;ITCH;DUSP10;TAOK3;NF1;INPP5K;SH2B3;PTPN2;FKTN
Negative Regulation of Stem Cell Population Maintenance (GO:1902455)	4,15E+11	0.0366376	HDAC2;ZNF706;BRMS1L;SAP30L;TET1;ARID4B;OGT
Negative Regulation of ERK1 and ERK2 Cascade (GO:0070373)	4,17E+11	0.0366376	PTPN1;RPS6KA6;SPRED1;XBP1;DLG1;DUSP10;SEMA6A;SPRY4;DAB2IP;DUSP28;DUSP9;PTPN2
Cellular Response to Glucose Stimulus (GO:0071333)	4,52E+11	0.0388500	XBP1;KCNB1;ZBTB20;PRKCA;SELENOT;RAB11FIP2;ZNF236;OGT;PAX2;IGF1R
Regulation of TOR Signaling (GO:0032006)	4,65E+11	0.0391735	MINAR1;GSK3B;XBP1;UBR1;F3;SIRT1;C9ORF72;PREX2;PKHD1;CCL5;DDIT4;GNA12;RICTOR;RFFL;LAMTOR3;FBXO9
ERBB Signaling Pathway (GO:0038127)	4,96E+11	0.0398428	NRG1;BRAF;PTPN11;EREG;BTC;EPGN;ERBB4;REPS2;ABL2;MAPK1;GRB2;SOS1;HBEGF
Transcription Initiation-Coupled Chromatin Remodeling (GO:0045815)	5,06E+10	0.0398428	KAT2B;SMARCD1;EGR1;KMT2A;GDNF;TET2;EP300;TET1;ARID1A;ARID1B;TP53;CTCFL
Negative Regulation of Cellular Response to Growth Factor Stimulus (GO:0090288)	5,12E+11	0.0398428	PTPN1;UBE2D3;SPRY4;CRIM1;DAB2IP;CASK;LRP2;LEMD3;HHEX;MMRN1;RBPMS2;VWC2;AGTR2;SLIT2;GPR155
Negative Regulation of Epithelial Cell Proliferation (GO:0050680)	5,12E+11	0.0398428	FBXW7;HPN;DAB2IP;CASK;GATA3;GDF5;MTSS1;ROBO1;EREG;ATP5IF1;TGFBR3;DUSP10;NFIB;NF1;SNAI2
Regulation of Cell Differentiation (GO:0045595)	5,69E+11	0.0434401	GSK3B;SMARCD1;DDX5;HDAC2;CCNT2;PLEKHB2;CSF1;GATA3;NREP;TNF;NEUROD4;MDK;TRPS1;CCN4;FLOT2;CCN2;ARID2;SMAD2;SS18;PRKCA;NR1D2;ARID1A;ISL1;ARID1B;TGFBR1;RUNX1;GDNF;NF1;SNAI2;PDCD2
Cellular Response to Transforming Growth Factor Beta Stimulus (GO:0071560)	6,26E+11	0.0460428	SMAD2;SMAD1;JUN;USP9X;USP9Y;NR3C1;NLK;GDF5;SMAD5;SIRT1;PIAS2;TGFBR1;TGFBR3;ADAM9;SOX6;MAP3K7;SOX5;APPL1
Negative Regulation of Cell Migration (GO:0030336)	6,26E+11	0.0460428	GSK3B;HDAC2;MCTP1;NEXMIF;STC1;ARID4B;MIA3;FOXO3;ROBO1;DLL4;DUSP10;SAP30L;DPYSL3;SLIT2;ARID2;SRGAP1;SRGAP2B;JAG1;DAB2IP;TET1;TGFBR1;RHOB;TGFBR3;BRMS1L;NF1;OGT;RECK;PFN2
Regulation of Chondrocyte Differentiation (GO:0032330)	6,51E+11	0.0469875	MDK;TRPS1;SNAI2;PTPN11;SOX6;GDF5;TGFBR1;SOX5
Positive Regulation of Gene Expression (GO:0010628)	6,98E+11	0.0494933	GSK3B;CNTF;CSF1;EIF4A3;ELAVL4;PTPN22;TNF;ELAVL1;LARP1B;ROBO1;ITGB8;DYRK1A;SOX11;PAX6;ANK3;DKK1;F3;TGFBR1;EREG;TGFBR3;OLFM1;SFRP4;ACTA1;CSDE1;RBM20;TP53;YAP1;HPN;STK39;PIK3R1;FXR1;DLL4;NWD1;UBR5;PPARGC1A;SLC38A2;WNT3;NTRK2;EGR1;CADM1;BRAF;LARP4;CTCFL;OCLN;FUBP3;GDNF;WT1;TNFSF4;HNRNPD;EIF3C;OGT;MYD88;PF4;NFE2L2
Positive Regulation of Lymphocyte Differentiation (GO:0045621)	7,12E+11	0.0496741	SMARCD1;XBP1;MDK;IL7;INPP5D;ZBTB1;GATA3;ARID2;ARID1A;ARID1B
Organelle Organization (GO:0006996)	7,36E+11	0.0504632	RB1;TDRKH;GSK3B;ABCD3;FAF2;MIA3;LETM2;PCLO;MDK;ATP7A;CXADR;SEMA6A;PDCD6IP;PARP1;VPS13C;RAB30;RAB31;PALLD;KAT6A;PHIP;BLZF1;CHMP5;ATL3;STX17;MAPKAP1;AFG1L;RTN4;FGD2;MTM1;ATXN7;NAA25;RICTOR;CCDC32;PPARGC1A;RAB6B;EPS15;PDZD8;PEX16;ZRANB1;CSNK1A1;ATP8B1;SORT1;SEC16A;LARP4;TJAP1;FMNL3;SEC23IP;ABI2;REEP5;VAPA;BRWD1;RNF103-CHMP3;ZNF135;BRWD3
Transforming Growth Factor Beta Receptor Signaling Pathway (GO:0007179)	8,00E+11	0.0534315	SMAD2;SMAD1;JUN;USP9X;USP9Y;NLK;SIRT1;GDF5;SMAD5;PIAS2;TGFBR1;TGFBR3;ADAM9;MAP3K7;APPL1
Negative Regulation of Developmental Growth (GO:0048640)	8,06E+11	0.0534315	DUSP10;BCL11A;DCC;WWC1;SEMA6D;WWC2;SEMA3G;DIP2B;RTN4
Pulmonary Valve Development (GO:0003177)	8,32E+11	0.0542447	NOTCH2;SMAD2;ADAMTS5;JAG1;SLIT2;RBPJ;ROBO1
Regulation of T Cell Differentiation (GO:0045580)	9,01E+11	0.0578114	SMARCD1;XBP1;MDK;IL7;CAMK4;ZBTB1;GATA3;PRDM1;ARID2;ARID1A;ARID1B
Regulation of Epithelial Cell Proliferation (GO:0050678)	9,26E+11	0.0584882	YAP1;HPN;DAB2IP;LAMB1;GDF5;MTSS1;PAX2;EREG;TGFBR3;PKHD1;NR4A1;FGF7;EPGN;DUSP10;SIX4;ERBB4;SRSF6
Positive Regulation of Endoplasmic Reticulum Unfolded Protein Response (GO:1900103)	9,53E+11	0.0593151	PTPN1;BCL2L11;TMEM33;DAB2IP;PIK3R1
Aortic Valve Development (GO:0003176)	0.0010864	0.0633999	RB1;DLL4;SMAD2;ADAMTS5;JAG1;SNAI2;GATA3;SLIT2;RBPJ;ROBO1
Regulation of RNA Biosynthetic Process (GO:2001141)	0.0010958	0.0633999	RB1;KDM5A;SETD5;ZNF253;KDM5B;WWC1;WWC2;RORA;AHR;NR3C1;ELAVL2;POFUT1;GPBP1;SLC39A8;SOX6;ZNF680;SOX13;RFX3;TGFBR1;KAT2B;ZNF90;SETBP1;KAT6A;ZNF516;ZNF117;ZNF676;TP53;VGLL1;PLAG1;IGSF1;TAF9;NLK;NSD1;ZNF506;SAP30L;MIER1;ZNF703;ZKSCAN5;PPARGC1A;SMAD2;NFATC2;NFXL1;NR1D2;ESRRG;ACVR2B;ZNF33A;ZZZ3;NFIA;GDNF;WT1;ZNF737;SP3;HNRNPD;SNAI2;ZNF257;ZNF695
Cellular Response to Oxygen-Containing Compound (GO:1901701)	0.0011030	0.0633999	GSK3B;CD80;CACNA1A;RORA;MTR;PTPN22;AHR;UBR1;PPM1E;SLC8A1;FOXO1;ICAM1;IGF1R;HSP90B1;GRIN2A;CASP7;GRM5;ZNF703;PDK3;INPP5K;EP300;ABL2;SLIT2;SLC38A2;PF4V1;WNT3;SNX6;P2RY12;EGR1;XBP1;CACNA2D1;DAB2IP;IL36G;TET1;PPBP;PEX13;PAX2;CACNB1;CREB1;WT1;TNFSF4;TRAF6;ADAM9;TP53;CCL28;MYD88;PF4;CHMP5
Cellular Response to Hexose Stimulus (GO:0071331)	0.0011225	0.0633999	XBP1;KCNB1;ZBTB20;PRKCA;ZNF236;OGT;PAX2;IGF1R
Interleukin-33-Mediated Signaling Pathway (GO:0038172)	0.0011287	0.0633999	IRAK1;TRAF6;MAP3K7;MYD88
Positive Regulation of IRE1-mediated Unfolded Protein Response (GO:1903896)	0.0011287	0.0633999	PTPN1;BCL2L11;TMEM33;DAB2IP
Regulation of Mammary Gland Epithelial Cell Proliferation (GO:0033599)	0.0011287	0.0633999	ZNF703;GATA3;RTN4;ROBO1
Regulation of Transforming Growth Factor Beta Receptor Signaling Pathway (GO:0017015)	0.0011571	0.0641023	SMAD2;ZNF451;XBP1;HDAC2;TET1;ARID4B;SOX11;NREP;LEMD3;SIRT1;VASN;TGFBR3;SPRED1;SAP30L;BRMS1L;ZNF703;EP300;SRFBP1;OGT;SNX6
Cell Surface Receptor Protein Tyrosine Kinase Signaling Pathway (GO:0007169)	0.0011770	0.0643223	GSK3B;CSF1;RORA;PIK3R1;CSF2RA;FOXO1;IGF1R;GHR;FGF7;ERBB4;CASP3;MAPK1;ALKAL1;ANKS1B;APPL1;NCK1;EPHA5;KL;NTRK2;XBP1;YES1;CHURC1;SORT1;FRS2;NRG1;PTPN11;SORBS1;SHCBP1;MTSS1;FGF14;GDNF;DDIT4;GRB2;PHIP;PLCB1;SOS1;ANGPTL1;RHOQ;PTPN2
Positive Regulation of Biosynthetic Process (GO:0009891)	0.0012086	0.0651718	MTPN;EGR1;HDAC2;ZBTB20;MIA3;TTF2;OPRM1;SORBS1;PTGS2;TNF;SIRT1;FOXO1;RUNX1;CREB1;PPARGC1A;PTPN2
Regulation of Stem Cell Population Maintenance (GO:2000036)	0.0012317	0.0655444	SS18;SMARCD1;HDAC2;ZNF706;SAP30L;BRMS1L;TET1;ARID4B;ARID1A;ELAVL1;OGT;WDR43
Regulation of Insulin Receptor Signaling Pathway (GO:0046626)	0.0013015	0.0678838	PTPN1;PRKCB;MAPKAP1;INPP5K;PTPN11;SORBS1;SIRT1;OGT;TNS2;PTPN2;NCK1
Positive Regulation of T Cell Differentiation (GO:0045582)	0.0013249	0.0678838	SMARCD1;XBP1;ZBTB1;GATA3;ARID1A;FOXO3;ARID1B;DUSP10;VNN1;MDK;IL7;TNFSF4;ARID2
Positive Regulation of Ossification (GO:0045778)	0.0013261	0.0678838	FBN2;KL;PTPN11;SOX11;ATP2B1;TENT5A;ACVR2B;SLC8A1;GPM6B;ACVR2A
Negative Regulation of Extrinsic Apoptotic Signaling Pathway (GO:2001237)	0.0013733	0.0694244	GSK3B;RNF34;SIAH2;FGG;EYA4;NRG1;TNF;TGFBR1;ICAM1;GDNF;SNAI2;PHIP;RFFL;PF4
Cellular Response to Cytokine Stimulus (GO:0071345)	0.0014020	0.0699997	GSK3B;NFAT5;USP32;CAMK2A;STK39;GATA3;PIK3R1;TANK;CSF2RA;EDA2R;GHR;SYNCRIP;RO60;CCL5;DPYSL3;INPP5K;SH2B3;CCR1;EGR1;XBP1;CHUK;DAB2IP;RC3H1;IL36G;RPL13A;PRKCA;PTPN11;F3;SIRT1;EPOR;EREG;LRCH1;IL7;TRAF6;IL6ST;SOS1;PF4;NFE2L2
Positive Regulation of Protein Import Into Nucleus (GO:0042307)	0.0014437	0.0712036	XBP1;UBR5;ZIC1;EP300;FLNA;PIK3R1;EFCAB7;ECT2
Transforming Growth Factor Beta Receptor Superfamily Signaling Pathway (GO:0141091)	0.0015182	0.0739726	SMAD2;SMAD1;DDX5;JUN;USP9X;USP9Y;INHBB;NLK;GDF5;SMAD5;SIRT1;ACVR2B;PIAS2;TGFBR1;ACVR2A;TGFBR3;ADAM9;MAP3K7;APPL1
Regulation of ERK1 and ERK2 Cascade (GO:0070372)	0.0015369	0.0739939	NOTCH2;PTPN22;TNF;ICAM1;PKHD1;RPS6KA6;SPRED1;DUSP10;ERBB4;TNFAIP8L3;CCN2;ALKAL1;CCR1;PTPN1;XBP1;SEMA6A;FBXW7;FGG;SPRY4;DAB2IP;PRKCA;BRAF;DUSP28;NRG1;PTPN11;OPRM1;DUSP9;TGFBR3;ADCYAP1;DLG1;PTPN2
Negative Regulation of Neuron Apoptotic Process (GO:0043524)	0.0015787	0.0745547	NTRK2;CNTF;SET;KDM2B;KIF14;NDNF;SOD2;ISL1;SIX4;GDNF;MDK;IL6ST;PPARGC1A;SNX6;CPEB4

Cellular Response to Amyloid-Beta (GO:1904646)	0.0015854	0.0745547	CACNB1;GSK3B;GRIN2A;GRM5;CACNA2D1;CACNA1A;ICAM1;IGF1R;SNX6
Positive Regulation of Cell Projection Organization (GO:0031346)	0.0016865	0.0783965	FZD1;NTRK2;BCL11A;KIDINS220;SS18L1;DAB2IP;TWF1;NDNF;ROBO1;FUT9;MDK;CAPRIN1;DPYSL3;NPTN;EP300;PLXNC1;ALKAL1;SLIT2;MAP3K13
Regulation of Intracellular Signal Transduction (GO:1902531)	0.0017087	0.0785243	PHLPP1;DYRK2;RNF34;SETD9;JPT2;WWC1;WWC2;PTPN22;PPM1K;SAMSN1;TNF;FGD2;PREX2;PKHD1;ARHGAP44;ARHGAP20;SPRED1;MPZ;GNA12;EP300;PIP5K1B;RICTOR;KMT5A;ARHGEF40;ECT2;RFFL;FBXO9;AMFR;DAB2IP;PTPN11;SIRT1;ARHGAP32;DLG1;TAOK3;KAT6A;TRAF6;SPATA13;GRB2;DIPK2A
Positive Regulation of Cellular Process (GO:0048522)	0.0017900	0.0804892	RYR2;GSK3B;CSF1;KIF14;TNF;IGF1R;GHR;FGF7;POFUT2;ESM1;MDK;CCN4;CCN2;ARID2;SS18;CNOT6L;PRKCA;DUSP28;NRG1;ACSL4;DICER1;ARID1A;ISL1;SIRT1;ARID1B;TGFBR1;EREG;BTC;CCKBR;PHIP;IL6ST;FXN;TP53;TSPYL5;HBEGF;ANKRD17;HDAC4;YAP1;MTPN;SMARCD1;HDAC2;MAPKAP1;BRAT1;HPN;TTK;REG3G;CSF2RA;CDKN2AIP;PKHD1;NEUROD4;ERBB4;CCL5;ZNF703;CXCR2;MAP3K20;RICTOR;S1PR3;NTRK2;XBP1;LAMB1;EPOR;EPGN;KITLG;GDNF;IL7;TNFSF4;DNAJA2;ACER3;EIF4G2;METAP1
Dephosphorylation (GO:0016311)	0.0017913	0.0804892	PTPN1;PLPPR4;DAPP1;PTPN22;DUSP28;PTPN12;SSH2;DUSP9;PTPN14;SGPP1;MTM1;PPM1G;PPP2CB;PTPRB;DUSP10;INPP5K;PTPN4;ZNF367
Positive Regulation of Neuron Projection Development (GO:0010976)	0.0018878	0.0838969	FZD1;SMAD1;NTRK2;BCL11A;KIDINS220;DAB2IP;TWF1;NDNF;ZDHHC15;PTPRD;FUT9;MDK;CAPRIN1;DPYSL3;NPTN;EP300;ALKAL1
Regulation of Ossification (GO:0030278)	0.0019345	0.0850340	SMAD2;CYP27B1;BMP3;MDK;PTPN11;SOX11;TENT5A;RBPJ;ACVR2B;DKK1
Response to Interleukin-1 (GO:0070555)	0.0019810	0.0857791	HDAC4;IRAK1;USP32;TRAF6;CCL5;TAF9;DAB2IP;RC3H1;PRKCA;TANK;MYD88;SNCA
Cellular Response to Insulin Stimulus (GO:0032869)	0.0019938	0.0857791	GSK3B;XBP1;PARP1;MAPKAP1;ZDHHC7;INHBB;PIK3R1;SORBS1;FOXO1;IGF1R;KAT2B;RAB31;INPP5K;PHIP;GRB2;RHOQ;PTPN2;APPL1
Positive Regulation of Macromolecule Biosynthetic Process (GO:0010557)	0.0020469	0.0868880	YAP1;GSK3B;MTPN;CNTF;DYRK2;CSF1;HPN;PTPN22;TNF;ROBO1;DLL4;EPM2AIP1;NWD1;UBR5;ITGB8;PPARGC1A;SLC38A2;WNT3;EGR1;NTRK2;XBP1;SOX11;BRAF;PAX6;ANK3;SORBS1;DKK1;F3;SIRT1;TGFBR1;CTCFL;TGFBR3;SFRP4;OLFM1;ACTA1;OCLN;FUBP3;GDNF;WT1;SOS1;TP53;MYD88;PF4;NFE2L2
Peptidyl-Serine Modification (GO:0018209)	0.0020626	0.0868880	GSK3B;CAB39;PRKCE;GALNT1;DYRK1A;STK39;PRKCA;TGFBR1;HIPK3;UHMK1;PDK3;MAPK1;POC1B-GALNT4;MAP3K13
Calcium Ion Transport Into Cytosol (GO:0060402)	0.0021160	0.0873202	RYR2;CACNA2D1;JPH1;SLC8A1
Ventricular Trabecula Myocardium Morphogenesis (GO:0003222)	0.0021160	0.0873202	DLL4;NRG1;RBPJ;TGFBR1
Negative Regulation of Signal Transduction (GO:0009968)	0.0022541	0.0898873	RB1;YWHAB;HHIP;MAPKAP1;CLEC12B;GATA3;NLK;TNF;CXXC4;DLL4;HHEX;SPRED1;HMGXB4;INPP5D;CCL5;UBR5;INPP5K;SH2B3;TNS2;APPL1;NCK1;SNCA;PTPN1;SEMA6A;PRKCB;FBXW7;SPRY4;DAB2IP;PTPN12;DKK1;SMAD5;DLK1;SFRP4;MMRN1;AGTR2;PTPN2
Axonogenesis (GO:0007409)	0.0022744	0.0898873	NOTCH2;PLPPR4;MYCBP2;DOCK7;SEMA3G;C9ORF72;SLITRK4;NPTN;SLIT2;ARHGEF40;EPHA5;SEMA6A;USP9X;DCC;APLP2;SEMA6D;SIAH1;ANK3;UNC5D;SEMA4G;ISL1;PAX2;KLF7;PALLD;FEZ2;CNTN1;SOS1;METAP1
Negative Regulation of Intracellular Signal Transduction (GO:1902532)	0.0022908	0.0898873	GSK3B;PHLPP1;RNF34;USP32;WWC1;WWC2;PRDM15;RORA;UBR1;TANK;IGF1R;PREX2;PKHD1;SPRED1;IRAK1;TSPAN6;MAP3K20;INPP5K;SLC39A8;SLIT2;RFFL;SH2B3;MINAR1;RUBCN;XBP1;FBXW7;DAB2IP;DUSP28;OPRM1;DKK1;SIRT1;CD2AP;TGFBR3;ITCH;DDIT4;PPIF;NF1;SNAI2
Positive Regulation of D-glucose Transmembrane Transport (GO:0010828)	0.0023023	0.0898873	OSBPL8;OCLN;BRAF;PTPN11;PIK3R1;SORBS1;RHOQ;APPL1
Protein Import Into Peroxisome Matrix (GO:0016558)	0.0023221	0.0898873	PEX16;PEX5L;USP9X;TRIM37;PEX13
Protein Deubiquitination (GO:0016579)	0.0023478	0.0898873	USP47;USP14;USP37;ZRANB1;USP38;USP6;USP42;USP32;USP9X;USP9Y;USP12;SPATA2;JOSD2;VCPIP1
Regulation of Apoptotic Process (GO:0042981)	0.0023627	0.0898873	FANK1;FOXA1;GSK3B;KIF14;TNF;IGF1R;CASP9;SLK;SIX4;TRIM2;EPC1;IFT57;USP42;DKK1;SIRT1;PAX2;RHOB;DNAJC3;OLFM1;SFRP4;MELK;CREB1;CEACAM5;KCNMA1;PPIF;PHIP;IL6ST;SOS1;FXN;TP53;NOTCH2;HDAC2;PHLPP1;ANP32A;MAPKAP1;DCUN1D3;HPN;TAF9;LRP2;PIK3R1;GLYAT;FOXO3;TANK;FOXO1;RTN4;HSP90B1;C3ORF38;PKHD1;BCL2L11;BAG3;ERBB4;MAP3K20;FLNA;RICTOR;SLIT2;ECT2;ATG7;SNCA;BARD1;EGR1;JUN;XBP1;SIAH2;SIAH1;DAB2IP;BRAF;SMAD5;HIPK3;ITCH;GDNF;WT1;PTPRA;IL7;STK17B;BRMS1L;NF1;PDCD2
Aortic Valve Morphogenesis (GO:0003180)	0.0023783	0.0898873	RB1;DLL4;SMAD2;ADAMTS5;JAG1;SNAI2;GATA3;SLIT2;ROBO1
Ventricular Septum Development (GO:0003281)	0.0023783	0.0898873	TGFBR3;HECTD1;SALL1;SOX11;MDM4;GATA3;SLIT2;TGFBR1;ROBO1
Positive Regulation of Cellular Component Biogenesis (GO:0044089)	0.0024814	0.0929176	SH3GLB1;GSK3B;PDCD6IP;TSG101;SYNPO2;DAB2IP;BRK1;NRG1;PRKCA;STAM;TNF;MTSS1;TGFBR1;BCL2L11;SUMO1;PFN2
Cellular Response to Ionizing Radiation (GO:0071479)	0.0025433	0.0929608	YAP1;BARD1;MAP3K20;GRB2;ECT2;TNF;SIRT1;TP53;TANK;RHOB;TSPYL5
Cell Morphogenesis Involved in Neuron Differentiation (GO:0048667)	0.0025886	0.0929608	RB1;MEF2A;DCC;APLP2;PLPPR4;SDC2;DOCK7;DCDC2;ANK3;ISL1;PAX2;KLF7;SLITRK4;TMEM106B;METAP1
Negative Regulation of Transforming Growth Factor Beta Receptor Signaling Pathway (GO:0030512)	0.0025886	0.0929608	FBN2;ZNF451;HDAC2;XBP1;TET1;ARID4B;LEMD3;SIRT1;VASN;SPRED1;SAP30L;BRMS1L;SRFBP1;OGT;SNX6
Cell Surface Receptor Protein Serine/Threonine Kinase Signaling Pathway (GO:0007178)	0.0025975	0.0929608	SMAD2;SMAD1;JUN;ACVR2B;SMAD5;ACVR2A;PIAS2
Positive Regulation of D-glucose Import (GO:0046326)	0.0025975	0.0929608	OSBPL8;OCLN;PTPN11;PIK3R1;SORBS1;RHOQ;APPL1
Negative Regulation of Macromolecule Biosynthetic Process (GO:0010558)	0.0026325	0.0931081	RB1;YAP1;NOTCH2;GSK3B;HDAC2;STK39;PTPN22;TNF;ROBO1;MECP2;DLL4;PPP2CB;INPP5K;FLOT2;ITGB8;ZNF148;CCR1;MSR1;FBXW7;SOX11;DICER1;SIRT1;SMAD5;FOXP1;OLFM1;OCLN;RBL1;PF4
Embryonic Digestive Tract Development (GO:0048566)	0.0026937	0.0931081	SALL1;RBPMS2;RARB;SOX11;PCSK5;TNF
Import Into Cell (GO:0098657)	0.0026937	0.0931081	SLC38A1;SLC1A3;ATP1A2;SPX;ACSL3;SLC29A1
Pulmonary Valve Morphogenesis (GO:0003184)	0.0026937	0.0931081	NOTCH2;SMAD2;ADAMTS5;JAG1;SLIT2;ROBO1
Negative Regulation of Developmental Process (GO:0051093)	0.0027205	0.0932367	SMAD2;SS18;SMARCD1;HDAC2;PARP1;SFMBT1;BCL11A;DCC;RC3H1;TET1;ARID4B;KLF7;RPS6KA6;ZNF706;NFIB;SAP30L;BRMS1L;OGT
Positive Regulation of Nucleocytoplasmic Transport (GO:0046824)	0.0027500	0.0934566	GSK3B;XBP1;BAG3;UBR5;ZIC1;EP300;FLNA;PIK3R1;EFCAB7;ECT2
Neuron Projection Morphogenesis (GO:0048812)	0.0030164	0.1016532	MEF2A;TSC22D4;DCC;APLP2;SDC2;PLPPR4;DOCK7;DAB2IP;DCDC2;ANK3;DICER1;LRP2;ISL1;PAX2;KLF7;ALCAM;TAOK3;SLITRK4;TMEM106B;ATP7A;PPP1R12B;METAP1;ZNF365
Regulation of Vascular Endothelial Growth Factor Receptor Signaling Pathway (GO:0030947)	0.0033148	0.1107873	PTPN1;HHEX;MMRN1;PRKCB;DAB2IP;MIA3;ROBO1
G Protein-Coupled Glutamate Receptor Signaling Pathway (GO:0007216)	0.0033784	0.1110781	GRM3;GRM5;GRM7;GNAQ;GRM8
Acidic Amino Acid Transport (GO:0015800)	0.0033784	0.1110781	SLC1A1;SLC7A14;SLC1A3;SLC6A1;KCNK2
Positive Regulation of Stem Cell Population Maintenance (GO:1902459)	0.0034572	0.1126469	SMARCD1;SS18;HDAC2;SAP30L;BRMS1L;TET1;ARID4B;ARID1A;OGT
Ventricular Cardiac Muscle Tissue Morphogenesis (GO:0055010)	0.0035171	0.1126469	DLL4;TGFBR3;RYR2;NRG1;LRP2;RBPJ;ISL1;TGFBR1
Adrenal Gland Development (GO:0030325)	0.0035708	0.1126469	SALL1;MDK;WT1;NF1
Cell Differentiation in Spinal Cord (GO:0021515)	0.0035708	0.1126469	SOX13;MDGA2;SOX6;ISL1
Cardiac Ventricle Development (GO:0003231)	0.0035933	0.1126469	DLL4;HECTD1;SALL1;TMEM65;MDM4;GATA3
Positive Regulation of Macrophage Migration (GO:1905523)	0.0035933	0.1126469	P2RY12;CSF1;MDK;CCL5;MAPK1;RTN4
Positive Regulation of Signal Transduction (GO:0009967)	0.0038067	0.1184190	CD80;MIA3;GATA3;RBPJ;NLK;CDKN2AIP;ROBO1;HSP90B1;DLL4;GRIN2A;ESM1;HHEX;SALL1;CCL5;NPTN;RSPO3;FLNA;ZRANB1;JAG1;CRBN;PRKCB;DAB2IP;DCDC2;PRKCA;LAMB1;PTPN11;SORBS1;F3;SIRT1;TGFBR1;IL7;PHIP;IL6ST
Monoatomic Anion Transmembrane Transport (GO:0098656)	0.0039651	0.1223708	GABRB2;GABRA1;GABRA5;GABRA4;SLC1A1;SLC1A3;SLC6A1;ANO5;GABRG1;LRRC8D;SLC26A7;SLC12A6;KCNK2

Cellular Response to Fibroblast Growth Factor Stimulus (GO:0044344)	0.0039942	0.1223708	DLL4;KL;NR4A1;FGF7;CHURC1;FGF14;CCL5;FRS2;NDNF;PTPN11;SHCBP1
Positive Regulation of Neurogenesis (GO:0050769)	0.0040295	0.1225229	FXR1;NTRK2;SS18L1;CAPRIN1;NPTN;NUMB;SOX11;PLXNC1;OPRM1;LRP2;SLIT2;ROBO1
Regulation of Keratinocyte Proliferation (GO:0010837)	0.0041737	0.1241383	NOTCH2;FGF7;MDK;CASK;SNAI2;REG3G;SRSF6
Axon Guidance (GO:0007411)	0.0042537	0.1241383	EPHA5;NOTCH2;SEMA6A;DCC;SEMA6D;MYCBP2;SIAH1;SEMA3G;UNC5D;SEMA4G;CELSR3;KLF7;ALCAM;GDNF;PALLD;NFIB;FEZ2;CNTN1;NPTN;SLIT2;ARHGEF40;SOS1
Positive Regulation of Cell Population Proliferation (GO:0008284)	0.0042844	0.1241383	CSF1;KIF14;RBPJ;TNF;IGF1R;GHR;FGF7;ESM1;MDK;RBPMS2;CCN4;SS18;CNOT6L;SOX11;NRG1;SIRT1;PAX2;TGFBR1;FOXP1;EREG;BTC;TGFBR3;CCKBR;PHIP;IL6ST;FXN;TSPYL5;HBEGF;HDAC4;YAP1;SMARCD1;HDAC2;TTK;REG3G;PIK3R1;CSF2RA;PKHD1;DLL4;ERBB4;CCL5;ZNF703;CXCR2;S1PR3;NTRK2;XBP1;LAMB1;EPOR;EPGN;KITLG;GDNF;IL7;TNFSF4;DNAJA2;ACER3;HPSE
ERBB4 Signaling Pathway (GO:0038130)	0.0043896	0.1241383	ERBB4;NRG1;EREG
Epithelial Cell Differentiation Involved in Kidney Development (GO:0035850)	0.0043896	0.1241383	SALL1;MTSS1;PAX2
Negative Regulation of Cardiac Muscle Cell Differentiation (GO:2000726)	0.0043896	0.1241383	FRS2;SOX6;DKK1
Negative Regulation of Chemokine-Mediated Signaling Pathway (GO:0070100)	0.0043896	0.1241383	SLIT2;SH2B3;ROBO1
Negative Regulation of Mast Cell Activation (GO:0033004)	0.0043896	0.1241383	CD84;ENPP3;CD300LF
Regulation of Stress Granule Assembly (GO:0062028)	0.0043896	0.1241383	USP32;G3BP1;G3BP2
Ventricular Compact Myocardium Morphogenesis (GO:0003223)	0.0043896	0.1241383	TGFBR3;LRP2;TGFBR1
Positive Regulation of Smooth Muscle Cell Proliferation (GO:0048661)	0.0044650	0.1249242	JUN;XBP1;CCL5;RBPMS2;CCN4;PIK3R1;TNF;MEF2D;EREG;FOXP1
Chloride Transmembrane Transport (GO:1902476)	0.0045936	0.1249242	GABRB2;GABRA1;GABRA5;GABRA4;SLC1A1;SLC1A3;SLC26A7;SLC6A1;SLC12A6;ANO5;KCNK2;GABRG1
Positive Regulation of Organelle Assembly (GO:1902117)	0.0045936	0.1249242	SH3GLB1;GSK3B;SNX4;CCDC88A;CNOT6L;PDCD6IP;CAPRIN1;G3BP1;RAB3IP;G3BP2;CCP110;TNF
Regulation of Cell-Matrix Adhesion (GO:0001952)	0.0045936	0.1249242	PKHD1;GSK3B;SLK;JAG1;CSF1;PTPRA;NEXMIF;NF1;CASK;UTRN;CCL28;GPM6B
Cellular Response to Interleukin-1 (GO:0071347)	0.0046027	0.1249242	EGR1;IRAK1;USP32;TRAF6;CCL5;DAB2IP;RC3H1;PLCB1;MAP3K7;TANK;MYD88
mRNA Stabilization (GO:0048255)	0.0046027	0.1249242	SYNCRIP;BOLL;GDNF;CSDE1;IREB2;ELAVL4;HNRNPD;FAM76B;TENT5A;ELAVL1;MYD88
Regulation of Cardiac Muscle Cell Proliferation (GO:0060043)	0.0046980	0.1266585	TGFBR3;ERBB4;NRG1;RBPJ;TGFBR1;RUNX1
Golgi to Endosome Transport (GO:0006895)	0.0047405	0.1269577	SYS1;SORT1;VPS13C;VPS13A;EPS15
Inorganic Anion Transmembrane Transport (GO:0098661)	0.0048200	0.1274009	GABRB2;GABRA1;SLC20A1;GABRA5;GABRA4;SLC1A1;SLC1A3;SLC6A1;ANO5;GABRG1;SLC26A7;SLC12A6;SLC37A3;KCNK2
Response to Insulin (GO:0032868)	0.0048200	0.1274009	XBP1;PARP1;SORT1;MAPKAP1;INHBB;PIK3R1;SORBS1;SIRT1;FOXO1;KAT2B;RAB31;INPP5K;OGT;RHOQ
Regulation of Cell Population Proliferation (GO:0042127)	0.0050217	0.1318698	CSF1;WWC1;WWC2;KIF14;RERG;IGF1R;GHR;HHEX;FGF7;ESM1;SIX4;ARID2;TNS2;MINAR1;SS18;CNOT6L;NRG1;OPRM1;SIRT1;TGFBR1;EREG;RUNX1;BTC;SFRP4;KAT2B;CCKBR;PHIP;SGK3;IL6ST;SOS1;FXN;TP53;TSPYL5;HBEGF;YAP1;HDAC4;SMARCD1;HDAC2;CDCA7;TTK;GATA3;REG3G;PDS5B;CSF2RA;TOB1;PKHD1;DLL4;CYP27B1;CLMN;ERBB4;NACC2;ZNF703;CXCR2;S1PR3;SH2B3;ZBTB7C;FKTN;NTRK2;JUN;XBP1;JAG1;DAB2IP;SOD2;PTPN14;SHCBP1;EPOR;CCDC88A;FABP3;EPGN;KITLG;GDNF;WT1;IL7;TNFSF4;ACER3;DNAJA2;STRN;MDM4;ZNF777;PTPN2;PF4
Regulation of Transmembrane Receptor Protein Serine/Threonine Kinase Signaling Pathway (GO:0090092)	0.0051907	0.1344331	SMAD2;TGFBR3;SFRP4;ZNF703;SOX11;NREP;PCSK6
Toll-Like Receptor 4 Signaling Pathway (GO:0034142)	0.0051907	0.1344331	IRAK1;TRIL;PRKCE;TRAF6;RAB11FIP2;MAP3K7;MYD88
Regulation of Neurogenesis (GO:0050767)	0.0052190	0.1344331	YAP1;FXR1;DOCK7;BHLHE41;NUMB;PAX6;SOX11;OPRM1;LRP2;HOOK3;TNF;WNT3
Chromatin Remodeling (GO:0006338)	0.0054320	0.1384549	RB1;FOXA1;KDM5A;HDAC4;KDM5B;NFAT5;SMARCD1;HDAC2;INO80D;ZBTB1;EHMT1;PSIP1;CHD1;MIER1;UBR5;GATAD1;PWWP2A;JARID2;ARID2;BARD1;SS18;KDM2B;TET1;CHD1L;UBE2A;ARID1A;SIRT1;ARID1B;RNF168;KAT2B;MTF2;TRIP12;ASF1A
Cell Proliferation in Forebrain (GO:0021846)	0.0055806	0.1384549	DOCK7;KIF14;NUMB;HOOK3
Mesenchymal to Epithelial Transition (GO:0060231)	0.0055806	0.1384549	SALL1;WT1;GATA3;PAX2
Negative Regulation of Vascular Endothelial Growth Factor Receptor Signaling Pathway (GO:0030948)	0.0055806	0.1384549	PTPN1;HHEX;MMRN1;DAB2IP
Positive Regulation of Gluconeogenesis (GO:0045722)	0.0055806	0.1384549	PPARGC1A;SIRT1;FOXO1;PTPN2
Regulation of Mesenchymal Stem Cell Differentiation (GO:2000739)	0.0055806	0.1384549	GSK3B;SOX6;WNT3;SOX5
RNA Biosynthetic Process (GO:0032774)	0.0057444	0.1393679	MEF2A;SMAD2;SMAD1;THRB;FASTKD2;CELF2;ELAVL4;TTF2;SYNCRIP;ATXN1;POLR3A;FUBP3;HNRNPH1;NOVA1;HNRNPF;XRN2;MED20;HNRNPD;EPC1
Negative Regulation of mRNA Catabolic Process (GO:1902373)	0.0057553	0.1393679	FXR1;SYNCRIP;GDNF;CSDE1;IREB2;HNRNPD;FAM76B;TENT5A;ELAVL1
Positive Regulation of Biomineral Tissue Development (GO:0070169)	0.0057553	0.1393679	FBN2;KL;ATP2B1;TENT5A;ACVR2B;ODAPH;SLC8A1;GPM6B;ACVR2A
Positive Regulation of Gene Expression, Epigenetic (GO:0141137)	0.0057553	0.1393679	SMARCD1;KAT2B;KMT2A;GDNF;EP300;ARID1A;ARID1B;TP53;CTCFL
Insulin Receptor Signaling Pathway (GO:0008286)	0.0060117	0.1443730	GSK3B;PHIP;GRB2;PIK3R1;SORBS1;RHOQ;FOXO1;PTPN2;IGF1R;APPL1
Regulation of Cardiac Muscle Contraction by Calcium Ion Signaling (GO:0010882)	0.0060333	0.1443730	RYR2;ASPH;ATP1A2;PRKCA;ATP1B1;SLC8A1
Peptidyl-Threonine Phosphorylation (GO:0018107)	0.0062026	0.1475509	GDNF;CAMK2A;STK39;DYRK1A;MAPK1;PRKCA;NLK;HIPK3
Synaptic Transmission, GABAergic (GO:0051932)	0.0064510	0.1517739	GABRA1;GABRB2;GABRA5;GABRA4;GABRG1
Cytokine-Mediated Signaling Pathway (GO:0019221)	0.0064552	0.1517739	CNTF;PIK3R1;FOXO3;TANK;TNF;CSF2RA;EDA2R;GHR;IRAK1;CCL5;CXCR2;CD300LF;MAP3K7;APPL1;CCR1;EGR1;CHUK;IL36G;PRKCA;PTPN11;F3;SIRT1;EPOR;EREG;IL7;TRAF6;PLCB1;IL6ST;SOS1;TP53;MYD88;PF4
Peptidyl-Threonine Modification (GO:0018210)	0.0067377	0.1575008	GDNF;GALNT1;STK39;DYRK1A;MAPK1;PRKCA;POC1B-GALNT4;NLK;HIPK3
Regulation of Hydrolase Activity (GO:0051336)	0.0069269	0.1609925	ARHGAP44;ATP5IF1;EPHA5;NTRK2;RABGAP1;CAMK2A;DAB2IP;SIRT1;TBC1D15;FGD2
Regulation of BMP Signaling Pathway (GO:0030510)	0.0071192	0.1645154	SMAD2;UBE2D3;CRIM1;SOX11;LRP2;PCSK6;RBPJ;LEMD3;GDF5;SFRP4;RBPMS2;VWC2;GPR155
Cellular Response to Nitrogen Compound (GO:1901699)	0.0075050	0.1685037	P2RY12;EGR1;GABRB2;GSK3B;XBP1;CACNA2D1;CACNA1A;PTPN22;AHR;UBR1;ICAM1;IGF1R;HSP90B1;CACNB1;GRIN2A;GRM5;WT1;EP300;INPP5K;SLIT2;TP53;SNX6;CHMP5
Negative Regulation of DNA-binding Transcription Factor Activity (GO:0043433)	0.0075113	0.1685037	RB1;HDAC4;HDAC2;CHUK;FZD6;DAB2IP;SIRT1;PIAS2;PKHD1;SFRP4;PPP2CB;NWD1;ITCH;SUMO1;TNFSF4;BRMS1L;FLNA;TRIM37
Cellular Response to Growth Factor Stimulus (GO:0071363)	0.0075299	0.1685037	XBP1;SEMA6A;KIDINS220;DAB2IP;NDNF;PTPN11;LRP2;PTPN12;NR3C1;ACVR2B;TGFBR1;ACVR2A;DLL4;NR4A1;CCL5;INPP5K;SNAI2;SOX6;SOS1;SOX5;APPL1
Positive Regulation of Programmed Cell Death (GO:0043068)	0.0076709	0.1685037	FANK1;FOXA1;DCUN1D3;FOXO3;TNF;TMEM164;FOXO1;CASP9;C3ORF38;BCL2L11;MAP3K20;SLIT2;ECT2;ATG7;SNCA;BARD1;JUN;SIAH1;DAB2IP;SIRT1;RHOB;SFRP4;OLFM1;MELK;WT1;PTPRA;STK17B;KCNMA1;NF1;PDCD2;TP53
Negative Regulation of Ossification (GO:0030279)	0.0077674	0.1685037	CCR1;SMAD2;BMP3;MDK;RBPJ;ACVR2B;DKK1

Negative Regulation of Programmed Cell Death (GO:0043069)	0.0078182	0.1685037	FANK1;NOTCH2;GSK3B;HDAC2;MAPKAP1;HPN;TAF9;KIF14;PIK3R1;GLYAT;LRP2;FOXO1;IGF1R;HSP90B1;PKHD1;SIX4;BAG3;ERBB4;FLNA;RICTOR;SNCA;BARD1;XBP1;SIAH2;BRAF;DKK1;SIRT1;SMAD5;PAX2;HIPK3;DNAJC3;ITCH;CREB1;GDNF;WT1;IL7;CEACAM5;PPIF;PHIP;IL6ST;SOS1;TP53;FXN;NFE2L2
Response to Amyloid-Beta (GO:1904645)	0.0078438	0.1685037	CACNB1;GSK3B;GRIN2A;GRM5;CACNA2D1;CACNA1A;ICAM1;IGF1R;SNX6
Anterograde Trans-Synaptic Signaling (GO:0098916)	0.0078508	0.1685037	GRIA2;GABRB2;CHRNA5;SLC1A1;CACNA1A;SLC1A3;GRIK1;SLC6A1;GRM3;GRIN2A;GRM7;MPZ;HRH4;MAPK1;DLGAP1;SLC12A6;SNCA;DTNA;SYN2;CACNB1;DLG1;CACNB4;SYT10;AMPH;PDE7B
Regulation of Phosphatidylinositol 3-Kinase/Protein Kinase B Signal Transduction (GO:0051896)	0.0081037	0.1685037	PHLPP1;GATA3;TNF;RTN4;IGF1R;PKHD1;CCL5;INPP5K;PIP5K1B;RICTOR;SH2B3;P2RY12;OSBPL8;RUBCN;NTRK2;XBP1;DAB2IP;PRKCA;PTPN11;SIRT1;TGFBR1;CCDC88A;GFRAL;HPSE;DIPK2A;SOS1;TSPYL5;HBEGF
Actin Crosslink Formation (GO:0051764)	0.0082248	0.1685037	GAS2L3;BAIAP2L1;DPYSL3;FLNA
Endoplasmic Reticulum Membrane Organization (GO:0090158)	0.0082248	0.1685037	VCPIP1;REEP5;RTN4;TRDN
Insulin-Like Growth Factor Receptor Signaling Pathway (GO:0048009)	0.0082248	0.1685037	GHR;PIK3R1;PLCB1;IGF1R
Negative Regulation of Myotube Differentiation (GO:0010832)	0.0082248	0.1685037	HDAC4;XBP1;CEACAM5;BHLHE41
Positive Regulation of Oxidoreductase Activity (GO:0051353)	0.0082248	0.1685037	GDNF;ABL2;VDAC2;FXN
Regulation of Cardiac Muscle Cell Apoptotic Process (GO:0010665)	0.0082248	0.1685037	MDK;PPP1R10;CAMK2A;NRG1
Regulation of Programmed Necrotic Cell Death (GO:0062098)	0.0082248	0.1685037	ITCH;PELI1;SPATA2;OGT
IRE1-mediated Unfolded Protein Response (GO:0036498)	0.0082631	0.1685037	PTPN1;XBP1;PARP16
Cellular Hypotonic Response (GO:0071476)	0.0082631	0.1685037	CAB39;STK39;SLC12A6
Negative Regulation of Cardiac Muscle Cell Apoptotic Process (GO:0010667)	0.0082631	0.1685037	MDK;PPP1R10;NRG1
Negative Regulation of Cardiocyte Differentiation (GO:1905208)	0.0082631	0.1685037	FRS2;SOX6;DKK1
Negative Regulation of Glycogen Biosynthetic Process (GO:0045719)	0.0082631	0.1685037	GSK3B;STK39;INPP5K
Positive Regulation of Animal Organ Morphogenesis (GO:0110110)	0.0082631	0.1685037	GDNF;SIX4;GATA3
Positive Regulation of Hormone Metabolic Process (GO:0032352)	0.0082631	0.1685037	HPN;MIA3;GATA3
Response to Lipopolysaccharide (GO:0032496)	0.0083938	0.1685037	XBP1;CD80;DAB2IP;IL36G;PTPN22;PPBP;PPM1E;FOXP1;CYP27B1;NR4A1;CASP7;IRAK1;TNFSF4;TRAF6;PELI1;ADAM9;CCL28;MYD88;PF4V1;CHMP5;SNCA;PF4
Peptidyl-Serine Phosphorylation (GO:0018105)	0.0084271	0.1685037	GSK3B;CAB39;PRKCE;DYRK1A;STK39;PDK3;MAPK1;PRKCA;MAP3K13;TGFBR1;HIPK3;UHMK1
Negative Regulation of Cation Transmembrane Transport (GO:1904063)	0.0085515	0.1685037	CAB39;PRKCE;STK39;PPIF;ATP1A2
Positive Regulation by Host of Viral Transcription (GO:0043923)	0.0085515	0.1685037	JUN;CCNT2;HPN;EP300;CHD1
Positive Regulation of Leukocyte Adhesion to Vascular Endothelial Cell (GO:1904996)	0.0085515	0.1685037	NFAT5;IRAK1;MDK;TRAF6;TNF
Positive Regulation of Smooth Muscle Cell Migration (GO:0014911)	0.0085515	0.1685037	XBP1;MDK;CCL5;DOCK7;CCN4
Reg of Cardiac Muscle Contraction by Reg of the Release of Sequestered Calcium Ion (GO:0010881)	0.0085515	0.1685037	RYR2;ASPH;ATP1A2;PRKCA;SLC8A1
Negative Regulation of Cell Differentiation (GO:0045596)	0.0086049	0.1685037	RB1;YAP1;FOXA1;GSK3B;SMARCD1;HPN;TMEM182;RORA;GATA3;TNF;FOXO1;RTN4;SPRED1;RUNX1T1;SMAD2;SS18;JAG1;ABCA5;DAB2IP;CRIM1;PTPN11;TMEM64;GDF5;SIRT1;VASN;TGFBR1;COL5A2;SNAI2
Nervous System Development (GO:0007399)	0.0087009	0.1685037	GMFB;NCAN;GRIK1;MTR;HAPLN1;ROBO1;DMBX1;FGF7;RPS6KA6;ZIC2;MDK;ZIC1;EP300;PLXNC1;JARID2;SOX6;TRPC5;SEMA6A;APLP2;DYRK1A;SOX11;PAX6;NRG1;PAX5;PAX2;TGFBR1;BCAN;OLFM1;ZEB1;B3GNT5;FEZ2;GFRAL;HOXB2;NOTCH2;HDAC4;BTBD1;ERBB4;ST8SIA4;ECT2;GPM6B;FKTN;JAG1;MOG;SIAH1;LSAMP;CRIM1;ZIC5;PTPRD;DLG1;FGF14;GDNF;NF1;MDGA2
Nuclear Transport (GO:0051169)	0.0087085	0.1685037	GLE1;AHCTF1;POM121;ANP32A;NUP210;RSRC1;NUP43;NUP58
Positive Regulation of Bone Mineralization (GO:0030501)	0.0087085	0.1685037	FBN2;KL;ATP2B1;TENT5A;ACVR2B;SLC8A1;GPM6B;ACVR2A
Protein Modification Process (GO:0036211)	0.0088145	0.1697427	GSK3B;GMFB;DYRK2;UBE2D3;F13A1;PTPN22;GMPPB;SERP1;MAP3K8;SEPHS1;ZNF367;USP6;PARP1;PRKCB;PRKCE;DYRK1A;PRKCA;DUSP28;DUSP9;FBXW2;CREB1;PRKD3;B3GNT5;SGK3;CAMK2A;STK39;DAPP1;NLK;MTM1;PPM1G;PPP2CB;FUT9;MAP3K20;ST8SIA4;ABL2;MAPK1;ATG7;FKTN;CDK17;PTPN1;YES1;CSNK1A1;ST8SIA3;BRAF;PTPN12;SSH2;PTPN14;HIPK3;MAPK10;TMEM59;PTPRB;SNRK;TAOK3;CAMK4;STK17B;UBA3;MAP3K13;PTPN4;METAP1
T Cell Differentiation (GO:0030217)	0.0090828	0.1735752	DLL4;LFNG;PNKD;SOX13;RABL3;PTPN22;GATA3;PIK3R1;PTPN2
Regulation of Signal Transduction (GO:0009966)	0.0090993	0.1735752	HHIP;CD80;ZDHHC7;RORA;GATA3;PPM1K;SAMSN1;CDKN2AIP;LFNG;RGS4;RGS5;IRAK1;POFUT1;INPP5D;PPP6R3;ZIC1;NCK1;PTPN1;DCDC2;ADCYAP1;DBNDD2;GDNF;STRN;CDK13;PLCB1;OGT;PTPN2
Positive Regulation of Multicellular Organismal Process (GO:0051240)	0.0092831	0.1738031	SMARCD1;HDAC2;EHMT1;ARID4B;TNF;RTN4;IGF1R;GHR;CYP27B1;SNX4;SIX4;MDK;SAP30L;ZNF703;INPP5K;SOX6;PPARGC1A;SOX5;SMAD2;SS18;XBP1;CADM1;FGG;RBM19;RFX3;TET1;PRKCA;SOX11;ESRRG;PTPN11;INHBB;TMEM64;ARID1A;SIRT1;TGFBR1;EREG;CACNB1;ADCYAP1;OLFM1;GDNF;TNFSF4;BRMS1L;ZNF516;PLCB1;MAP3K13;OGT
Negative Regulation of Insulin Receptor Signaling Pathway (GO:0046627)	0.0093614	0.1738031	PTPN1;PRKCB;MAPKAP1;INPP5K;TNS2;PTPN2;NCK1
Regulation of Dendritic Spine Morphogenesis (GO:0061001)	0.0093614	0.1738031	ARHGAP44;PTPRD;CAPRIN1;PDLIM5;LZTS3;PPFIA2;ZDHHC15
Positive Regulation of Apoptotic Process (GO:0043065)	0.0093803	0.1738031	FANK1;FOXA1;GSK3B;DCUN1D3;FOXO3;TNF;FOXO1;CASP9;C3ORF38;CASP7;BCL2L11;CASP3;MAP3K20;SLIT2;ECT2;ATG7;SNCA;BARD1;JUN;XBP1;SIAH1;DAB2IP;F3;SIRT1;TGFBR1;RHOB;SFRP4;OLFM1;MELK;WT1;PTPRA;STK17B;BRMS1L;KCNMA1;NF1;PDCD2;TP53
Myotube Differentiation (GO:0014902)	0.0094981	0.1738031	ADAMTS5;MYEF2;SIX4;SORT1;ADGRB3;TMEM182
Negative Regulation of Extrinsic Apoptotic Signaling Pathway in Absence of Ligand (GO:2001240)	0.0094981	0.1738031	GDNF;EYA4;SNAI2;NRG1;TNF;PF4
Negative Regulation of Signal Transduction in Absence of Ligand (GO:1901099)	0.0094981	0.1738031	GDNF;EYA4;SNAI2;NRG1;TNF;PF4
Positive Regulation of Cell Communication (GO:0010647)	0.0094981	0.1738031	CD80;SOX11;ANK3;GATA3;TNF;CDKN2AIP
Regulation of Release of Sequestered Calcium Ion Into Cytosol by Sarcoplasmic Reticulum (GO:0010880)	0.0094981	0.1738031	RYR2;ASPH;ATP1A2;PRKCA;SLC8A1;TRDN
Cellular Response to Lipopolysaccharide (GO:0071222)	0.0096130	0.1751130	XBP1;CD80;PRKCE;DAB2IP;IL36G;PTPN22;PPBP;PPM1E;CASP7;IRAK1;TNFSF4;TRAF6;ADAM9;CCL28;MYD88;PF4V1;CHMP5;PF4
Protein Complex Oligomerization (GO:0051259)	0.0098278	0.1782236	TMEM120B;PDCD6IP;ATL3;ZBTB1;CALHM3;ELAVL1;RNF135;CALHM1;WDCP;SPAST;NACC2;G3BP2;LRRC8D;ZNF777;POLK;ECT2;SNCA
Positive Regulation of Neuron Apoptotic Process (GO:0043525)	0.0102131	0.1828862	CASP9;GSK3B;CASP7;FBXW7;CASP3;NF1;FOXO3;TNF
Regulation of Axon Extension (GO:0030516)	0.0102131	0.1828862	GSK3B;OLFM1;BCL11A;SEMA6D;SEMA3G;DIP2B;MAP3K13;RTN4
DNA-templated Transcription (GO:0006351)	0.0102206	0.1828862	NFAT5;DDX5;CCNT2;THRB;TTF2;FOXO3;RO60;EPC1;PKNOX1;POLR2K;SOX5;MEF2A;SMAD2;SMAD1;XBP1;PARP1;GTF2H1;ETV1;PAX5;GTF2F1;HIPK3;EREG;POLR3A;FUBP3;MED20;TP53
Endoplasmic Reticulum Organization (GO:0007029)	0.0103277	0.1839879	VCPIP1;ATL3;REEP5;VAPA;TMEM33;SEC16A;REEP3;MIA3;RTN4;TRDN

BMP Signaling Pathway (GO:0030509)	0.0104643	0.1847942	TGFBR3;SMAD1;DDX5;USP9X;USP9Y;ACVR2B;GDF5;SMAD5;ACVR2A
Cellular Response to Radiation (GO:0071478)	0.0104643	0.1847942	BARD1;GPR88;GRB2;ECT2;TANK;TNF;SIRT1;TP53;RHOB
Cytoskeleton Organization (GO:0007010)	0.0106753	0.1868586	ZRANB1;SEMA6A;MAPKAP1;LARP4;RHOBTB1;FGD2;FMNL3;PCLO;MDK;PALLD;NAA25;PHIP;RICTOR;BRWD1;PDZD8;BRWD3;ZNF135
Protein Modification by Small Protein Removal (GO:0070646)	0.0108551	0.1868586	USP47;USP37;ZRANB1;USP6;TOR1A;USP42;USP32;USP9X;USP9Y;USP12;JOSD2;SENP7;VCPIP1
Positive Regulation of Canonical NF-kappaB Signal Transduction (GO:0043123)	0.0108583	0.1868586	ANKRD17;NFAT5;SLC20A1;RORA;TANK;TNF;EDA2R;TRIM8;IRAK1;TSPAN6;MIER1;FLNA;FLOT2;ECT2;MAP3K7;MAP3K3;CARD10;PARP1;CHUK;PRKCB;DAB2IP;ZDHHC13;VAPA;TRAF6;PELI1;MYD88
Cytosolic Transport (GO:0016482)	0.0109941	0.1868586	SYS1;STX16;SORT1;VPS13C;GOSR1;RHOBTB3;VPS13A;RBSN;AP1G1;VPS54;DENND5A;TBC1D17;RAB6B;EPS15;SNX6
SMAD Protein Signal Transduction (GO:0060395)	0.0110814	0.1868586	SMAD2;SMAD1;JUN;SMAD5;PIAS2
Positive Regulation of Muscle Hypertrophy (GO:0014742)	0.0110814	0.1868586	MEF2A;MTPN;PARP1;PRKCA;IL6ST
Regulation of Adaptive Immune Response (GO:0002819)	0.0110814	0.1868586	TNFSF4;AHR;IL6ST;SAMSN1;SIRT1
Response to Axon Injury (GO:0048678)	0.0110814	0.1868586	P2RY12;RTN4RL1;DPYSL3;MTR;NREP
Ureteric Bud Morphogenesis (GO:0060675)	0.0110814	0.1868586	SALL1;GDNF;WT1;GATA3;PAX2
Negative Regulation of Cellular Response to Insulin Stimulus (GO:1900077)	0.0111819	0.1868586	PTPN1;PRKCB;MAPKAP1;INPP5K;TNS2;PTPN2;NCK1
Negative Regulation of Proteasomal Ubiquitin-Dependent Protein Catabolic Process (GO:0032435)	0.0111819	0.1868586	PHF20L1;UBXN1;USP38;USP9X;TAF9;OGT;METAP1
Protein Localization to Golgi Apparatus (GO:0034067)	0.0111819	0.1868586	SYS1;VPS13C;VPS13A;ARL5A;RAB6B;BICD2;ZDHHC15
Response to Alcohol (GO:0097305)	0.0111819	0.1868586	GHR;SMAD2;TGFBR3;GRIN2A;GNAQ;SLIT2;TGFBR1
Negative Regulation of Transcription by Competitive Promoter Binding (GO:0010944)	0.0115731	0.1910279	HDAC4;HDAC2;HHEX;CREB1
Positive Regulation of Keratinocyte Proliferation (GO:0010838)	0.0115731	0.1910279	NOTCH2;FGF7;MDK;REG3G
Sodium Ion Export Across Plasma Membrane (GO:0036376)	0.0115731	0.1910279	ATP1A2;ATP1B1;SLC4A4;SLC8A1
Regulation of Protein Export From Nucleus (GO:0046825)	0.0116772	0.1919630	BARD1;GSK3B;BAG3;FAM76B;PTPN14;UHMK1
Positive Regulation of Lipid Biosynthetic Process (GO:0046889)	0.0119021	0.1948674	KAT2B;XBP1;FABP3;CREB1;MID1IP1;CNEP1R1;ZBTB20;SORBS1
Protein K48-linked Ubiquitination (GO:0070936)	0.0120784	0.1969571	KCMF1;RNF34;UBE3C;UBE2D3;MYCBP2;UBE2A;DTX4;ANAPC10;ITCH;KBTBD6;UBR5;PELI1;RFFL;FBXO9
Negative Regulation of Response to Stimulus (GO:0048585)	0.0132155	0.2125659	PTPN1;KLF7;MCTP1;HHIP;INPP5D;CASK;SLIT2;SIRT1;C9ORF72;SH2B3
Glucose Homeostasis (GO:0042593)	0.0132394	0.2125659	FBN2;TSC22D4;KCNB1;PAX6;PIK3R1;HOOK3;SIRT1;FOXO1;KLF7;INPP5K;LRRC8D;SELENOT;PPARGC1A;PTPN2
Coronary Vasculature Morphogenesis (GO:0060977)	0.0136138	0.2125659	TGFBR3;SPRED1;TGFBR1
Hypotonic Response (GO:0006971)	0.0136138	0.2125659	CAB39;STK39;SLC12A6
Kidney Epithelium Development (GO:0072073)	0.0136138	0.2125659	SALL1;WT1;PAX2
Kidney Mesenchyme Development (GO:0072074)	0.0136138	0.2125659	SIX4;WT1;PAX2
Negative Regulation of p38MAPK Cascade (GO:1903753)	0.0136138	0.2125659	DLG1;DUSP10;PTPN22
Neurotrophin TRK Receptor Signaling Pathway (GO:0048011)	0.0136138	0.2125659	SORT1;CASP3;DDIT4
Regulation of Chemokine-Mediated Signaling Pathway (GO:0070099)	0.0136138	0.2125659	SLIT2;SH2B3;ROBO1
Regulation of Dopamine Uptake Involved in Synaptic Transmission (GO:0051584)	0.0136138	0.2125659	TOR1A;GDNF;SNCA
Regulation of miRNA-mediated Gene Silencing (GO:0060964)	0.0136138	0.2125659	FXR1;PUM1;ELAVL1
Connective Tissue Development (GO:0061448)	0.0137877	0.2144525	XBP1;SPTLC2;ZNF516;ITGB8;NFATC2;ATP7A;SOX6;SOX5
Positive Regulation of T Cell Activation (GO:0050870)	0.0139639	0.2148201	SMARCD1;XBP1;CD80;ZBTB1;GATA3;ARID1A;ARID1B;IL7;MDK;TNFSF4;CCL5;ABL2;FLOT2;ARID2;IL6ST;NCK1
Positive Regulation of MAP Kinase Activity (GO:0043406)	0.0139802	0.2148201	GHR;PTPN1;TAOK3;TRAF6;DAB2IP;MAP3K13;TNF;MAP3K7;TANK;MAP3K4;ROBO1
Cardiac Epithelial to Mesenchymal Transition (GO:0060317)	0.0140769	0.2148201	TGFBR3;OLFM1;SNAI2;RBPJ;TGFBR1
Mitotic Sister Chromatid Cohesion (GO:0007064)	0.0140769	0.2148201	RB1;RAD21;PDS5B;HDAC8;PDS5A
Protein Localization to Endoplasmic Reticulum (GO:0070972)	0.0140769	0.2148201	RYR2;VAPA;SEC16A;BCAP29;MIA3
Regulation of Cytoplasmic Translation (GO:2000765)	0.0141860	0.2148622	SYNCRIP;CSDE1;HNRNPD;PARP16;NMNAT2;CPEB4
Regulation of Glycogen Biosynthetic Process (GO:0005979)	0.0141860	0.2148622	GSK3B;EPM2AIP1;DYRK2;STK39;INPP5K;SORBS1
Positive Regulation of Protein Localization to Nucleus (GO:1900182)	0.0143940	0.2171990	YAP1;CARD10;XBP1;BAG3;ZIC1;UBR5;EP300;FLNA;TTF2;PIK3R1;EFCAB7;ECT2
Chemical Synaptic Transmission (GO:0007268)	0.0145938	0.2193953	GABRB2;GRIA2;CHRNA5;SLC1A1;CACNA1A;SLC1A3;GRIK1;SLC6A1;GRM3;GRIN2A;GRM7;MPZ;HRH4;MAPK1;DLGAP1;SLC12A6;GRIA3;SNCA;GABRA1;UNC13A;DTNA;GABRA5;GABRA4;SYN2;GABRG1;CACNB1;DLG1;CACNB4;SYT10;AMPH;PDE7B
Negative Regulation of Cell Growth (GO:0030308)	0.0146591	0.2195621	RB1;MINAR1;BCL11A;DCC;SEMA6D;DCUN1D3;SEMA3G;CDKN2AIP;RTN4;RERG;ST7L;CYP27B1;WT1;DIP2B;AGTR2;SLIT2;TP53
Regulation of Wnt Signaling Pathway (GO:0030111)	0.0151316	0.2258023	AMER2;ZRANB1;CRBN;DCDC2;NLK;DKK1;CXXC4;HSP90B1;SFRP4;HHEX;SALL1;HMGXB4;GNAQ;ZNF703;RSPO3;RECK
Nucleocytoplasmic Transport (GO:0006913)	0.0155583	0.2289427	GLE1;AHCTF1;POM121;ATXN1;NUP210;ANP32A;RSRC1;NUP43;NUP58
Phospholipid Dephosphorylation (GO:0046839)	0.0155685	0.2289427	INPP5B;MTMR1;MTMR10;PLPPR4;INPP5K;SGPP1;MTM1
Protein Acylation (GO:0043543)	0.0155685	0.2289427	KAT2B;ZDHHC20;KAT6A;EP300;ZDHHC7;FOXO1;ZDHHC15
Regulation of Gluconeogenesis (GO:0006111)	0.0155685	0.2289427	SDHAF3;EP300;PPARGC1A;SIRT1;OGT;FOXO1;PTPN2
MyD88-dependent Toll-Like Receptor Signaling Pathway (GO:0002755)	0.0156847	0.2289855	IRAK1;TRAF6;REG3G;MAP3K7
pre-miRNA Processing (GO:0031054)	0.0156847	0.2289855	LIN28B;TUT4;AGO1;DICER1
Positive Regulation of Receptor Signaling Pathway via STAT (GO:1904894)	0.0158820	0.2302040	GHR;ERBB4;CCL5;CAMK2A;EP300;TTF2;NLK;CSF2RA
Response to Hydrogen Peroxide (GO:0042542)	0.0158820	0.2302040	PPIF;ADAM9;GLYAT;ECT2;SIRT1;FXN;RHOB;NFE2L2
Neuron Projection Guidance (GO:0097485)	0.0163538	0.2355444	EPHA5;NOTCH2;SEMA6A;DCC;SEMA6D;MYCBP2;SIAH1;SEMA3G;UNC5D;SEMA4G;KLF7;PALLD;FEZ2;CNTN1;NPTN;SLIT2;ARHGEF40;SOS1
Regulation of Translation (GO:0006417)	0.0163669	0.2355444	EIF4A2;MTPN;EIF4A3;STK39;FOXO3;BZW1;ELAVL1;TOB1;LARP1B;FXR1;SYNCRIP;PAIP2;ZNF540;IFRD2;RPL13A;ELP4;LARP4;WT1;CSDE1;CAPRIN1;HNRNPD;EIF3C;OGT;EIF4G2;METAP1
Regulation of Bone Mineralization (GO:0030500)	0.0166701	0.2389520	FBN2;CCR1;CYP27B1;KL;TENT5A;ATP2B1;ACVR2B;SLC8A1;GPM6B;ACVR2A

Positive Regulation of Intracellular Signal Transduction (GO:1902533)	0.0167219	0.2389520	THRB;WWC1;RORA;PTPN22;TNF;EDA2R;IGF1R;TRIM8;FGF7;GRM5;FLOT2;MAP3K7;CARD10;CHUK;PARP1;PRKCB;ZDHHC13;PRKCA;SOX11;OPRM1;F3;SIRT1;TGFBR1;TGFBR3;TRAF6;GFRAL;PELI1;SOS1;TP53;TSPYL5;HBEGF;ANKRD17;NFAT5;SLC20A1;TTK;GATA3;PIK3R1;TANK;RTN4;CYP27B1;BCL2L11;IRAK1;ERBB4;CCL5;TSPAN6;MIER1;FLNA;RICTOR;ECT2;P2RY12;OSBPL8;NTRK2;MAP3K3;XBP1;SIAH1;DAB2IP;PTPN11;PUM1;GDF5;ACVR2A;CCDC88A;EPGN;VAPA;TRIP6;HPSE;LAMTOR3;MYD88
Transcription Initiation at RNA Polymerase II Promoter (GO:0006367)	0.0170434	0.2413252	SMARCD1;KMT2A;TAF9;MED9;GTF2H1;GTF2F1;ARID1B;CTCFL;MED11;KAT2B;MED14;GDNF;MED20;EP300;ELOC;TP53;PPARGC1A
Positive Regulation of Vascular Endothelial Growth Factor Production (GO:0010575)	0.0170466	0.2413252	MIA3;RORA;HPSE;PTGS2;IL6ST;ISL1
Protein Modification by Small Protein Conjugation (GO:0032446)	0.0175215	0.2413252	HDAC4;ZNF451;RNF34;DCUN1D3;DCUN1D4;MYCBP2;UBE2D3;UBR1;DTX4;IFIH1;RNF135;VCPIP1;HERC3;SUMO1;FBXO4;ELOC;ATG7;FBXO9;BARD1;ATG3;RNF44;CRBN;BCL11A;USP9X;FBXW7;SIRT1;PIAS2;RNF168;CNOT4;ITCH;KLHL7;UBA3;MDM4;CBLL1;NFE2L2;CUL4B
Heart Trabecula Morphogenesis (GO:0061384)	0.0175715	0.2413252	DLL4;TGFBR3;NRG1;RBPJ;TGFBR1
Interleukin-1-Mediated Signaling Pathway (GO:0070498)	0.0175715	0.2413252	IRAK1;TRAF6;PLCB1;MAP3K7;MYD88
Phosphatidylinositol Dephosphorylation (GO:0046856)	0.0175715	0.2413252	INPP5B;MTMR1;MTMR10;INPP5K;MTM1
Positive Regulation of Cardiac Muscle Hypertrophy (GO:0010613)	0.0175715	0.2413252	MEF2A;MTPN;PARP1;PRKCA;IL6ST
Regulation of Cardiac Conduction (GO:1903779)	0.0175715	0.2413252	RYR2;TMEM65;PRKCA;ATP2B1;SLC8A1
Regulation of Cellular Response to Transforming Growth Factor Beta Stimulus (GO:1903844)	0.0175715	0.2413252	SMAD2;TGFBR3;ZNF703;SOX11;NREP
Regulation of Necroptotic Process (GO:0060544)	0.0175715	0.2413252	ITCH;PARP1;PELI1;SPATA2;OGT
Regulation of Nuclear-Transcribed mRNA Catabolic Process, Deadenylation-Dependent Decay (GO:1900151)	0.0175715	0.2413252	SYNCRIP;CSDE1;HNRNPD;RC3H1;TOB1
Response to Glucose (GO:0009749)	0.0176040	0.2413252	XBP1;KCNB1;ZBTB20;PRKCA;SELENOT;ZNF236;OGT;PAX2;IGF1R
T Cell Activation (GO:0042110)	0.0176928	0.2417223	CD84;CXADR;CD80;PTPN22;GATA3;PIK3R1;TANK;LFNG;DLL4;MDK;TNFSF4;GRB2;SOS1;DCAF12;PTPN2;NCK1
Cellular Response to Hydrogen Peroxide (GO:0070301)	0.0181665	0.2420642	PPIF;GLYAT;ECT2;SIRT1;FXN;RHOB;NFE2L2
Regulation of p38MAPK Cascade (GO:1900744)	0.0181665	0.2420642	DLG1;PHLPP1;DUSP10;STK39;DAB2IP;PTPN22;MAP3K4
mRNA Transcription (GO:0009299)	0.0181967	0.2420642	DDX5;THRB;CREB1;TAF9;ZBTB1;TP53;HIPK3;EREG
Negative Regulation of Organelle Assembly (GO:1902116)	0.0181967	0.2420642	YAP1;KAT2B;USP32;PHF23;FEZ2;CCP110;TRIM37;MTM1
Negative Regulation of Ubiquitin-Dependent Protein Catabolic Process (GO:2000059)	0.0181967	0.2420642	PHF20L1;USP14;UBXN1;USP38;USP9X;TAF9;OGT;METAP1
Neural Crest Cell Development (GO:0014032)	0.0181967	0.2420642	SEMA6A;GDNF;ERBB4;SEMA6D;SEMA3G;SNAI2;NRG1;SEMA4G
Positive Regulation of Myeloid Leukocyte Differentiation (GO:0002763)	0.0181967	0.2420642	CCR1;CSF1;TRAF6;PRKCA;TMEM64;TNF;RUNX1;PF4
Regulation of Dendrite Development (GO:0050773)	0.0181967	0.2420642	GSK3B;SMAD1;BCL11A;DCC;SDC2;DAB2IP;CAMSAP2;ZDHHC15
Calcium Ion Homeostasis (GO:0055074)	0.0182776	0.2423436	CCR1;RYR2;CALCB;STC1;ITPR3;ATP1B1;SLC8A1;TRDN;PKHD1;CYP27B1;STIM1;ASPH;CCL5;NPTN;SNCA
Positive Regulation of Intracellular Protein Transport (GO:0090316)	0.0191349	0.2528818	GSK3B;XBP1;ASPH;BAG3;ZIC1;UBR5;EP300;FLNA;PIK3R1;EFCAB7;ECT2
Negative Regulation of Response to External Stimulus (GO:0032102)	0.0194253	0.2548489	RB1;USP38;PARP1;CASK;RORA;MIA3;AHR;GATA3;DTX4;ISL1;FXR1;ITCH;MMRN1;RTN4RL1;SLC39A8;FAM76B;FEM1C;ENPP3;PTPN2;CERS2
Positive Regulation of DNA-binding Transcription Factor Activity (GO:0051091)	0.0196109	0.2548489	FOXA1;EPHA5;FZD1;FANK1;HDAC4;MTPN;CHUK;CAMK2A;RORA;TNF;TANK;TRIM8;IRAK1;GDNF;ZIC2;TRAF6;EP300;TRIM37;MAP3K13;PPARGC1A;MYD88;NEUROG2
Negative Regulation of Protein Polymerization (GO:0032272)	0.0202801	0.2548489	DYRK1A;TWF1;SLIT2;SSH2;SNCA;PFN2
Regulation of Extrinsic Apoptotic Signaling Pathway in Absence of Ligand (GO:2001239)	0.0202801	0.2548489	GDNF;EYA4;SNAI2;NRG1;TNF;PF4
Protein Phosphorylation (GO:0006468)	0.0205018	0.2548489	GSK3B;GMFB;DYRK2;CAB39;CAMK2A;STK39;NLK;IGF1R;SLK;IRAK1;ERBB4;MAP3K20;PDK3;ABL2;MAPK1;MAP3K8;MAP3K7;CDK17;MAP3K3;NTRK2;PRKCB;CSNK1A1;PRKCE;DYRK1A;PRKCA;BRAF;HIPK3;TGFBR1;UHMK1;MAPK10;SNRK;MELK;CREB1;ABI2;TAOK3;GDNF;PRKD3;CAMK4;STK17B;SGK3;MAP3K13
Cellular Response to Epinephrine Stimulus (GO:0071872)	0.0205126	0.2548489	RYR2;PRKCA;SNCA
Monocyte Activation (GO:0042117)	0.0205126	0.2548489	CSF1;ADAM9;FOXP1
Monoubiquitinated Protein Deubiquitination (GO:0035520)	0.0205126	0.2548489	USP47;USP32;USP9X
Negative Regulation of Glycogen Metabolic Process (GO:0070874)	0.0205126	0.2548489	GSK3B;STK39;INPP5K
Negative Regulation of Protein-Containing Complex Disassembly (GO:0043242)	0.0205126	0.2548489	RUBCN;PHF23;TNF
Positive Regulation of Epithelial Tube Formation (GO:1905278)	0.0205126	0.2548489	GDNF;SIX4;GATA3
Regulation of Epithelial Cell Apoptotic Process (GO:1904035)	0.0205126	0.2548489	PKHD1;YAP1;MDK
Regulation of Myeloid Leukocyte Differentiation (GO:0002761)	0.0205126	0.2548489	PRXL2A;CAMK4;FOXP1
Cell Communication by Electrical Coupling Involved in Cardiac Conduction (GO:0086064)	0.0206072	0.2548489	RYR2;PRKCA;ATP1B1;SLC8A1
Intracellular Potassium Ion Homeostasis (GO:0030007)	0.0206072	0.2548489	KCNMA1;ATP1A2;PRKCA;ATP1B1
miRNA Metabolic Process (GO:0010586)	0.0206072	0.2548489	DDX5;LIN28B;TUT4;DICER1
Negative Regulation of Cartilage Development (GO:0061037)	0.0206072	0.2548489	SNAI2;PTPN11;GDF5;TGFBR1
Positive Regulation of Acute Inflammatory Response (GO:0002675)	0.0206072	0.2548489	C2CD4A;PTGS2;IL6ST;TNF
Positive Regulation of Cardiac Muscle Cell Proliferation (GO:0060045)	0.0206072	0.2548489	TGFBR3;ERBB4;NRG1;RBPJ
Positive Regulation of Macrophage Chemotaxis (GO:0010759)	0.0206072	0.2548489	CSF1;MDK;CCL5;MAPK1
Regulation of Granulocyte Macrophage Colony-Stimulating Factor Production (GO:0032645)	0.0206072	0.2548489	CD84;CD80;IL17D;ISL1
Positive Regulation of Wound Healing (GO:0090303)	0.0207431	0.2550621	XBP1;OCLN;PRKCE;ENPP4;REG3G;TTF2;HPSE;HBEGF
post-Golgi Vesicle-Mediated Transport (GO:0006892)	0.0207506	0.2550621	SYS1;SORT1;VPS13C;SEC16A;RAB3IP;VPS13A;VPS54;EXOC5;STEAP2;EPS15
Negative Regulation of Epithelial to Mesenchymal Transition (GO:0010719)	0.0210543	0.2558539	FOXA1;GSK3B;SPRED1;HPN;DAB2IP;GATA3;VASN
Regulation of D-glucose Import (GO:0046324)	0.0210543	0.2558539	OSBPL8;OCLN;PTPN11;PIK3R1;SORBS1;RHOQ;APPL1
Regulation of Cytosolic Calcium Ion Concentration (GO:0051480)	0.0210543	0.2558539	RYR2;TRPC5;CALCB;ASPH;TTF2;ATP2B1;TMEM64
Positive Regulation of Growth (GO:0045927)	0.0211295	0.2558539	YAP1;MTPN;BRAT1;MAPKAP1;HPN;NRG1;RICTOR;PLCB1;FXN;TGFBR1;CDKN2AIP;EIF4G2
Negative Regulation of TOR Signaling (GO:0032007)	0.0211313	0.2558539	PREX2;MINAR1;GSK3B;UBE2W;DDIT4;PELI1;PRKCA;UBR1;DEPDC5;NLK;SIRT1
DNA Repair-Dependent Chromatin Remodeling (GO:0140861)	0.0215945	0.2560946	RNF168;UBR5;TRIP12;SIRT1;ASF1A
Inhibitory Synapse Assembly (GO:1904862)	0.0215945	0.2560946	GABRA1;GABRB2;GABRA5;GABRA4;GABRG1

Intrinsic Apoptotic Signaling Pathway in Response to DNA Damage by P53 Class Mediator (GO:0042771)	0.0215945	0.2560946	DYRK2;DDIT4;EP300;SIRT1;TP53
Myoblast Differentiation (GO:0045445)	0.0215945	0.2560946	RB1;DDX5;JAG1;NRG1;RBPJ
Negative Regulation of Signal Transduction by P53 Class Mediator (GO:1901797)	0.0215945	0.2560946	RNF34;DYRK1A;SNAI2;RFFL;SIRT1
Regulation of Transcription Regulatory Region DNA Binding (GO:2000677)	0.0215945	0.2560946	RB1;NSD1;TRAF6;SOX11;GATA3
Response to Gamma Radiation (GO:0010332)	0.0215945	0.2560946	YAP1;DCUN1D3;MAP3K20;TP53;TSPYL5
Response to Endoplasmic Reticulum Stress (GO:0034976)	0.0218000	0.2577759	GSK3B;PTPN1;JUN;XBP1;TOR1A;SEC16A;AMFR;FAF2;DAB2IP;PARP16;PIK3R1;TANK;HSP90B1;RNF145;SERP1;BCL2L11;TMEM33;DNAJC10;FBXO6;MAN1A1;TP53;FAM8A1;NFE2L2;NCK1
Cytoplasmic Microtubule Organization (GO:0031122)	0.0222713	0.2625815	CEP126;CCDC88A;DLG1;SLK;CCDC13;HOOK1;HOOK3;TUBGCP4;TRDN
Regulation of Anatomical Structure Morphogenesis (GO:0022603)	0.0226999	0.2668564	GSK3B;ZRANB1;SDC2;RC3H1;LARP4;ZDHHC15;ARHGAP44;LFNG;GDNF;ADGRB3;NF1;PHIP;PDLIM5;PDZD8;ZNF135;LZTS3;PPFIA2
Positive Regulation of Translation (GO:0045727)	0.0230774	0.2705076	FASTKD2;EIF4A3;STK39;LARP4;ELAVL1;LARP1B;UHMK1;FXR1;SYNCRIP;BOLL;CSDE1;CCL5;HNRNPD;EIF3C;OGT
Protein Polyubiquitination (GO:0000209)	0.0232704	0.2712701	RNF34;UBE3C;MYCBP2;FBXO28;UBE2D3;DTX4;TANK;ANAPC10;TRIM8;RNF135;TRIM2;UBR5;FBXO4;RFFL;FBXO9;BARD1;KCMF1;AMFR;RC3H1;UBE2A;RNF168;UBE2W;ITCH;KBTBD6;TRAF6;PELI1;TRIP12
Response to Ionizing Radiation (GO:0010212)	0.0232766	0.2712701	RNF168;BARD1;BRAT1;DCUN1D3;GRB2;ECT2;TNF;SIRT1;TP53;TANK;RHOB
Camera-Type Eye Development (GO:0043010)	0.0239054	0.2754235	FBN2;NEUROD4;WT1;NF1;SOX11;PAX2
Cytoplasmic Pattern Recognition Receptor Signaling Pathway (GO:0002753)	0.0239054	0.2754235	IFIH1;RNF135;ITCH;RNF34;TRAF6;MAP3K7
mRNA Splice Site Recognition (GO:0006376)	0.0239054	0.2754235	SF3A1;CELF2;PSIP1;SRSF6;LUC7L2;SRSF12
Regulation of Vascular Endothelial Growth Factor Production (GO:0010574)	0.0239054	0.2754235	MIA3;RORA;HPSE;PTGS2;IL6ST;ISL1
Negative Regulation of Endothelial Cell Migration (GO:0010596)	0.0242460	0.2785538	DLL4;MECP2;DAB2IP;STC1;SLIT2;AGTR2;TNF
DNA Damage Response (GO:0006974)	0.0244771	0.2804125	YAP1;NFAT5;DYRK2;USP32;BRAT1;TAF9;ZBTB1;TTF2;PDS5B;PDS5A;TANK;FOXO1;CDKN2AIP;ZBTB40;CASP9;VCPIP1;BCL2L11;SUMO1;UBR5;FBXO6;POLK;ATRIP;BARD1;WDHD1;USP47;CBX5;PARP1;FBXW7;CCDC13;NFATC2;GTF2H1;CHD1L;UBE2A;GNL1;SIRT1;FOXP1;RNF168;FAM111A;TAOK3;GDNF;GRB2;TRIP12;TP53;ASF1A;CUL4B
Cellular Response to BMP Stimulus (GO:0071773)	0.0249101	0.2837652	TGFBR3;SMAD1;DDX5;USP9X;USP9Y;ACVR2B;GDF5;SMAD5;ACVR2A
Gonad Development (GO:0008406)	0.0249101	0.2837652	KITLG;SALL1;SIX4;SRD5A2;WT1;CSDE1;NCOA4;GATA3;LRP2
Positive Regulation of Cell Cycle Process (GO:0090068)	0.0255480	0.2896654	PRKCE;KIF14;CDC14A;NCAPH;EREG;BTC;EPGN;SPAST;DBF4;RAD21;MAP3K20;PHIP;PKP4;ECT2;ZNF367;E2F7
Endocardial Cushion Development (GO:0003197)	0.0261713	0.2896654	SMAD2;ADAMTS5;MDM4;DKK1;ISL1
Negative Regulation of Cell Development (GO:0010721)	0.0261713	0.2896654	GSK3B;PAX6;HOOK3;IL17D;TNF
Peptidyl-Tyrosine Dephosphorylation (GO:0035335)	0.0261713	0.2896654	PTPN1;PTPN11;PTPN12;PTPN2;TNS2
Post-Transcriptional Gene Silencing (GO:0016441)	0.0261713	0.2896654	COLEC12;HELZ;DICER1;ELOC;PUM1
Negative Regulation of Axon Extension (GO:0030517)	0.0263769	0.2896654	SEMA6D;SEMA3G;DIP2B;RTN4
Negative Regulation of Chondrocyte Differentiation (GO:0032331)	0.0263769	0.2896654	SNAI2;PTPN11;GDF5;TGFBR1
Nucleotide-Binding Domain, Leucine Rich Repeat Containing Receptor Signaling Pathway (GO:0035872)	0.0263769	0.2896654	LACC1;ITCH;RNF34;MAP3K7
Regulation of IRE1-mediated Unfolded Protein Response (GO:1903894)	0.0263769	0.2896654	PTPN1;BCL2L11;TMEM33;DAB2IP
Regulation of MHC Class II Biosynthetic Process (GO:0045346)	0.0263769	0.2896654	HDAC2;XBP1;SIRT1;PF4
Stress-Activated MAPK Cascade (GO:0051403)	0.0263769	0.2896654	MAP3K20;MAPK1;MAP3K13;MAP3K7
Sympathetic Nervous System Development (GO:0048485)	0.0263769	0.2896654	GDNF;NF1;SOX11;GATA3
Development of Primary Male Sexual Characteristics (GO:0046546)	0.0265741	0.2896654	KITLG;SIX4;SRD5A2;WT1;CSDE1;NCOA4;GATA3;LRP2
Male Gonad Development (GO:0008584)	0.0265741	0.2896654	KITLG;SIX4;SRD5A2;WT1;CSDE1;NCOA4;GATA3;LRP2
Negative Regulation of Proteasomal Protein Catabolic Process (GO:1901799)	0.0265741	0.2896654	PHF20L1;USP14;UBXN1;USP38;USP9X;TAF9;OGT;METAP1
Neurotransmitter Transport (GO:0006836)	0.0265741	0.2896654	SLC38A1;UNC13A;SLC6A15;SLC1A1;SLC1A3;SLC29A1;SLC38A2;SYN2
Protein O-linked Glycosylation (GO:0006493)	0.0267914	0.2912490	GALNT18;GALNT1;TET2;TET1;TMTC1;GALNT10;POFUT2;POFUT1;FUT9;B3GNT5;POC1B-GALNT4;OGT;FKTN
Metal Ion Transport (GO:0030001)	0.0276893	0.3002034	CCR1;RYR2;TRPC5;KCNH5;CALCRL;CACNA2D1;SLC41A2;CAMK2A;KCNJ16;ATP1A2;KCNA6;LRP2;SLC4A4;CACNB1;CYP27B1;KCNV1;CACNB4;CCL5;KCNMA1;CLDN16;SCN3A;SCN1A
Hemopoiesis (GO:0030097)	0.0279377	0.3005031	NOTCH2;JAG1;KMT2A;TET2;RUNX1;TGFBR3;KITLG;SNRK;MELK;KAT6A;CDK13;TP53;OGT;SH2B3
L-amino Acid Transport (GO:0015807)	0.0279399	0.3005031	SLC38A1;SLC7A8;SLC6A15;SLC1A1;SLC1A3;SLC38A2
Positive Regulation of Response to Wounding (GO:1903036)	0.0279399	0.3005031	CNTF;OCLN;MDK;PRKCE;REG3G;HBEGF
Positive Regulation of TOR Signaling (GO:0032008)	0.0280365	0.3007420	XBP1;USP32;CSNK1A1;USP9X;CCL5;EP300;RICTOR;F3;GPR155;LAMTOR3;OGT
Positive Regulation of Cellular Component Organization (GO:0051130)	0.0287662	0.3013090	RB1;SH3GLB1;GSK3B;FRMPD4;DAB2IP;BRK1;NRG1;TNF;TGFBR1;IGF1R;RUNX1;BCL2L11;SUMO1;CBLL1;SNCA
Regulation of Cell Growth (GO:0001558)	0.0289006	0.3013090	RB1;YAP1;MTPN;DCUN1D3;MAPKAP1;BRAT1;HPN;KIF14;CDKN2AIP;RTN4;RERG;CYP27B1;RICTOR;SLIT2;MINAR1;XBP1;NRG1;TGFBR1;ST7L;ITCH;WT1;SGK3;AGTR2;TP53;FXN;EIF4G2
RISC Complex Assembly (GO:0070922)	0.0289834	0.3013090	COLEC12;AGO1;DICER1
Axon Regeneration (GO:0031103)	0.0289834	0.3013090	RTN4RL1;MTR;NREP
Insulin Metabolic Process (GO:1901142)	0.0289834	0.3013090	PCSK2;ERO1B;YIPF5
Negative Regulation of Cytoplasmic Translation (GO:2000766)	0.0289834	0.3013090	PARP16;NMNAT2;CPEB4
Negative Regulation of Potassium Ion Transmembrane Transporter Activity (GO:1901017)	0.0289834	0.3013090	CAB39;SUMO1;STK39
Negative Regulation of Striated Muscle Cell Apoptotic Process (GO:0010664)	0.0289834	0.3013090	MDK;PPP1R10;NRG1
Neuron Projection Regeneration (GO:0031102)	0.0289834	0.3013090	RTN4RL1;MTR;NREP
Peptidyl-Lysine Acetylation (GO:0018394)	0.0289834	0.3013090	KAT2B;EP300;SIRT1
Regulation of Cardiac Muscle Cell Differentiation (GO:2000725)	0.0289834	0.3013090	FRS2;SOX6;DKK1
Regulation of Translation in Response to Endoplasmic Reticulum Stress (GO:0036490)	0.0289834	0.3013090	DNAJC3;MAP3K20;NCK1
Negative Regulation of Defense Response (GO:0031348)	0.0293310	0.3041402	RB1;USP38;PARP1;RORA;MIA3;AHR;GATA3;DTX4;ISL1;FXR1;ITCH;SLC39A8;FAM76B;FEM1C;ENPP3;PTPN2

Regulation of Endothelial Cell Apoptotic Process (GO:2000351)	0.0298787	0.3090270	XBP1;FGG;NDNF;GATA3;TNF;F3;ICAM1;NFE2L2
Generation of Neurons (GO:0048699)	0.0304544	0.3141783	FZD1;MTPN;NTRK2;MYEF2;DCC;USP9X;DCDC2;ACSL4;SOX11;NDNF;ACSL3;PEX13;RTN4;RUNX1;PTPRD;SIX4;ABI2;FUT9;ERBB4;CASP3;DDIT4;WNT3;ASTN1
Cellular Response to Lipid (GO:0071396)	0.0311396	0.3190696	GSK3B;CD80;RORA;ATP1A2;AHR;NR3C1;PPM1E;CASP7;ZNF703;PDK3;ABL2;PF4V1;WNT3;XBP1;NCOA4;DAB2IP;IL36G;PPBP;PAX2;CREB1;TNFSF4;TRAF6;ADAM9;CCL28;MYD88;PF4;CHMP5
Regulation of Cytokine Production (GO:0001817)	0.0312365	0.3190696	CADM1;PPP1R11;MOG;TRIL;ZBTB26;ZBTB1;ZBTB20;GATA3;ATP2B1;DTX4;TNF;EREG;FOXP1;ZBTB2;IFIH1;HIC2;TNFSF4;CASP3;NPTN;CCN4;SNAI2
Gamma-Aminobutyric Acid Signaling Pathway (GO:0007214)	0.0313231	0.3190696	GABRA1;GABRB2;GABRA5;GABRA4;GABRG1
poly(A)+ mRNA Export From Nucleus (GO:0016973)	0.0313231	0.3190696	GLE1;NXF1;HHEX;NXT1;NXT2
Positive Regulation of T Cell Cytokine Production (GO:0002726)	0.0313231	0.3190696	DENND1B;TNFSF4;TRAF6;TANK;MAP3K7
+ Reg of Phosphatidylinositol 3-Kinase/Prot Kinase B Signal Transduction (GO:0051897)	0.0315126	0.3201936	P2RY12;OSBPL8;NTRK2;XBP1;PRKCA;PTPN11;GATA3;TNF;SIRT1;RTN4;TGFBR1;IGF1R;CCDC88A;CCL5;GFRAL;RICTOR;HPSE;SOS1;TSPYL5;HBEGF
Protein Localization to Plasma Membrane (GO:0072659)	0.0316471	0.3202122	FYB1;KCNB1;SEC16A;TTC7B;ZDHHC7;WDR72;ANK3;ATP1B1;TNF;HSP90B1;CCDC88A;EFR3A;DLG1;FCHO2;INPP5K;FLOT2;FLNA
Regulation of Cell Adhesion (GO:0030155)	0.0317422	0.3202122	KIF14;C2CD4A;PRKCA;MIA3;DUSP28;NRG1;LAMB1;PTPN11;TNF;PKHD1;MDK;CCL5;ABL2;PKP4;PLXNC1;TGFBI;CYTIP;CBLL1
Mesenchymal Cell Migration (GO:0090497)	0.0323987	0.3202122	SEMA6A;GDNF;ERBB4;SEMA6D;SEMA3G;SEMA4G
Positive Regulation of Signaling (GO:0023056)	0.0323987	0.3202122	CD80;SOX11;GATA3;TNF;CDKN2AIP;SNCA
Response to Epidermal Growth Factor (GO:0070849)	0.0323987	0.3202122	DAB2IP;INPP5K;MAPK1;SNAI2;PTPN11;PTPN12
Vascular Transport (GO:0010232)	0.0325272	0.3202122	SLC38A1;SLC22A5;SLC7A8;SLC1A1;SLC16A7;SLC1A3;ATP1A2;LRP2;SLC6A1;SLC29A1;SLC4A4;SLC38A2
Golgi to Vacuole Transport (GO:0006896)	0.0330189	0.3202122	SORT1;AP1G1;VPS54;RBSN
Autonomic Nervous System Development (GO:0048483)	0.0330189	0.3202122	GDNF;NF1;SOX11;GATA3
Cellular Response to Alkaloid (GO:0071312)	0.0330189	0.3202122	RYR2;CASP7;CASP3;SLC8A1
Cellular Response to Gamma Radiation (GO:0071480)	0.0330189	0.3202122	YAP1;MAP3K20;TP53;TSPYL5
Lipopolysaccharide-Mediated Signaling Pathway (GO:0031663)	0.0330189	0.3202122	IRAK1;PRKCE;TRAF6;PTPN22
Mesenchyme Morphogenesis (GO:0072132)	0.0330189	0.3202122	SMAD2;ADAMTS5;MDM4;ISL1
Negative Regulation of mRNA Processing (GO:0050686)	0.0330189	0.3202122	BARD1;RBM20;SRSF6;SRSF12
Negative Regulation of Signaling Receptor Activity (GO:2000272)	0.0330189	0.3202122	TSG101;CLEC12B;TNF;SNX6
Neuron Fate Commitment (GO:0048663)	0.0330189	0.3202122	DLL4;PAX6;ISL1;TGFBR1
Neurotrophin Signaling Pathway (GO:0038179)	0.0330189	0.3202122	KIDINS220;SORT1;CASP3;DDIT4
Presynapse Assembly (GO:0099054)	0.0330189	0.3202122	FZD1;NLGN4Y;PTPRD;PCLO
Regulation of Macrophage Differentiation (GO:0045649)	0.0330189	0.3202122	CSF1;PRKCA;PTPN2;PF4
Regulation of Response to Cytokine Stimulus (GO:0060759)	0.0330189	0.3202122	IFIH1;IRAK1;TAF9;SH2B3
Replication Fork Processing (GO:0031297)	0.0334549	0.3234563	BARD1;EXD2;FAM111A;MMS22L;FANCM;PARP1;POLK;ASF1A
Cellular Response to Molecule of Bacterial Origin (GO:0071219)	0.0335134	0.3234563	XBP1;CD80;DAB2IP;IL36G;AHR;PPBP;PPM1E;CASP7;TNFSF4;TRAF6;ADAM9;CCL28;MYD88;PF4V1;CHMP5;PF4
Heart Morphogenesis (GO:0003007)	0.0341425	0.3287443	TGFBR3;SMAD2;RYR2;ADAMTS5;OLFM1;RBM20;MDM4;ISL1;TGFBR1
Negative Regulation of Cellular Process (GO:0048523)	0.0345977	0.3323356	RB1;WWC1;WWC2;MIA3;TNF;RERG;MDK;PLXNC1;ARID2;TNS2;MINAR1;SS18;OPRM1;SIRT1;PAX2;EREG;RHOB;ST7L;KAT2B;SFRP4;THAP5;RBL1;AGTR2;CBLL1;TP53;SMARCD1;DCUN1D3;GATA3;PDS5B;TOB1;CDKN2AIP;RTN4;DLL4;CYP27B1;CLMN;ERBB4;NACC2;SLIT2;SH2B3;ZBTB7C;FKTN;BARD1;SMAD2;DAB2IP;SOD2;PTPN14;NR4A1;FABP3;WT1;STRN;MDM4;ZNF777;TGFBI;PTPN2
Branching Morphogenesis of an Epithelial Tube (GO:0048754)	0.0357682	0.3407322	DLL4;PKHD1;SALL1;GDNF;WT1;SLIT2;PAX2
Fibroblast Growth Factor Receptor Signaling Pathway (GO:0008543)	0.0357682	0.3407322	KL;FGF7;CHURC1;FGF14;FRS2;PTPN11;SHCBP1
Positive Regulation of Cytokinesis (GO:0032467)	0.0357682	0.3407322	SPAST;PRKCE;KIF14;PKP4;ECT2;ZNF367;CDC14A
Regulation of Canonical Wnt Signaling Pathway (GO:0060828)	0.0358089	0.3407322	AMER2;GSK3B;PRDM15;SCEL;HHEX;MDK;ZNF703;UBR5;RSPO3;SCYL2;USP47;CSNK1A1;SIAH2;AMFR;FZD6;SOX13;DAB2IP;TMEM64;DKK1;SFRP4;TBL1XR1;GNAQ;SNAI2;RECK;CSNK1A1L
Endocrine System Development (GO:0035270)	0.0370665	0.3452758	SALL1;MDK;WT1;NF1;TGFBR1
Metanephros Development (GO:0001656)	0.0370665	0.3452758	GDNF;SIX4;WT1;NF1;PAX2
Negative Regulation of Extrinsic Apoptotic Signaling Pathway via Death Domain Receptors (GO:1902042)	0.0370665	0.3452758	GSK3B;RNF34;FGG;RFFL;ICAM1
Negative Regulation of Response to Wounding (GO:1903035)	0.0370665	0.3452758	MMRN1;MDK;RTN4RL1;CASK;CERS2
Potassium Ion Homeostasis (GO:0055075)	0.0370665	0.3452758	KCNMA1;ATP1A2;PRKCA;ATP1B1;SLC12A6
Response to Ketone (GO:1901654)	0.0370665	0.3452758	YAP1;GHR;TGFBR3;GNAQ;SLIT2
TOR Signaling (GO:0031929)	0.0372949	0.3452758	CCDC88A;RPS6KA6;MAPKAP1;RICTOR;GATA3;LAMTOR3
Negative Regulation of Fat Cell Differentiation (GO:0045599)	0.0372949	0.3452758	YAP1;RORA;TNF;SIRT1;FOXO1;RUNX1T1
Regulation of Extrinsic Apoptotic Signaling Pathway via Death Domain Receptors (GO:1902041)	0.0372949	0.3452758	GSK3B;RNF34;FEM1B;FGG;RFFL;ICAM1
Regulation of Lamellipodium Assembly (GO:0010591)	0.0372949	0.3452758	AKIRIN1;OCLN;BRK1;TWF1;PIK3R1;SLIT2
Regulation of Postsynapse Organization (GO:0099175)	0.0372949	0.3452758	ARHGAP44;ABI2;PDLIM5;LZTS3;PPFIA2;ZDHHC15
Regulation of Wound Healing (GO:0061041)	0.0373109	0.3452758	OCLN;MMRN1;PRKCE;CASK;TTF2;REG3G;SRSF6;HBEGF
Regulation of Canonical NF-kappaB Signal Transduction (GO:0043122)	0.0375645	0.3455536	ANKRD17;NFAT5;SLC20A1;USP32;RORA;TANK;TNF;EDA2R;TRIM8;IRAK1;TSPAN6;MIER1;FLNA;FLOT2;SLC39A8;ECT2;MAP3K7;MAP3K3;CARD10;PARP1;CHUK;PRKCB;DAB2IP;ZDHHC13;SIRT1;TGFBR3;VAPA;TRAF6;PELI1;MYD88
Cellular Response to Unfolded Protein (GO:0034620)	0.0376827	0.3455536	PTPN1;XBP1;SERP1;BAG3;HSPB8;AMFR;DAB2IP;PARP16;NFE2L2
Muscle Organ Development (GO:0007517)	0.0376827	0.3455536	MEF2A;FXR1;ZBTB42;NEB;SOX6;UTRN;MEF2D;HBEGF;FKTN
Regulation of Calcium Ion Transport (GO:0051924)	0.0376827	0.3455536	CCR1;CD84;STIM1;CACNA2D1;CCL5;CAMK2A;STC1;INPP5K;ATP2B1
Transport Across Blood-Brain Barrier (GO:0150104)	0.0381319	0.3459862	SLC38A1;SLC22A5;SLC7A8;SLC1A1;SLC16A7;SLC1A3;ATP1A2;LRP2;SLC6A1;SLC29A1;SLC4A4;SLC38A2
Negative Regulation of Cell Population Proliferation (GO:0008285)	0.0383919	0.3459862	WWC1;HPN;WWC2;GATA3;PDS5B;TOB1;RERG;DLL4;CYP27B1;DUSP10;CLMN;ERBB4;NACC2;ARID2;PPARGC1A;SH2B3;TNS2;ZBTB7C;FKTN;MINAR1;KDM2B;DAB2IP;SOX11;OPRM1;SOD2;GDF5;PTPN14;MTSS1;EREG;TGFBR3;SFRP4;KAT2B;FABP3;WT1;NF1;STRN;MDM4;ZNF777;TP53;PTPN2
Golgi to Lysosome Transport (GO:0090160)	0.0390132	0.3459862	AP1G1;SORT1;RBSN

TRIF-dependent Toll-Like Receptor Signaling Pathway (GO:0035666)	0.0390132	0.3459862	TRAF6;CD300LF;MAP3K7
Angiogenesis Involved in Wound Healing (GO:0060055)	0.0390132	0.3459862	NDNF;PRCP;HPSE
Copper Ion Transport (GO:0006825)	0.0390132	0.3459862	SLC31A2;ATP7A;STEAP2
Metanephric Mesenchyme Development (GO:0072075)	0.0390132	0.3459862	SIX4;WT1;PAX2
miRNA Catabolic Process (GO:0010587)	0.0390132	0.3459862	LIN28B;TUT4;ELOC
Peptidyl-Threonine Dephosphorylation (GO:0035970)	0.0390132	0.3459862	PPP2CB;PPM1E;PPM1G
Positive Regulation of Chondrocyte Differentiation (GO:0032332)	0.0390132	0.3459862	SOX6;GDF5;SOX5
Positive Regulation of Hemostasis (GO:1900048)	0.0390132	0.3459862	ENPP4;HPSE;TTF2
Regulation of Macrophage Migration (GO:1905521)	0.0390132	0.3459862	P2RY12;CSF1;RTN4
Response to Sterol (GO:0036314)	0.0390132	0.3459862	SMAD2;RORA;TGFBR1
Synaptic Signaling (GO:0099536)	0.0390132	0.3459862	KCMF1;DTNA;UTRN
Ubiquitin-Dependent Protein Catabolic Process via the C-end Degron Rule Pathway (GO:0140627)	0.0390132	0.3459862	FEM1B;FEM1C;DCAF12
Cellular Response to UV (GO:0034644)	0.0395812	0.3484062	CASP9;MFAP4;PARP1;FBXW7;ZBTB1;MAP3K20;EP300;POLK;SIRT1;TP53;CUL4B
Negative Regulation of Wnt Signaling Pathway (GO:0030178)	0.0402399	0.3484062	GSK3B;AMER2;CSNK1A1;SIAH2;AMFR;FZD6;SOX13;DAB2IP;TMEM64;NLK;DKK1;CXXC4;SFRP4;HMGXB4;MDK;SNAI2;SCYL2;CSNK1A1L
Negative Regulation of Protein Metabolic Process (GO:0051248)	0.0402399	0.3484062	ZNF540;CD84;IFRD2;EIF4A3;DAB2IP;RPL13A;INHBB;TTF2;TOB1;AZIN1;PHF20L1;SYNCRIP;WT1;CAPRIN1;FLOT2;FLNA;MDM4;PAIP2
Monoatomic Cation Transport (GO:0006812)	0.0402936	0.3484062	CALHM1;GRIN2A;ATP10D;ATP1A2;LRP2;CLDN16;SLC39A1
G Protein-Coupled Acetylcholine Receptor Signaling Pathway (GO:0007213)	0.0405473	0.3484062	GNAQ;HRH4;OPRM1;PLCB1
Electron Transport Chain (GO:0022900)	0.0405473	0.3484062	ENOX2;VDAC2;HCCS;PPARGC1A
Negative Regulation of Intracellular Protein Transport (GO:0090317)	0.0405473	0.3484062	BARD1;SUMO1;YWHAB;FAM76B
Peroxisomal Membrane Transport (GO:0015919)	0.0405473	0.3484062	PEX16;ABCD3;TRIM37;RAB8B
Positive Regulation of Cardiac Muscle Tissue Growth (GO:0055023)	0.0405473	0.3484062	TGFBR3;ERBB4;NRG1;RBPJ
Positive Regulation of Cartilage Development (GO:0061036)	0.0405473	0.3484062	MDK;SOX6;GDF5;SOX5
Positive Regulation of Cholesterol Metabolic Process (GO:0090205)	0.0405473	0.3484062	STARD4;MAPK1;SCAP;PRKCA
Regulation of D-glucose Transmembrane Transport (GO:0010827)	0.0405473	0.3484062	OCLN;PRKCB;INPP5K;BRAF
Regulation of Cartilage Development (GO:0061035)	0.0405473	0.3484062	MDK;TRPS1;SOX6;SOX5
Stress-Activated Protein Kinase Signaling Cascade (GO:0031098)	0.0405473	0.3484062	MAP3K20;MAPK1;MAP3K13;MAP3K7
Calcium Ion Transport (GO:0006816)	0.0405811	0.3484062	CCR1;RYR2;TRPC5;MCOLN3;CALCRL;CACNA2D1;CAMK2A;CACNA1A;SLC8A1;CACNB1;CYP27B1;GRIN2A;CACNB4;STIM1;ASPH;CCL5
Positive Regulation of Type I Interferon Production (GO:0032481)	0.0407507	0.3484062	IFIH1;RNF135;POLR3A;CHUK;IRAK1;G3BP1;PTPN22;PTPN11;TANK;MYD88
Regulation of Cell Communication (GO:0010646)	0.0407507	0.3484062	RYR2;PTPN1;DBNDD2;CXADR;ASPH;PPP6R3;STRN;CDK13;SLC8A1;TRDN
Negative Regulation of Protein-Containing Complex Assembly (GO:0031333)	0.0414650	0.3537651	GSK3B;CSNK1A1;LAMP2;KIF14;EP300;RPL13A;DKK1;ISL1;OGT
Epithelial Tube Morphogenesis (GO:0060562)	0.0426393	0.3612460	PKHD1;RYR2;GATA3;SLIT2;MTSS1;RHOB
Negative Regulation of JNK Cascade (GO:0046329)	0.0426393	0.3612460	ITCH;DUSP10;TAOK3;PTPN22;HIPK3;FKTN
Positive Regulation of Cell Growth (GO:0030307)	0.0428528	0.3612460	YAP1;MTPN;UNC13A;BCL11A;MAPKAP1;BRAT1;HPN;NRG1;CDKN2AIP;TGFBR1;RICTOR;MAP3K13;FXN;EIF4G2
Negative Regulation of Canonical Wnt Signaling Pathway (GO:0090090)	0.0431399	0.3612460	GSK3B;AMER2;CSNK1A1;SIAH2;AMFR;FZD6;SOX13;DAB2IP;TMEM64;DKK1;SFRP4;MDK;SNAI2;SCYL2;CSNK1A1L
Activin Receptor Signaling Pathway (GO:0032924)	0.0434138	0.3612460	SMAD2;INHBB;ACVR2B;ACVR2A;TGFBR1
Negative Regulation of Axonogenesis (GO:0050771)	0.0434138	0.3612460	DCC;SEMA6D;SEMA3G;DIP2B;RTN4
Positive Regulation of Cilium Assembly (GO:0045724)	0.0434138	0.3612460	GSK3B;CCDC88A;WRAP73;RAB3IP;CCP110
Regulation of Cardiac Muscle Hypertrophy (GO:0010611)	0.0434138	0.3612460	MEF2A;MTPN;PARP1;PRKCA;IL6ST
Regulation of Cell Junction Assembly (GO:1901888)	0.0434138	0.3612460	SETD5;SNAI2;PRKCA;PDLIM5;SRGAP2B
Regulation of Centrosome Cycle (GO:0046605)	0.0434138	0.3612460	PKHD1;PDCD6IP;SIRT1;RNF103-CHMP3;CHMP5
Response to Glucocorticoid (GO:0051384)	0.0434138	0.3612460	BCL2L11;SLIT2;NR3C1;TNF;ISL1
Stress Granule Assembly (GO:0034063)	0.0434138	0.3612460	DAZAP2;CSDE1;G3BP1;G3BP2;C9ORF72
Phosphatidylinositol Metabolic Process (GO:0046488)	0.0444144	0.3680577	INPP4A;INPP5B;MTMR1;MTMR10;INPP5D;INPP5K;TNFAIP8L3;PIP5K1B;PLCB1;MTM1
Regulation of Translational Initiation (GO:0006446)	0.0444144	0.3680577	EIF4A2;DNAJC3;BOLL;CSDE1;CCL5;PAIP2;BZW1;C8ORF88;EIF4G2;UHMK1
Notch Signaling Pathway (GO:0007219)	0.0451759	0.3681479	NOTCH2;DLL4;JAG1;POFUT1;SNAI2;DTX4;RBPJ
Membrane Fission (GO:0090148)	0.0451759	0.3681479	SH3GLB1;TSG101;SPAST;STAM;EXOC5;RNF103-CHMP3;CHMP5
Transcription by RNA Polymerase II (GO:0006366)	0.0453620	0.3681479	NFAT5;XBP1;CCNT2;THRB;PARP1;ICE2;TAF9;ZBTB1;GTF2H1;ETV1;PAX5;ELP4;TTF2;GTF2F1;CREB1;XRN2;MED20;ELOC;RPRD1A;PKNOX1;PPARGC1A;POLR2K;SOX5
Neuron Migration (GO:0001764)	0.0454950	0.3681479	NTRK2;ABI2;DCC;USP9X;DDIT4;DCDC2;NDNF;PEX13;ASTN1
Post-Transcriptional Regulation of Gene Expression (GO:0010608)	0.0454950	0.3681479	MTPN;RC3H1;ELP4;FOXO3;LARP4;MEX3D;PUM1;SPOUT1;METAP1
			NOTCH2;FASTKD2;BRAT1;AHR;FOXO1;RTN4;FXR1;CASP9;CASP7;BCL2L11;CASP3;EP300;IFT57;MAP3K7;TRPC5;SEMA6A;PARP1;FEM1B;PRKCE;SIAH1;DAB2IP;RHOB;MELK;D
Apoptotic Process (GO:0006915)	0.0458870	0.3681479	DIT4;OGT;MYD88
Epithelial to Mesenchymal Transition (GO:0001837)	0.0458915	0.3681479	TGFBR3;GSK3B;OLFM1;DDX5;SNAI2;RBPJ;SOS1;TGFBR1
Macromolecule Glycosylation (GO:0043413)	0.0458915	0.3681479	GMPPB;TMEM59;SERP1;FUT9;B3GNT5;ST8SIA3;ST8SIA4;FKTN
Negative Regulation of Cell Projection Organization (GO:0031345)	0.0458915	0.3681479	MINAR1;HDAC2;DCC;RTN4RL1;BCL11A;DPYSL3;DENND5A;ZNF365
Positive Regulation of Actin Filament Bundle Assembly (GO:0032233)	0.0458915	0.3681479	CCDC88A;SYNPO2;CCN2;PPM1E;MTSS1;TGFBR1;AMOT;PFN2
Positive Regulation of Protein Modification by Small Protein Conjugation or Removal (GO:1903322)	0.0464417	0.3681479	HDAC4;GSK3B;FBXW7;PHF23;TRAF6;DCUN1D3;DCUN1D4;MYCBP2;FBXO4;TANK;TSPYL5
Regulation of Transcription Elongation by RNA Polymerase II (GO:0034243)	0.0464417	0.3681479	RNF168;MED11;MED14;CCNT2;PARP1;MED20;INTS2;PWWP2A;MED9;CDK13;WDR43
Amide Biosynthetic Process (GO:0043604)	0.0482950	0.3681479	SLC25A15;PANK3;CERS6;GGT6;SPTLC2;VAPA;MYCBP2;GCLM;CERS2;GNE
JNK Cascade (GO:0007254)	0.0484402	0.3681479	MAPK10;FGF14;MAP3K20;MAP3K13;MAP3K7;DUSP9

Cellular Response to Osmotic Stress (GO:0071470)	0.0484402	0.3681479	NFAT5;CAB39;STK39;LRRC8D;NLK;SLC12A6
Negative Regulation of Blood Vessel Endothelial Cell Migration (GO:0043537)	0.0484402	0.3681479	DLL4;MECP2;CARD10;MMRN1;AGTR2;TNF
Regulation of DNA Damage Response, Signal Transduction by P53 Class Mediator (GO:0043516)	0.0484402	0.3681479	DDX5;SPRED1;DYRK1A;SNAI2;KMT5A;SIRT1
Regulation of Developmental Growth (GO:0048638)	0.0484402	0.3681479	GHR;GSK3B;OLFM1;SIX4;MYCBP2;PLCB1
Regulation of Proteasomal Ubiquitin-Dependent Protein Catabolic Process (GO:0032434)	0.0488821	0.3681479	GSK3B;UBXN1;USP38;DESI2;CSNK1A1;USP9X;TAF9;SIRT1;PHF20L1;HECTD1;SUMO1;GNA12;OGT;METAP1
Branching Involved in Ureteric Bud Morphogenesis (GO:0001658)	0.0489661	0.3681479	SALL1;GDNF;WT1;PAX2
Detection of Mechanical Stimulus Involved in Sensory Perception (GO:0050974)	0.0489661	0.3681479	COL11A1;HPN;KCNK2;SCN1A
Intracellular Lipid Transport (GO:0032365)	0.0489661	0.3681479	FABP3;ABCD3;TMEM41B;SGPP1
Negative Regulation of Insulin Secretion (GO:0046676)	0.0489661	0.3681479	KLF7;KCNB1;INHBB;FOXO1
Negative Regulation of Nucleocytoplasmic Transport (GO:0046823)	0.0489661	0.3681479	BARD1;SUMO1;YWHAB;FAM76B
Pharyngeal System Development (GO:0060037)	0.0489661	0.3681479	SIX4;GATA3;ISL1;TGFBR1
Positive Regulation of Glucose Metabolic Process (GO:0010907)	0.0489661	0.3681479	SIRT1;PPARGC1A;FOXO1;PTPN2
Protein Branched Polyubiquitination (GO:0141198)	0.0489661	0.3681479	ITCH;TRAF6;UBR5;ANAPC10
Regulation of Activin Receptor Signaling Pathway (GO:0032925)	0.0489661	0.3681479	IGSF1;LEMD3;ACVR2B;ACVR2A
Regulation of Cytoplasmic Pattern Recognition Receptor Signaling Pathway (GO:0039531)	0.0489661	0.3681479	ITCH;TSPAN6;FLOT2;MAP3K7
Regulation of Exosomal Secretion (GO:1903541)	0.0489661	0.3681479	PDCD6IP;TSG101;STAM;RNF103-CHMP3
Regulation of Neurotransmitter Transport (GO:0051588)	0.0489661	0.3681479	MCTP1;CAMK2A;CASK;MCTP2
Negative Regulation of Canonical NF-kappaB Signal Transduction (GO:0043124)	0.0497777	0.3681479	TGFBR3;IRAK1;USP32;TSPAN6;DAB2IP;RORA;SLC39A8;SIRT1;TANK
Positive Regulation of Protein Metabolic Process (GO:0051247)	0.0498017	0.3681479	BARD1;GSK3B;MTPN;HDAC2;CSF1;CD80;EIF4A3;DAB2IP;STK39;LARP4;ISL1;TNF;ELAVL1;LARP1B;FOXO1;FXR1;ASPH;CSDE1;HNRNPD;IL17D;EIF3C;ATG7;OGT

CC

Term	P-value	Adjusted P-	Genes
Nucleus (GO:0005634)	1,95E+09	5,25E+10	[illegible]OX13;ZNF10;RFX3;MED9;PRKCA;SOX11;ARMC8;DICER1;ISL1;SRFBP1;HOXB2;ASF1A;HOXB6;TSPYL5;HOXB5;SLBP;EXD2;SET;ZNF391;MAPKAP1;HSPA4L;CDCA7;PRDM15;GATA3;SAMSN1;C9ORF72;RAD21;ATRIP;PPARGC1A;HOXC8;BARD1;CDK17;JUN;CDK19;RAB3IP;NFATC2;NR1D2;MEX3D;PHC3;HRNR;FAM111A;HNRNPM;AGO1;HNRNPF;TRIP6;HNRNPD;SNAI2;CDK13;ZNF136;ZNF135;CPEB4;FANK1;SETD5;ZNF493;PHF23;BHLHE41;FMN1;TCF20;TFCP2L1;PHF6;MECP2;TRIM8;MED11;MED14;RBPMS2;JARID2;ZNF367;APPL1;SS18;CNOT6L;RMI1;ZNF480;TET2;TET1;PAX6;DUSP9;GNL1;SIRT1;CDYL2;PAX2;RNF168;KAT2B;GCFC2;ZEB1;PSMA1;PSMA2;PLSCR4;MED20;MTF2;RARB;ZNF236;PLCB1;ERGIC2;SYNPO2;MIPOL1;NSD1;UBN2;ZNF229;FLNA;MEF2D;ZNF224;PMS1;RUNX1T1;TRMT10A;SF3A1;CRBN;CSNK1A1;USP9X;USP9Y;HIPK3;ZNF33B;ZNF33A;FOXR2;FABP3;NFIA;WT1;NFIB;TRMT6;CENPQ;ZNF333;ZNF451;PUS10;CCNT2;UBE2D3;ZBTB20;RORA;AHR;SALL1;SALL4;ZNF208;MAEL;OIP5;KPNA4;KMT5A;MAP3K7;KPNA3;NUDT12;KPNA1;BIVM;FBXW7;DYRK1A;CHD1L;TC2N;GTF2F1;PIAS2;FOXP2;FOXP1;HIC2;MSH4;KAT6A;PKP4;ZNF436;PHIP;RAD54L2;DGKI;NOTCH2;ANKRD17;INO80D;KMT2A;GPR88;STK39;GPATCH2L;FOXO3;FOXO1;HSP90B1;HMBOX1;ATXN1;NEUROD4;ATXN7;BAG3;NACC2;UBR5;ZKSCAN5;ZBTB18;ZNF540;AKIRIN1;ESRRG;TRMT9B;MOB1B;LSM8;PTPRA;SP3;NF1;ZNF777;MDM4;HPSE;OGT;ZNF652;MYD88;ZNF772;NFE2L2;NXT1;CELF2;PTPN22;NXT2;HHEX;DUSP10;SUMO1;ZNF407;ZNF649;PPP6R3;ZNF644;ZNF521;RPL13A;DUSP28;PTBP2;MMS22L;SLC25A15;TRAF6;ZNF516;SNURF;MCM5;LCORL;L3MBTL4;HDAC4;MTPN;SMARCD1;NFAT5;HDAC2;RNF34;ZBTB42;TTK;HDAC8;CXXC4;PPM1E;ZBTB40;PPM1G;C3ORF38;ZNF624;ASCC3;POLR2K;FKTN;FEM1B;PTPN11;PTPN12;PTPN14;PNRC1;CTCFL;KLF7;SNRK;POLR3A;NOVA1;PDCD2;PTPN2;CUL4B;PANK3;JPT2;EIF4A3;EPC1;SOX6;SOX5;SFMBT1;IFRD2;ZFP3;UHMK1;RUNX1;INPP4A;SFRP4;THAP5;XRN2;VDAC2;ZNF711;BCORL1;TP53;CBLL1;BLZF1;L3MBTL3;ANP32A;TSHZ3;DCUN1D3;TSHZ1;MYCBP2;DCUN1D4;ZBTB1;C2CD4A;STC1;PIK3R1;NLK;SEC14L2;FXR1;SPAST;ZNF706;MIER1;ZNF703;INPP5K;EGR1;XBP1;ZRANB1;DDIAS;BCL11A;SS18L1;INTS2;SIAH1;LACC1;NDUFAF6;FUBP3;NDUFAF3;GRB2;CC2D1B;DAZAP2;AHCTF1;UBXN1;PPP1R11;PPP1R10;MIA3;LARP1B;SYNCRIP;RSRC1;CHAMP1;AASS;TSC22D4;PARP1;STRBP;NCOA4;ZHX1;RHOB;HNRNPH1;TFEC;NCOA7;RGS7BP;UMPS;VGLL1;YAP1;MIS12;AKAP4;PURA;GINS1;MYEF2;SORBS1;KBTBD6;DPY19L2;STK17B;CAMK4;TRIP12;ZNF354C;NEUROG2;MPHOSPH6;ZNF354B;FOXA1;DBR1;PRDM6;WWC1;ARID4B;PRDM1;TTF2;NR3C1;RGS4;AMMECR1L;MPZ;SACS;EP300;ARID2;KDM2B;APLP2;LMO2;RSBN1;TBL1XR1;HOMEZ;DDX5;UBIAD1;CACNA1A;PDS5B;PDS5A;PRPF8;VCPIP1;IRAK1;RPRD1A;ECT2;MORC1;MYH10;SMAD2;SMAD1;CBX5;POU2F1;PHF12;NFXL1;SMAD5;AZIN1;UBE2W;NR4A1;LIN28B;DLG1;FGF14;GDNF;UBA3;BRWD1;BRWD3;KDM5A;KDM5B;GSK3B;USP37;DYRK2;THRB;RBM26;USP38;YWHAB;NVL;USP32;HSPB8;PSI
Axon (GO:0030424)	2,86E+10	5,25E+10	GSK3B;CNTF;SLC1A1;DOCK7;ELAVL4;RORA;LRP2;SLC6A1;SLC8A1;IGF1R;GRM3;ALCAM;SPAST;PCLO;CALB1;GRM7;SACS;NPTN;DIP2B;ATP7A;SNCA;EPHA5;NTRK2;SEMA6A;KCNB1;DAB2IP;DYRK1A;OPRM1;PRSS12;CD2AP;UHMK1;OLFM1;NFIB;PALLD;FEZ2;CNTN1;NF1;METAP1

			[illegible]
			MRTFB;SOX13;ZNF10;RFX3;MED9;PRKCA;SOX11;ARMC8;DICER1;ISL1;SRFBP1;HOXB2;ASF1A;HOXB6;TSPYL5;HO
			XB5;SLBP;EXD2;SET;ZNF391;MAPKAP1;HSPA4L;CDCA7;PRDM15;GATA3;SAMSN1;C9ORF72;NCAPH;HSD11B1;RA
			D21;HAO1;ATRIP;PPARGC1A;HOXC8;BARD1;CDK17;JUN;CDK19;RAB3IP;NFATC2;NR1D2;BRAF;MEX3D;PHC3;HR
			NR;FAM111A;HNRNPM;AGO1;HNRNPF;TRIP6;HNRNPD;SNAI2;CDK13;ZNF136;ZNF135;CPEB4;FANK1;SETD5;DEN
			ND1B;ZNF493;PHF23;BHLHE41;FMN1;TCF20;TFCP2L1;PHF6;MECP2;TRIM8;MED11;MED14;RBPMS2;JARID2;ZNF3
			67;APPL1;SS18;CNOT6L;RMI1;ZNF480;TET2;TET1;PAX6;DUSP9;GNL1;RBSN;SIRT1;CDYL2;PAX2;RNF168;KAT2B;G
			CFC2;ZEB1;PSMA1;PSMA2;PLSCR4;MED20;MTF2;RARB;ZNF236;PLCB1;ERGIC2;SYNPO2;KCNA6;PTGS2;MIPOL1;T
			MEM65;NSD1;UBN2;ZNF229;FLNA;MEF2D;ZNF224;PMS1;RUNX1T1;TRMT10A;SF3A1;CRBN;CSNK1A1;USP9X;USP
			9Y;HIPK3;ZNF33B;ZNF33A;FOXR2;FABP3;NFIA;WT1;NFIB;SNX14;TRMT6;CENPQ;ZNF333;ZNF451;PUS10;CCNT2;U
			BE2D3;ZBTB20;RORA;AHR;SALL1;SALL4;LAMP2;ZNF208;MAEL;OIP5;KPNA4;KMT5A;MAP3K7;KPNA3;NUDT12;KPN
			A1;BIVM;FBXW7;DYRK1A;CHD1L;TC2N;GTF2F1;PIAS2;FOXP2;FOXP1;HIC2;RAB30;RAB31;MSH4;KAT6A;PKP4;ZNF4
			36;PHIP;RAD54L2;DGKI;NOTCH2;ANKRD17;INO80D;KMT2A;GPR88;STK39;GPATCH2L;ABHD5;FOXO3;FOXO1;HSP
			90B1;HMBOX1;ATXN1;NEUROD4;ATXN7;BAG3;NACC2;UBR5;ZKSCAN5;ZBTB18;ZNF540;AKIRIN1;TOR1A;ESRRG;T
			RMT9B;MOB1B;LSM8;PTPRA;SP3;NF1;ZNF777;MDM4;HPSE;SLC49A4;OGT;ZNF652;MYD88;ZNF772;NFE2L2;NXT1;
			CELF2;PTPN22;NXT2;HHEX;DUSP10;SUMO1;AP1G1;ZNF407;ZNF649;PPP6R3;ZNF644;ZNF521;RPL13A;DUSP28;P
			TBP2;MMS22L;SLC25A15;TRAF6;ZNF516;SNURF;MCM5;LCORL;L3MBTL4;HDAC4;MTPN;SMARCD1;NFAT5;HDAC2;
			RNF34;ZBTB42;TTK;PRCP;HDAC8;CXXC4;PPM1E;ZBTB40;PPM1G;C3ORF38;ZNF624;ASCC3;E2F5;POLR2K;FKTN;C
			YB5A;FEM1B;PTPN11;PTPN12;PTPN14;PNRC1;CTCFL;KLF7;SNRK;EVI2A;POLR3A;NOVA1;PDCD2;PTPN2;CUL4B;P
			ANK3;JPT2;EIF4A3;EPC1;CCN2;SOX6;SOX5;SFMBT1;IFRD2;ZFP3;UHMK1;RUNX1;INPP4A;SFRP4;THAP5;XRN2;RUB
			CNL;VDAC2;ZNF711;UPRT;BCORL1;TP53;CBLL1;BLZF1;L3MBTL3;ANP32A;TSHZ3;DCUN1D3;TSHZ1;MYCBP2;DCU
			N1D4;ZBTB1;C2CD4A;STC1;PIK3R1;NLK;SEC14L2;FXR1;SPAST;ZNF706;MIER1;ZNF703;ATP6V0A2;INPP5K;RAB11F
			IP2;EGR1;XBP1;ZRANB1;DDIAS;BCL11A;SS18L1;SIAH2;INTS2;SIAH1;STAM;LACC1;NDUFAF6;FUBP3;NDUFAF3;GR
			B2;CC2D1B;PCSK2;DAZAP2;AHCTF1;UBXN1;PPP1R11;PPP1R10;SLC7A14;GRIK1;MIA3;SPPL3;RAB22A;LARP1B;SY
			NCRIP;RSRC1;CHAMP1;AASS;TSC22D4;PARP1;STRBP;NCOA4;ZHX1;RHOB;CCKBR;HNRNPH1;TFEC;NCOA7;RGS7
			BP;UMPS;VGLL1;YAP1;ZDHHC20;STX16;MIS12;AKAP4;PURA;FAM184A;EPS15;GINS1;MYEF2;YIPF5;SORBS1;SEC2
			3IP;ITCH;KBTBD6;DPY19L2;STK17B;CAMK4;TRIP12;ZNF354C;NEUROG2;MPHOSPH6;ZNF354B;FOXA1;CD84;DBR
Intracellular Membrane-Bounded Organelle (GO:0043231)	5,82E+08	7,12E+11	1;PRDM6;WWC1;ARID4B;PRDM1;TTF2;NR3C1;IGF1R;RGS4;RGS5;AMMECR1L;MPZ;SACS;EP300;ARID2;KDM2B;AP
			PCSK2;GSK3B;CNTF;DOCK7;ELAVL4;RORA;SLC6A1;SLC8A1;IGF1R;ARHGAP44;GRM3;TMEM266;GRM5;ALCAM;PC
			LO;CALB1;GRM7;SACS;NPTN;DIP2B;ATP7A;KPNA1;EPHA5;TSC22D4;CXADR;UNC13A;SEMA6A;DYRK1A;ANK3;OPR
			M1;PRSS12;CD2AP;UHMK1;ADCYAP1;OLFM1;PALLD;FEZ2;RGS7BP;GRIA2;CHRNA5;KLHL14;SLC1A1;CAMK2A;SL
			C1A3;LRP2;PTGS2;C9ORF72;RTN4;FXR1;INPP5B;SPAST;HRH4;GNA12;INPP5K;KCNN3;SNCA;NTRK2;CADM1;KCN
Neuron Projection (GO:0043005)	2,71E+11	0.0205049	B1;DAB2IP;DLG1;MLPH;NF1;CNTN1;STRN;KCNK2;METAP1;CPEB4
Endoplasmic Reticulum Tubular Network (GO:0071782)	2,79E+11	0.0205049	OSBPL8;SPAST;ATL3;ASPH;REEP5;STIM1;REEP3;PARP16;RTN4
Nuclear Inner Membrane (GO:0005637)	0.0014437	0.0719166	SMAD1;TMEM120B;TERB2;TMX4;DPY19L2;PTGS2;LEMD3;SIRT1
Postsynaptic Specialization Membrane (GO:0099634)	0.0014496	0.0719166	NLGN4Y;GABRB2;GRIA2;GABRA1;GRM5;GRIN2A;DLG1;PLPPR4;LRRTM2;GRIK1;CACNG2;GRIA3
			GRIA2;GSK3B;SLC1A1;ELAVL4;LRP2;C9ORF72;SLC8A1;ARHGAP44;TMEM266;GRM5;CALB1;GRM7;HRH4;SACS;DI
			P2B;ATP7A;KPNA1;EPHA5;TSC22D4;GABRA1;NTRK2;GABRA5;KCNB1;GABRA4;DYRK1A;ANK3;OPRM1;PRSS12;CD
Dendrite (GO:0030425)	0.0015676	0.0719166	2AP;GABRG1;UHMK1;MLPH;NF1;STRN;KCNK2;METAP1;CPEB4

Nuclear Membrane (GO:0031965)	0.0024021	0.0979525	ANKRD17;POM121;AHCTF1;MINDY3;HPN;ZBTB1;ITPR3;PTGS2;C9ORF72;GLE1;SPAST;SUMO1;CLMN;EPC1;SH3BGRL2;KPNA4;SEPHS1;ZNF224;RAP1GAP2;SMAD1;TMEM120B;AKIRIN1;TOR1A;LEMD3;SIRT1;INPP4A;TERB2;NR4A1;TMX4;DPY19L2;CNEP1R1;ZNF354C
Intercalated Disc (GO:0014704)	0.0028598	0.1049566	DLG1;CXADR;TMEM65;CTNNA3;PGM5;ANK3;ATP1B1;SLC8A1
Junctional Sarcoplasmic Reticulum Membrane (GO:0014701)	0.0035708	0.1191378	RYR2;ASPH;JPH1;TRDN
Calcium Channel Complex (GO:0034704)	0.0060117	0.1801959	CACNB1;RYR2;TRPC5;CACNB4;ASPH;PTPRA;CACNA2D1;CACNA1A;PKD1L1;PRKCA
SWI/SNF Complex (GO:0016514)	0.0063829	0.1801959	RB1;SMARCD1;SS18;BCL11A;ARID2;ARID1A;ARID1B
Activin Receptor Complex (GO:0048179)	0.0082631	0.2166118	ACVR2B;ACVR2A;TGFBR1
Cell-Cell Contact Zone (GO:0044291)	0.0119978	0.2935464	DLG1;CXADR;TMEM65;FLOT2;PGM5;PKP4;ANK3;ATP1B1;SLC8A1
Cortical Endoplasmic Reticulum (GO:0032541)	0.0136138	0.2938999	OSBPL8;ASPH;STIM1
cyclin/CDK Positive Transcription Elongation Factor Complex (GO:00080	0.0136138	0.2938999	RB1;CCNT2;CDK13
Cytoplasmic Stress Granule (GO:0010494)	0.0145401	0.2964583	DAZAP2;RC3H1;LARP4;PUM1;ELAVL1;C9ORF72;LARP1B;FXR1;RNF135;CAPRIN1;G3BP1;RBPMS2;G3BP2
ESC/E(Z) Complex (GO:0035098)	0.0156847	0.3029627	HDAC2;MTF2;TRIM37;SIRT1
Perineuronal Net (GO:0072534)	0.0205126	0.3512252	BCAN;NCAN;HAPLN1
Cul2-RING Ubiquitin Ligase Complex (GO:0031462)	0.0207431	0.3512252	ZYG11A;PRAMEF13;PRAMEF2;PRAMEF1;FEM1B;PRAMEF14;FEM1C;ELOC
Cul4-RING E3 Ubiquitin Ligase Complex (GO:0080008)	0.0210543	0.3512252	DCAF17;CRBN;DCAF10;DCAF12;DCAF7;DCAF13;CUL4B
cullin-RING Ubiquitin Ligase Complex (GO:0031461)	0.0248887	0.3683543	KLHL11;DCAF7;ANAPC10;FBXL20;DCAF17;PRAMEF13;PRAMEF2;PRAMEF1;PRAMEF14;FBXO4;FBXO6;FEM1C;ELOC;DCAF10;DCAF12;DCAF13;FBXO9;USP47;ZYG11A;FBXW7;FEM1B;KBTBD3;KBTBD6;KLHL7;DEPDC5;CUL4B
Protein Kinase Complex (GO:1902911)	0.0261713	0.3683543	CAB39;MAPKAP1;RICTOR;TANK;IGF1R
Glial Cell Projection (GO:0097386)	0.0263769	0.3683543	GRM3;CNTF;SLC1A1;KCNK2
Golgi Medial Cisterna (GO:0005797)	0.0289834	0.3683543	TMEM59;GOSR1;YIPF6
Neuron to Neuron Synapse (GO:0098984)	0.0289834	0.3683543	GRIA2;GRM7;SLC1A1
Perisynaptic Extracellular Matrix (GO:0098966)	0.0289834	0.3683543	BCAN;NCAN;HAPLN1
Postsynaptic Density Membrane (GO:0098839)	0.0298787	0.3683543	GRIA2;GRIN2A;GRM5;DLG1;PLPPR4;GRIK1;CACNG2;GRIA3
Glutamatergic Synapse (GO:0098978)	0.0301107	0.3683543	NLGN4Y;GSK3B;USP6;NRG1;PRSS12;PTPRD;PCLO;LRRTM3;TRAF6;SLITRK4;DLGAP1;SRGAP2B;DGKI;APPL1
Postsynaptic Density (GO:0014069)	0.0352641	0.4174823	GRIA2;NTRK2;USP6;PLPPR4;CAMK2A;GRIK1;SLC8A1;RTN4;ARHGAP44;INPP4A;ARHGAP32;GRIN2A;DLG1;GRM5;STRN;CACNG2;PDLIM5;GRIA3;LZTS3;CPEB4
CD40 Receptor Complex (GO:0035631)	0.0390132	0.4474336	CHUK;TRAF6;TANK
Basolateral Plasma Membrane (GO:0016323)	0.0453620	0.4751343	SLC38A1;CXADR;CADM1;CASK;KCNJ16;ANK3;ATP2B1;CALHM3;SLC4A4;TGFBR3;CALHM1;DLG1;SLC7A8;ERBB4;CEACAM5;NUMB;SLC16A7;SLC26A7;FLOT2;SLC39A8;SLC12A6;SLC29A1;ATP7A
Cell-Substrate Junction (GO:0030055)	0.0454714	0.4751343	RPL31;STX16;YWHAB;SYNPO2;DOCK7;TWF1;GLYAT;LPP;HSP90B1;ICAM1;ALCAM;G3BP1;GNA12;ATP6V0A2;FLNA;ITGB8;XIRP2;FLOT2;MAPK1;PGM5;ITGB6;HMCN1;TNS2;FZD1;PDCD6IP;YES1;SPRY4;RRAS2;RPL13A;CASK;SORBS1;RHOB;PROCR;PTPRA;PALLD;TRIP6;GFRAL;NUMB;ADAM9;TLN2;LAMTOR3
Ionotropic Glutamate Receptor Complex (GO:0008328)	0.0484402	0.4751343	GRIA2;GRIN2A;VWC2;GRIK1;CACNG2;GRIA3
GABA-A Receptor Complex (GO:1902711)	0.0489661	0.4751343	GABRB2;GABRA5;GABRA4;GABRG1

KEGG

Term	P-value	Adjusted P-	Genes
MAPK SIGNALING PATHWAY	2,62E+09	8,74E+10	CSF1;TNF;IGF1R;ELK4;RPS6KA6;FGF7;DUSP10;CASP3;MAP3K8;MAP3K7;MAP3K4;CHUK;PRKCB;CACNA2D1;RRAS2;PRKCA;DUSP9;TGFBR1;EREG;CACNB1;CACNB4;TRAF6;RASA2;SOS1;TP53;CACNA1A;NLK;TANK;IRAK1;ERBB4;GNA12;MAP3K20;FLNA;MAPK1;CACNG2;MAP3K2;MAP3K3;NTRK2;JUN;BRAF;MAPK10;NR4A1;KITLG;TAOK3;GDNF;NF1;GRB2;MAP3K13;LAMTOR3;MYD88
LONG-TERM DEPRESSION	4,02E+11	0.0047244	GUCY1A2;GRIA2;GUCY1A1;PRKCB;CACNA1A;PRKCA;ITPR3;BRAF;IGF1R;PPP2CB;GNAQ;GNA12;MAPK1;PLCB1;GRIA3
NEUROTROPHIN SIGNALING PATHWAY	5,24E+10	0.0047244	GSK3B;MAP3K3;NTRK2;JUN;KIDINS220;SORT1;CAMK2A;FRS2;BRAF;PTPN11;CALML4;PIK3R1;FOXO3;MAPK10;RPS6KA6;IRAK1;TRAF6;CAMK4;MAPK1;GRB2;SOS1;TP53;SH2B3
GLUTAMATERGIC SYNAPSE	5,86E+10	0.0047244	GRIA2;SLC38A1;PRKCB;SLC1A1;SLC1A3;CACNA1A;PRKCA;ITPR3;GRIK1;ADCY7;GRM3;GRIN2A;GRM5;GRM7;GNAQ;GRM8;MAPK1;DLGAP1;PLCB1;SLC38A2;GRIA3;LZTS3
ERBB SIGNALING PATHWAY	7,07E+10	0.0047244	GSK3B;JUN;PRKCB;CAMK2A;NRG1;PRKCA;BRAF;PIK3R1;EREG;BTC;MAPK10;ERBB4;ABL2;MAPK1;GRB2;SOS1;NCK1;HBEGF
COLORECTAL CANCER	1,15E+12	0.0063498	SMAD2;GSK3B;JUN;DCC;BRAF;PIK3R1;TGFBR1;EREG;CASP9;MAPK10;BCL2L11;CASP3;MAPK1;GRB2;POLK;SOS1;TP53;APPL1
LONG-TERM POTENTIATION	1,33E+12	0.0063498	GRIA2;PRKCB;CAMK2A;PRKCA;ITPR3;BRAF;CALML4;RPS6KA6;GRM5;GRIN2A;CAMK4;GNAQ;EP300;MAPK1;PLCB1
GNRH SIGNALING PATHWAY	2,11E+12	0.0088091	MAP3K2;EGR1;MAP3K3;JUN;PRKCB;CAMK2A;PRKCA;ITPR3;CALML4;ADCY7;MAPK10;GNAQ;MAPK1;GRB2;SOS1;PLCB1;MAP3K4;HBEGF
IGSF CAM SIGNALING	2,79E+11	0.0103648	GSK3B;CD84;CD80;PIK3R1;KCNA6;FOXO1;ICAM1;ROBO1;INPP5D;MPZ;SLITRK4;MAPK1;SLIT2;SRGAP1;MYH10;PPFIA2;NCK1;NLGN4Y;CD96;KIRREL1;CXADR;KIRREL2;CADM1;PRKCB;CADM2;DCC;FGG;CASK;PRKCA;PTPN11;UNC5D;ANK3;SORBS1;MTSS1;CD2AP;PTPRD;MAPK10;TRAF6;CNTN1;GRB2;SSX2IP
THYROID HORMONE SYNTHESIS	3,74E+11	0.0107144	PRKCB;GPX6;GPX8;PRKCA;ATP1A2;ITPR3;TTF2;LRP2;ATP1B1;ADCY7;HSP90B1;CREB1;GDNF;GNAQ;PLCB1
HEPATITIS B	3,83E+12	0.0107144	RB1;YWHAB;PIK3R1;TNF;CASP9;IFIH1;IRAK1;CASP3;EP300;MAPK1;MAP3K7;JUN;CHUK;PRKCB;NFATC2;PRKCA;BRAF;TGFBR1;MAPK10;CREB1;GDNF;TRAF6;GRB2;SOS1;TP53;MYD88
WNT SIGNALING PATHWAY	3,92E+12	0.0107144	GSK3B;CAMK2A;RORA;NLK;CXXC4;CCN4;EP300;RSPO3;MAP3K7;WNT3;FZD1;JUN;PRKCB;CSNK1A1;SIAH1;FZD6;NFATC2;PRKCA;DKK1;SIRT1;MAPK10;SFRP4;DAAM1;TBL1XR1;PLCB1;TP53;CSNK1A1L
LONGEVITY REGULATING PATHWAY	4,17E+11	0.0107144	KL;EHMT1;PRKCA;GLYAT;PIK3R1;FOXO3;SOD2;SIRT1;ADCY7;FOXO1;IGF1R;CREB1;GDNF;CAMK4;TP53;PPARGC1A;APPL1
PARATHYROID HORMONE SYNTHESIS, SECRETION AND ACTION	4,70E+12	0.0112119	MEF2A;KL;EGR1;PRKCB;STK39;PRKCA;ITPR3;BRAF;GATA3;ADCY7;CYP27B1;CREB1;GDNF;MMP16;GNAQ;GNA12;MAPK1;PLCB1;MEF2D;HBEGF
INSULIN SECRETION	5,38E+11	0.0119684	RYR2;PRKCB;CAMK2A;PRKCA;ATP1A2;ITPR3;ATP1B1;ADCY7;ADCYAP1;PCLO;CREB1;GDNF;GNAQ;KCNMA1;KCNN3;PLCB1
POLYCOMB REPRESSIVE COMPLEX	6,20E+11	0.0128161	HDAC2;L3MBTL3;KDM2B;SFMBT1;UBE2D3;PHC3;DCAF7;AEBP2;ASXL3;MTF2;WDR5B;EP300;JARID2;BCORL1;L3MBTL4;OGT
AMPHETAMINE ADDICTION	6,52E+11	0.0128161	GRIA2;HDAC2;JUN;PRKCB;MAOA;CAMK2A;PRKCA;CALML4;SIRT1;GRIN2A;CREB1;GDNF;CAMK4;GRIA3
ALDOSTERONE SYNTHESIS AND SECRETION	8,15E+11	0.0150543	PRKCB;PRKCE;CAMK2A;PRKCA;ATP1A2;ITPR3;CALML4;ATP2B1;ATP1B1;ADCY7;NR4A1;CREB1;GDNF;PRKD3;CAMK4;GNAQ;PLCB1
SPINOCEREBELLAR ATAXIA	8,56E+11	0.0150543	GRIA2;PSMD12;XBP1;PRKCB;CACNA1A;RORA;PRKCA;ITPR3;PIK3R1;RBPJ;PUM1;TANK;MAPK10;GRIN2A;ATXN1;FGF14;PSMA1;PSMA2;GNAQ;PPIF;VDAC2;PLCB1;GRIA3
TNF SIGNALING PATHWAY	9,33E+11	0.0155877	JUN;JAG1;CSF1;CHUK;DAB2IP;PIK3R1;PTGS2;TNF;TANK;ICAM1;MAPK10;CASP7;ITCH;CREB1;GDNF;CASP3;CCL5;MAPK1;MAP3K8;MAP3K7
PROSTATE CANCER	0.0011452	0.0179512	RB1;GSK3B;SRD5A2;CHUK;BRAF;PIK3R1;FOXO1;IGF1R;HSP90B1;CASP9;GRM5;ZEB1;CREB1;EP300;MAPK1;GRB2;SOS1;TP53

CIRCADIAN ENTRAINMENT	0.00118423	0.01795126	RYR2;GUCY1A2;GRIA2;GUCY1A1;PRKCB;CAMK2A;PRKCA;ITPR3;CALML4;ADCY7;ADCYAP1;GRIN2A;CREB1;GNAQ;MAPK1;PLCB1;GRIA3
APELIN SIGNALING PATHWAY	0.00123616	0.01795126	MEF2A;SMAD2;HDAC4;EGR1;RYR2;JAG1;PRKCE;RRAS2;PRKCA;ITPR3;CALML4;ADCY7;SLC8A1;TGFBR1;GNAQ;CAMK4;MAPK1;CCN2;PLIN1;PLCB1;PPARGC1A;MEF2D
GASTRIC ACID SECRETION	0.00157770	0.02195635	PRKCB;CAMK2A;KCNJ16;PRKCA;ATP1A2;ITPR3;CALML4;ATP1B1;ADCY7;CCKBR;GNAQ;SLC26A7;PLCB1;KCNK2
MICRORNAS IN CANCER	0.00180133	0.02332559	NOTCH2;HDAC4;HDAC2;PRKCB;PRKCE;PRKCA;DICER1;PIK3R1;PTGS2;SIRT1;FOXP1;BCL2L11;ZEB1;MMP16;CASP3;DDIT4;EP300;MAPK1;MDM4;GRB2;SOS1;TP53;RECK;WNT3
PROTEOGLYCANS IN CANCER	0.00181576	0.02332559	DDX5;SDC2;CAMK2A;ITPR3;PIK3R1;TNF;IGF1R;ERBB4;CASP3;FLNA;MAPK1;WNT3;FZD1;SMAD2;PRKCB;FZD6;RRAS2;FRS2;PRKCA;BRAF;PTPN11;ANK3;GRB2;HPSE;SOS1;PPP1R12B;TP53;HBEGF
GLIOMA	0.00206262	0.02551543	RB1;PRKCB;CAMK2A;PRKCA;BRAF;CALML4;PIK3R1;IGF1R;CAMK4;MAPK1;GRB2;POLK;SOS1;TP53
PATHWAYS IN CANCER	0.00258884	0.03088116	RB1;GSK3B;HHIP;MIA3;CALML4;TTF2;IGF1R;CASP9;FGF7;CASP7;CASP3;EP300;ELOC;POLK;APPL1;CHUK;PRKCB;DCC;NCOA4;PRKCA;TGFBR1;RUNX1;TRAF6;RARB;PLCB1;IL6ST;SOS1;TP53;NOTCH2;HDAC2;CAMK2A;PIK3R1;PTGS2;ADCY7;TANK;CSF2RA;FOXO1;HSP90B1;DLL4;BCL2L11;GNA12;MAPK1;CTNNA3;WNT3;RUNX1T1;FZD1;SMAD2;JUN;JAG1;FZD6;BRAF;LAMB1;EPOR;MAPK10;KITLG;IL7;GNAQ;CCDC6;GRB2;NFE2L2
HEPATOCELLULAR CARCINOMA	0.00295824	0.03305570	RB1;FZD1;SMAD2;GSK3B;SMARCD1;PRKCB;CSNK1A1;FZD6;PRKCA;BRAF;PIK3R1;ARID1A;ARID1B;TGFBR1;IGF1R;MAPK1;GRB2;POLK;ARID2;SOS1;TP53;WNT3;CSNK1A1L;NFE2L2
ADRENERGIC SIGNALING IN CARDIOMYOCYTES	0.00305983	0.03305570	RYR2;CACNA2D1;CAMK2A;PRKCA;ATP1A2;CALML4;ATP2B1;ATP1B1;ADCY7;SLC8A1;CACNB1;PPP2CB;CACNB4;CREB1;GDNF;PPP2R2B;GNAQ;MAPK1;AGTR2;PLCB1;CACNG2;KCNK2
NOTCH SIGNALING PATHWAY	0.00306804	0.03305570	LFNG;NOTCH2;DLL4;KAT2B;HDAC2;ITCH;ATXN1;JAG1;NUMB;EP300;RBPJ;DTX4
NICOTINE ADDICTION	0.00345722	0.03539599	GABRB2;GRIA2;GABRA1;GRIN2A;GABRA5;GABRA4;CACNA1A;GRIA3;GABRG1
NON-SMALL CELL LUNG CANCER	0.00349720	0.03539599	RB1;PRKCB;PRKCA;BRAF;PIK3R1;FOXO3;CASP9;RARB;MAPK1;GRB2;POLK;SOS1;TP53
SPHINGOLIPID SIGNALING PATHWAY	0.00369227	0.03627122	CERS6;PRKCB;PRKCE;PRKCA;PIK3R1;TNF;TANK;SGPP1;MAPK10;PPP2CB;SPTLC2;PPP2R2B;GNAQ;GNA12;MAPK1;S1PR3;PLCB1;TP53;CERS2
SALIVARY SECRETION	0.00408148	0.03826169	GUCY1A2;GUCY1A1;PRKCB;PRKCA;ATP1A2;ITPR3;CALML4;ATP2B1;ATP1B1;ADCY7;RBL1;GNAQ;KCNMA1;CHAMP1;PLCB1
FERROPTOSIS	0.00412401	0.03826169	SLC38A1;NCOA4;VDAC2;SLC39A8;ACSL4;ACSL3;GCLM;ATG7;TP53
ENDOCRINE AND OTHER FACTOR-REGULATED CALCIUM REABSORPTION	0.00519347	0.04412272	KL;CALB1;PRKCB;GNAQ;PRKCA;ATP1A2;ATP2B1;ATP1B1;PLCB1;SLC8A1
AGE-RAGE SIGNALING PATHWAY IN DIABETIC COMPLICATIONS	0.00520636	0.04412272	SMAD2;EGR1;JUN;PRKCB;PRKCE;PRKCA;PIK3R1;TNF;F3;FOXO1;TGFBR1;ICAM1;MAPK10;CASP3;MAPK1;PLCB1
ENDOMETRIAL CANCER	0.00528255	0.04412272	CASP9;GSK3B;MAPK1;CTNNA3;BRAF;GRB2;POLK;PIK3R1;SOS1;FOXO3;TP53
TGF-BETA SIGNALING PATHWAY	0.00528415	0.04412272	SMAD2;SMAD1;HDAC2;IGSF1;INHBB;LEMD3;TNF;SMAD5;GDF5;ACVR2B;TGFBR1;ACVR2A;PPP2CB;RBL1;EP300;MAPK1;E2F5
LIPID AND ATHEROSCLEROSIS	0.00594483	0.04842866	GSK3B;CAMK2A;CALML4;PIK3R1;TANK;TNF;ICAM1;HSP90B1;CASP9;CASP7;IRAK1;CASP3;CCL5;MAPK1;MAP3K7;XBP1;JUN;POU2F1;CHUK;NFATC2;PRKCA;SOD2;MAPK10;TRAF6;PLCB1;TP53;MYD88;NFE2L2
CHRONIC MYELOID LEUKEMIA	0.00636717	0.05063423	RB1;HDAC2;CHUK;BRAF;PTPN11;PIK3R1;TGFBR1;RUNX1;MAPK1;GRB2;POLK;SOS1;TP53
CHAGAS DISEASE	0.00695135	0.05399424	SMAD2;JUN;CHUK;PIK3R1;TNF;TGFBR1;MAPK10;PPP2CB;IRAK1;PPP2R2B;GNAQ;TRAF6;CCL5;MAPK1;PLCB1;MYD88
DOPAMINERGIC SYNAPSE	0.00737010	0.05594579	GRIA2;GSK3B;PRKCB;MAOA;CAMK2A;CACNA1A;PRKCA;ITPR3;CALML4;MAPK10;PPP2CB;GRIN2A;CREB1;GDNF;PPP2R2B;GNAQ;PLCB1;GRIA3;SCN1A
SIGNALING PATHWAYS REGULATING PLURIPOTENCY OF STEM CELLS	0.00777284	0.05769175	FZD1;SMAD2;SMAD1;GSK3B;FZD6;INHBB;PAX6;PIK3R1;ISL1;SMAD5;DUSP9;ACVR2B;ACVR2A;IGF1R;KAT6A;MAPK1;GRB2;IL6ST;JARID2;WNT3
CUSHING SYNDROME	0.00811243	0.05840707	RB1;FZD1;GSK3B;KMT2A;FZD6;CAMK2A;PRKCA;ITPR3;BRAF;MIA3;AHR;ADCY7;NR4A1;CREB1;GDNF;GNAQ;WDR5B;MAPK1;PLCB1;KCNK2;WNT3
MELANOGENESIS	0.00833350	0.05840707	FZD1;GSK3B;PRKCB;CAMK2A;FZD6;PRKCA;CALML4;ADCY7;KITLG;CREB1;GNAQ;EP300;MAPK1;PLCB1;WNT3
CGMP-PKG SIGNALING PATHWAY	0.00839383	0.05840707	MEF2A;GUCY1A2;GUCY1A1;PRKCE;NFATC2;ITPR3;ATP1A2;CALML4;ATP2B1;ATP1B1;ADCY7;SLC8A1;CREB1;GDNF;GNAQ;KCNMA1;GNA12;PPIF;VDAC2;MAPK1;PLCB1;MEF2D

OXYTOCIN SIGNALING PATHWAY	0.0087309	0.0595132	GUCY1A2;RYR2;GUCY1A1;JUN;PRKCB;CACNA2D1;CAMK2A;NFATC2;PRKCA;ITPR3;CALML4;PTGS2;ADCY7;CACNB1;CACNB4;GNAQ;CAMK4;MAPK1;PLCB1;PPP1R12B;CACNG2
MTOR SIGNALING PATHWAY	0.0100826	0.0672124	FZD1;GSK3B;CHUK;CAB39;PRKCB;MAPKAP1;FZD6;PRKCA;BRAF;PIK3R1;TNF;IGF1R;RPS6KA6;DDIT4;MAPK1;GRB2;RICTOR;SOS1;DEPDC5;LAMTOR3;WNT3
HUMAN CYTOMEGALOVIRUS INFECTION	0.0102629	0.0672124	RB1;GSK3B;ITPR3;CALML4;PIK3R1;PTGS2;TANK;ADCY7;TNF;SEC14L2;CASP9;CASP3;CCL5;CXCR2;GNA12;MAPK1;CCR1;CHUK;PRKCB;NFATC2;PRKCA;CREB1;GDNF;GNAQ;GRB2;PLCB1;SOS1;TP53
BREAST CANCER	0.0112853	0.0710312	RB1;NOTCH2;FZD1;GSK3B;JUN;JAG1;CSNK1A1;FZD6;BRAF;PIK3R1;IGF1R;DLL4;FGF7;MAPK1;GRB2;POLK;SOS1;TP53;WNT3;CSNK1A1L
VIRAL CARCINOGENESIS	0.0113973	0.0710312	RB1;HDAC4;JUN;HDAC2;YWHAB;HPN;PRKCA;GTF2H1;PIK3R1;RBPJ;HDAC8;TANK;KAT2B;DLG1;CREB1;RBL1;GDNF;CASP3;RASA2;EP300;MAPK1;GRB2;IL6ST;TP53
CHEMICAL CARCINOGENESIS - RECEPTOR ACTIVATION	0.0114840	0.0710312	RB1;NOTCH2;CACNA1A;MIA3;PIK3R1;AHR;ADCY7;HSP90B1;DLL4;RPS6KA6;FGF7;MAPK1;KPNA4;KPNA3;KPNA1;JUN;JAG1;PRKCB;PAQR5;UGT2B17;PRKCA;CREB1;GDNF;GRB2;SOS1
COCAINE ADDICTION	0.0119978	0.0715036	GPSM1;GRM3;GRIA2;GRIN2A;JUN;CREB1;GDNF;MAOA;PRKCA
CELL CYCLE	0.0124205	0.0715036	RB1;SMAD2;GSK3B;HDAC2;YWHAB;TTK;PDS5B;PDS5A;HDAC8;CDC14A;ANAPC10;SLC25A15;PPP2CB;DBF4;RBL1;RAD21;EP300;MCM5;E2F5;TP53;ZNF367
T CELL RECEPTOR SIGNALING PATHWAY	0.0125433	0.0715036	GSK3B;JUN;CHUK;NFATC2;PTPN11;PIK3R1;TNF;MAPK10;PPP2CB;DLG1;PPP2R2B;MAPK1;MAP3K8;GRB2;SOS1;MAP3K7;NCK1
THYROID HORMONE SIGNALING PATHWAY	0.0125433	0.0715036	NOTCH2;GSK3B;HDAC2;THRB;PRKCB;PRKCA;ATP1A2;PIK3R1;ATP1B1;FOXO1;CASP9;KAT2B;MED14;EP300;MAPK1;PLCB1;TP53
YERSINIA INFECTION	0.0126308	0.0715036	GSK3B;JUN;FYB1;CHUK;WIPF2;NFATC2;PIK3R1;TNF;TANK;MAPK10;RPS6KA6;IRAK1;GNAQ;TRAF6;MAPK1;PIP5K1B;MAP3K7;MYD88;SKAP2
PEROXISOME	0.0132190	0.0735860	PEX16;ABCD3;ACSL4;ACSL3;GLYAT;SOD2;PEX13;NUDT7;PEX5L;AMACR;HAO1;FAR2;NUDT12
FOXO SIGNALING PATHWAY	0.0141354	0.0772773	CHUK;BRAF;GLYAT;PIK3R1;NLK;FOXO3;SOD2;SIRT1;FOXO1;TGFBR1;IGF1R;MAPK10;BCL2L11;EP300;MAPK1;GRB2;SGK3;SOS1
IL-17 SIGNALING PATHWAY	0.0144848	0.0772773	GSK3B;JUN;CHUK;PTGS2;TNF;ELAVL1;TANK;HSP90B1;MAPK10;CASP3;TRAF6;MAPK1;IL17D;MAP3K7
TRANSCRIPTIONAL MISREGULATION IN CANCER	0.0145762	0.0772773	SMAD1;SS18;DDX5;HDAC2;CCNT2;KMT2A;SS18L1;LMO2;ETV1;PAX5;FOXO1;IGF1R;RUNX1;ELK4;HHEX;ZEB1;GDNF;SIX4;WT1;TSPAN7;POLK;TP53;GRIA3;RUNX1T1
VASCULAR SMOOTH MUSCLE CONTRACTION	0.0152130	0.0793932	GUCY1A2;GUCY1A1;CALCRL;CALCB;PRKCB;PRKCE;PRKCA;ITPR3;BRAF;CALML4;ADCY7;GNAQ;KCNMA1;GNA12;MAPK1;PLCB1;PPP1R12B;MYH10
GAP JUNCTION	0.0159599	0.0820095	MAP3K2;GUCY1A2;GUCY1A1;PRKCB;PRKCA;ITPR3;ADCY7;GRM5;GNAQ;MAPK1;GRB2;SOS1;PLCB1
UBIQUITIN MEDIATED PROTEOLYSIS	0.0167622	0.0848270	UBE3C;FBXW7;UBE2D3;SIAH1;UBE2A;PIAS2;ANAPC10;RHOBTB1;UBE2W;HERC3;ITCH;TRAF6;UBR5;FBXO4;UBA3;TRIM37;TRIP12;ELOC;CUL4B
OTHER TYPES OF O-GLYCAN BIOSYNTHESIS	0.0181967	0.0907119	LFNG;POFUT2;POFUT1;GALNT18;GALNT1;POC1B-GALNT4;OGT;GALNT10
LONGEVITY REGULATING PATHWAY - MULTIPLE SPECIES	0.0186285	0.0914988	HDAC2;PRKCA;PIK3R1;GLYAT;FOXO3;SOD2;SIRT1;ADCY7;FOXO1;IGF1R
NUCLEOCYTOPLASMIC TRANSPORT	0.0197841	0.0957666	POM121;AHCTF1;NXT1;NUP210;EIF4A3;NXT2;GLE1;NXF1;SUMO1;TMEM33;KPNA4;NUP43;KPNA3;NUP58;KPNA1
HIPPO SIGNALING PATHWAY	0.0207003	0.0987703	FZD1;SMAD2;YAP1;SMAD1;GSK3B;YWHAB;WWC1;FZD6;GDF5;TGFBR1;AMOT;MOB1B;PPP2CB;DLG1;PPP2R2B;RASSF6;SNAI2;CTNNA3;CCN2;WNT3
RETROGRADE ENDOCANNABINOID SIGNALING	0.0218775	0.1014875	GRIA2;GABRB2;GABRA1;PRKCB;GABRA5;GABRA4;CACNA1A;PRKCA;ITPR3;PTGS2;ADCY7;GABRG1;MAPK10;GRM5;GNAQ;MAPK1;NDUFS1;PLCB1;GRIA3
GASTRIC CANCER	0.0218775	0.1014875	RB1;FZD1;SMAD2;GSK3B;CSNK1A1;FZD6;BRAF;PIK3R1;TGFBR1;FGF7;RARB;MAPK1;GRB2;CTNNA3;POLK;SOS1;TP53;WNT3;CSNK1A1L
PHOSPHOLIPASE D SIGNALING PATHWAY	0.0233258	0.1067239	RRAS2;PRKCA;PTPN11;TTF2;PIK3R1;ADCY7;GRM3;GRM5;KITLG;GRM7;GRM8;GNA12;CXCR2;MAPK1;PIP5K1B;GRB2;SOS1;PLCB1;DGKI

GROWTH HORMONE SYNTHESIS, SECRETION AND ACTION	0.02379949	0.10681631	GSK3B;PRKCB;PRKCA;ITPR3;PIK3R1;ADCY7;GHR;MAPK10;CREB1;GDNF;GNAQ;EP300;MAPK1;GRB2;SOS1;PLCB1
AFRICAN TRYPANOSOMIASIS	0.02424603	0.10681631	PRKCB;GNAQ;PRKCA;PLCB1;TNF;MYD88;ICAM1
PATHOGENIC ESCHERICHIA COLI INFECTION	0.02430550	0.10681631	JUN;CHUK;WIPF2;BRK1;PTPN11;TTF2;TANK;TNF;CASP9;MAPK10;OCLN;CASP7;BAIAP2L1;IRAK1;MYO5B;CASP3;TRAF6;GNA12;MAPK1;CLDN16;MAP3K7;MYH10;MYD88;NCK1
CALCIUM SIGNALING PATHWAY	0.02468083	0.10705711	RYR2;CAMK2A;CACNA1A;ITPR3;CALML4;ADCY7;SLC8A1;GRIN2A;FGF7;GRM5;ERBB4;NTRK2;MCOLN3;PRKCB;NFATC2;PRKCA;PHKB;ATP2B1;TRDN;CCKBR;STIM1;GDNF;ASPH;CAMK4;GNAQ;PPIF;VDAC2;PLCB1;SOS1
ADHERENS JUNCTION	0.02902153	0.12427169	PTPN1;YES1;SORBS1;NLK;TGFBR1;IGF1R;PTPRB;EP300;MAPK1;SNAI2;CTNNA3;MAP3K7;SSX2IP
RENAL CELL CARCINOMA	0.03405555	0.14398172	JUN;EP300;MAPK1;MIA3;BRAF;PTPN11;GRB2;ELOC;PIK3R1;SOS1
GLUCAGON SIGNALING PATHWAY	0.03482252	0.14538405	CAMK2A;PRKCA;ITPR3;PHKB;CALML4;SIRT1;FOXO1;CREB1;GDNF;GNAQ;EP300;PGAM4;PLCB1;PPARGC1A
NEUROACTIVE LIGAND SIGNALING	0.03553290	0.14611822	P2RY12;GABRB2;GABRA1;GRIA2;PRKCB;GABRA5;GABRA4;SLC1A1;SLC1A3;PRKCA;GRIK1;OPRM1;SLC6A1;ADCY7;GABRG1;GRM3;GRIN2A;GRM5;GRM7;GNAQ;GRM8;PLCB1;GRIA3
OSTEOCLAST DIFFERENTIATION	0.03587333	0.14611822	JUN;CSF1;CHUK;NFATC2;ITPR3;PIK3R1;TMEM64;TNF;TANK;TGFBR1;MAPK10;CREB1;TRAF6;CAMK4;MAPK1;GRB2;MAP3K7
GABAERGIC SYNAPSE	0.03813198	0.15163015	GABRB2;GABRA1;SLC38A1;PRKCB;GABRA5;GABRA4;CACNA1A;PRKCA;SLC6A1;ADCY7;SLC38A2;GABRG1
ESTROGEN SIGNALING PATHWAY	0.03813452	0.15163015	JUN;PRKCA;ITPR3;CALML4;PIK3R1;OPRM1;ADCY7;HSP90B1;CREB1;GDNF;GNAQ;MAPK1;GRB2;SOS1;PLCB1;FKBP5;HBEGF
PANCREATIC CANCER	0.03958124	0.15553100	RB1;CASP9;SMAD2;MAPK10;CHUK;MAPK1;BRAF;POLK;PIK3R1;TP53;TGFBR1
HUMAN T-CELL LEUKEMIA VIRUS 1 INFECTION	0.04120957	0.16004649	RB1;PIK3R1;ADCY7;TNF;ANAPC10;ICAM1;ELK4;EP300;MAPK1;SMAD2;EGR1;MAP3K3;JUN;CHUK;NFATC2;PRKCA;TGFBR1;MAPK10;KAT2B;DLG1;CREB1;GDNF;VDAC2;TLN2;TP53
INSULIN RESISTANCE	0.04285282	0.16123592	GSK3B;PTPN1;PRKCB;PRKCE;PTPN11;PIK3R1;TNF;FOXO1;MAPK10;RPS6KA6;CREB1;PTPRA;PPARGC1A;OGT
AXON GUIDANCE	0.04295576	0.16123592	EPHA5;GSK3B;TRPC5;SEMA6A;DCC;SEMA6D;CAMK2A;SEMA3G;NFATC2;PRKCA;PTPN11;UNC5D;PIK3R1;SEMA4G;SSH2;ROBO1;MAPK1;PLXNC1;SLIT2;SRGAP1;NCK1
INSULIN SIGNALING PATHWAY	0.04296406	0.16123592	GSK3B;PTPN1;PRKCA;PHKB;BRAF;CALML4;PIK3R1;HOOK3;SORBS1;FOXO1;MAPK10;FLOT2;MAPK1;GRB2;SOS1;PPARGC1A;RHOQ
RENIN SECRETION	0.04441448	0.16314912	GUCY1A2;ADCYAP1;GUCY1A1;CREB1;GNAQ;KCNMA1;PRKCA;ITPR3;CALML4;PLCB1
HEPATITIS C	0.04445081	0.16314912	RB1;GSK3B;CHUK;YWHAB;BRAF;PIK3R1;TNF;TANK;CASP9;PPP2CB;OCLN;PPP2R2B;CASP3;TRAF6;MAPK1;GRB2;CLDN16;SOS1;TP53
PHOSPHATIDYLINOSITOL SIGNALING SYSTEM	0.04530433	0.16447443	MTMR1;PRKCB;PRKCA;ITPR3;CALML4;PIK3R1;MTM1;INPP4A;INPP5B;INPP5D;PIP5K1B;PLCB1;DGKI
MORPHINE ADDICTION	0.04776101	0.17152880	GABRB2;GABRA1;PRKCB;GABRA5;GABRA4;CACNA1A;PRKCA;OPRM1;PDE7B;PDE7A;ADCY7;GABRG1

Jensen			
Term	P-value	Adjusted P-	Genes
DISEASE	3,41E+11	0.0078815	RB1;RPL31;COL12A1;EHMT1;RBPJ;ANTXR2;SLC4A4;ANTXR1;LSM11;PDK3;CCN4;HMCN1;SLC12A6;SCN1A;CUBN;CACNA2D1;WDR72;PRKCA;SOX11;ACSL4;DICER1;GABRG1;RUNX1;SFRP4;RUFY3;NHLRC2;TP53;FREM1;ALDH7A1;GNE;SET;SLC22A5;MAOA;SDC2;TSHZ1;IGSF1;GATA3;PIK3R1;PCDH19;C9ORF72;NCAPH;C2;PKHD1;ADH4;CYP27B1;GRIN2A;SPAST;MAP2;RAD21;ARSK;ATP6V0A2;INPP5K;ATG7;SCN3A;ZBTB7C;SLC19A2;BARD1;MSR1;JUN;XBP1;CDK19;JAG1;YES1;DTNA;FZD6;BRAF;PUM1;GDF5;AIMP1;FAM111A;LACC1;CCDC88A;GJB2;HAL;NDUFAF6;AGO1;MYO5B;TNFSF4;ACER3;CALU;NDUFAF3;SNAI2;TECPR2;ODAPH;DENND1B;GABRB2;RYR2;MOCS2;FASTKD2;HEXB;SLC7A14;KIF14;MIA3;HCCS;GRIK2;SLC6A1;PHF6;JPH1;MECP2;IFIH1;LFNG;MED11;PCLO;SPTLC2;TRIM2;TCTN1;IDS;AASS;APPL1;SS18;NCOA4;TET2;RRAS2;PAX6;AP3B1;PAX5;CYP7B1;SYN2;ANO5;PAX2;RHOB;VWA8;ZEB1;F9;CEACAM5;KCNMA1;PIGK;RARB;TMEM106B;L2HGDH;PLIN1;DEPDC5;PLCB1;RGR;YAP1;ECHS1;ATL3;STX17;COL11A1;ADCY3;NSD1;FLNA;CTNNA3;EPS15;MEF2D;PMS1;RUNX1T1;PEX16;GABRA1;TRMT10A;KCNB1;KIDINS220;GABRA5;GABRA4;YIPF5;DAB2IP;PEX13;KITLG;DPY19L2;WT1;SNX14;KLHL7;CCDC6;MSRB3;FBXO28;F13A1;RORA;PRDM1;AHR;NR3C1;TNF;HOXA13;CDC14A;IGF1R;GRIP1;SMCHD1;SALL1;POFUT1;SALL4;LAMP2;SACS;EP300;MAP3K8;DENND5A;SLC39A8;ARID2;MAP3K7;KPNA3;KRT6B;CD96;KCNH5;USP6;LMO2;DYRK1A;DNAJB13;CASK;CHD1L;LEMD3;ATP1B1;FOXP2;CACNB4;STIM1;MSH4;KAT6A;SMS;ANKS6;ADAM9;ROR1;IL6ST;FKBP5;FBN2;NOTCH2;GRIA2;RNASEH2B;CALCB;KMT2A;IREB2;AK2;CACNA1A;SLC1A3;ITPR3;ABHD5;FOXO3;PRPF8;FOXO1;MTM1;ATXN1;IRAK1;ATXN7;BAG3;ECT2;WNT3;SPECC1;SMAD2;KL;TOR1A;FANCM;SRD5A2;ATP8B1;SRD5A3;CRIM1;DCDC2;PHKB;NEB;A2ML1;HPS5;PTPRD;ARHGAP32;FGF14;AMACR;GDNF;GNAQ;CAPRIN1;DMXL2;COL5A2;NF1;TUBGCP4;TGFBI;BRWD1;MYD88;KDM5A;KDM5B;GSK3B;CELF2;HSPB8;FHL1;DOCK7;MTR;PTPN22;BICD2;GHR;GMPPB;SPRED1;ZIC2;GRM7;TRPS1;ZIC1;DBT;GATAD1;TMEM107;HYDIN;ITGB6;ZNF644;ATP7A;ZNF521;RUBCN;PDCD6IP;DCC;VPS13C;FGG;VPS13A;NRG1;ETV1;TENT5A;OPRM1;ARID1A;ARID1B;TGFBR1;DNAJC3;ACTA1;SLC25A15;SETBP1;RP2;TRAF6;RASA2;RBM20;NDUFS1;SOS1;FXN;HDAC4;COLEC10;HLF;PLAG1;DNAH5;BRAT1;PKD1L1;ATP1A2;NDNF;LIN9;LRP2;PPM1K;HDAC8;SELENOI;DLL4;ACAN;DCAF17;ARL13B;CNR1;ERBB4;MXI1;RAB28;SH2B3;FKTN;SNCA;NTRK2;SLC35A3;AMFR;EYA4;SPRY4;SYT14;LAMB1;PTPN11;ATP2B1;DDHD1;ACVR2B;EPOR;PNKD;EVI2A;POLR3A;MLPH;APC;PPP2R2B;ASXL3;TRIM37;KRAS;RECK;PTPN2
RASOPATHY	5,20E+10	0.0078815	SPRED1;RASA2;NF1;PTPN11;BRAF;A2ML1;KRAS;SOS1
CANCER	6,38E+10	0.0078815	RB1;SALL4;CCN4;EP300;MAP3K8;ZNF521;SS18;USP6;DCC;NCOA4;LMO2;TET2;RRAS2;PRKCA;NRG1;ETV1;PAX5;ARID1A;TGFBR1;RUNX1;RHOB;STIM1;RUFY3;KAT6A;CEACAM5;RARB;TP53;YAP1;SET;CALCB;HLF;KMT2A;PLAG1;LRP2;LIN9;FOXO3;FOXO1;NSD1;MXI1;ECT2;EPS15;WNT3;ZBTB7C;RUNX1T1;BARD1;SPECC1;MSR1;JUN;XBP1;YES1;SRD5A2;DAB2IP;BRAF;KITLG;EVI2A;APC;WT1;CCDC6;NF1;TRIM37;KRAS;RECK;MYD88

DISEASE OF CELLULAR PROLIFERATION	7,65E+10	0.0078815	RB1;SALL4;CCN4;EP300;MAP3K8;ZNF521;SS18;USP6;DCC;NCOA4;LMO2;TET2;RRAS2;PRKCA;NRG1;ETV1;PAX5;ARID1A;TGFBR1;RUNX1;RHOB;STIM1;RUFY3;KAT6A;CEACAM5;RARB;TP53;YAP1;SET;CALCB;HLF;KMT2A;PLAG1;LRP2;LIN9;FOXO3;FOXO1;NSD1;MXI1;ECT2;EPS15;WNT3;ZBTB7C;RUNX1T1;BARD1;SPECC1;MSR1;JUN;XBP1;YES1;SRD5A2;DAB2IP;BRAF;KITLG;EVI2A;APC;WT1;CCDC6;NF1;TRIM37;KRAS;RECK;MYD88
NOONAN SYNDROME	2,58E+11	0.0212343	RASA2;A2ML1;PTPN11;BRAF;KRAS;SOS1
CENTRAL NERVOUS SYSTEM DISEASE	4,23E+12	0.0290471	FBXO28;RORA;SACS;PDK3;DENND5A;SLC12A6;KPNA3;SCN1A;KCNH5;CACNA2D1;CACNB4;NHLRC2;ALDH7A1;GRIA2;IREB2;SLC1A3;CACNA1A;ITPR3;PCDH19;C9ORF72;CYP27B1;GRIN2A;ATXN1;SPAST;ATXN7;ATG7;SCN3A;CDK19;JAG1;TOR1A;PUM1;PTPRD;AIMP1;CCDC88A;FGF14;AGO1;CAPRIN1;DMXL2;ACER3;TECPR2;GSK3B;GABRB2;HEXB;CELF2;HSPB8;DOCK7;GRIK2;SLC6A1;PHF6;BICD2;MECP2;MED11;PCLO;SPTLC2;GRM7;TRIM2;ZIC1;TCTN1;TMEM107;ATP7A;RUBCN;DCC;VPS13C;VPS13A;CYP7B1;DNAJC3;KCNMA1;PIGK;TMEM106B;DEPDC5;PLCB1;FXN;YAP1;HDAC4;ECHS1;BRAT1;ATP1A2;SELENOI;ARL13B;CNR1;ERBB4;MEF2D;SNCA;NTRK2;GABRA1;SLC35A3;KCNB1;KIDINS220;GABRA5;AMFR;YIPF5;SYT14;DDHD1;PNKD;POLR3A;PPP2R2B;SNX14
DISEASE OF MENTAL HEALTH	9,18E+11	0.0540570	KDM5A;KDM5B;GABRA5;GABRA4;DYRK1A;SOX11;OPRM1;ARID1A;TNF;ARID1B;SYN2;FOXP2;GABRG1;MECP2;ADH4;GRIN2A;SETBP1;ASXL3;SMS;ARID2;FKBP5;SNCA
AUTOSOMAL DOMINANT DISEASE	0.0010642	0.0548097	HOXA13;BICD2;SPRED1;SALL1;SALL4;TRPS1;EP300;HMCN1;ARID2;KRT6B;APPL1;SCN1A;SOX11;DICER1;LEMD3;ARID1A;ANO5;ARID1B;PAX2;FOXP2;TGFBR1;ACTA1;F9;STIM1;SETBP1;DEPDC5;TP53;NOTCH2;FBN2;TSHZ1;SDC2;CACNA1A;PIK3R1;C2;ATXN1;ATXN7;MAP2;RAD21;FLNA;PMS1;KCNB1;FZD6;BRAF;PTPN11;PUM1;GJB2;PNKD;FGF14;WT1;PPP2R2B;SNX14;NF1;SNAI2;TGFBI
EPILEPSY	0.0014367	0.0640650	HDAC4;GABRB2;GRIA2;CELF2;BRAT1;FBXO28;DOCK7;CACNA1A;RORA;ATP1A2;GRIK2;PCDH19;SLC6A1;PHF6;MED11;GRIN2A;GRM7;DENND5A;SCN3A;SCN1A;GABRA1;NTRK2;CDK19;KCNH5;SLC35A3;KCNB1;GABRA5;CACNA2D1;YIPF5;VPS13A;PUM1;CCDC88A;CACNB4;AGO1;KCNMA1;DMXL2;PIGK;PLCB1;DEPDC5;ALDH7A1
BRAIN DISEASE	0.0015549	0.0640650	GABRB2;CELF2;FBXO28;DOCK7;RORA;GRIK2;SLC6A1;PHF6;MECP2;MED11;GRM7;TCTN1;TMEM107;ZIC1;SACS;DENND5A;SCN1A;KCNH5;DCC;CACNA2D1;VPS13A;CACNB4;KCNMA1;PIGK;TMEM106B;PLCB1;DEPDC5;FXN;ALDH7A1;HDAC4;YAP1;GRIA2;BRAT1;CACNA1A;ATP1A2;PCDH19;GRIN2A;ATXN1;ARL13B;ATXN7;MEF2D;SCN3A;GABRA1;NTRK2;CDK19;TOR1A;SLC35A3;KCNB1;GABRA5;YIPF5;PUM1;AIMP1;CCDC88A;PNKD;POLR3A;FGF14;PPP2R2B;AGO1;SNX14;DMXL2;ACER3
DEVELOPMENTAL DISORDER OF MENTAL HEALTH	0.0023548	0.0783116	KDM5A;KDM5B;DYRK1A;SOX11;ARID1A;ARID1B;SYN2;FOXP2;MECP2;SETBP1;ASXL3;SMS;ARID2

NERVOUS SYSTEM DISEASE	0.0023802	0.0783116	RB1;FBXO28;RORA;AHR;SLC4A4;TNF;CDC14A;GRIP1;SMCHD1;SALL1;SALL4;SACS;PDK3;DENND5A;HMCN1;SLC12A6;KPNA3;SCN1A;KCNH5;CACNA2D1;ATP1B1;CACNB4;ADAM9;ROR1;NHLRC2;FREM1;ALDH7A1;GRIA2;IREB2;CACNA1A;SLC1A3;ITPR3;ABHD5;PCDH19;PRPF8;C9ORF72;NCAPH;FOXO1;C2;CYP27B1;GRIN2A;ATXN1;SPAST;ATXN7;BAG3;MAP2;INPP5K;ATG7;SCN3A;BARD1;CDK19;JAG1;TOR1A;SRD5A3;CRIM1;DCDC2;PUM1;AIMP1;PTPRD;FAM111A;CCDC88A;GJB2;FGF14;AGO1;CAPRIN1;DMXL2;ACER3;TUBGCP4;TGFBI;TECPR2;GABRB2;GSK3B;HEXB;CELF2;HSPB8;DOCK7;SLC7A14;KIF14;HCCS;GRIK2;SLC6A1;PHF6;JPH1;BICD2;MECP2;MED11;PCLO;ZIC2;GRM7;SPTLC2;TRIM2;ZIC1;TCTN1;TMEM107;ZNF644;ATP7A;RUBCN;PDCD6IP;DCC;VPS13C;VPS13A;PAX6;CYP7B1;PAX2;DNAJC3;VWA8;ZEB1;RP2;KCNMA1;PIGK;RARB;TMEM106B;DEPDC5;PLCB1;RGR;FXN;YAP1;HDAC4;ECHS1;ATL3;BRAT1;COL11A1;ATP1A2;LRP2;SELENOI;ARL13B;CNR1;ERBB4;FLNA;RAB28;MEF2D;FKTN;SNCA;NTRK2;GABRA1;SLC35A3;KCNB1;KIDINS220;GABRA5;AMFR;EYA4;YIPF5;SYT14;LAMB1;DDHD1;KITLG;PNKD;POLR3A;PPP2R2B;SNX14;KLHL7;MSRB3
SYNDROME	0.0024709	0.0783116	EHMT1;HCCS;RBPJ;HOXA13;IFIH1;GHR;GRIP1;SPRED1;LSM11;SALL1;ZIC2;SALL4;TRPS1;TCTN1;TMEM107;HYDIN;EP300;ARID2;KRT6B;CASK;DNAJB13;SOX11;ACSL4;AP3B1;DICER1;LEMD3;ARID1A;ARID1B;PAX2;TGFBR1;ACTA1;SETBP1;RASA2;SOS1;TP53;RNASEH2B;COLEC10;COL11A1;DNAH5;LRP2;PIK3R1;HDAC8;DLL4;DCAF17;ARL13B;RAD21;NSD1;FLNA;PMS1;WNT3;SLC19A2;BRAF;PTPN11;A2ML1;HPS5;GJB2;POLR3A;WT1;NF1;SNAI2;KRAS;BRWD1
SPECIFIC DEVELOPMENTAL DISORDER	0.0035171	0.0967498	MECP2;SETBP1;SMS;SOX11;ARID2;ARID1A;ARID1B;FOXP2
HEPATOBILIARY DISEASE	0.0035224	0.0967498	RB1;NOTCH2;JAG1;AMACR;ATP8B1;MYO5B;NF1;BRAF;KRAS;ARID1A;CYP7B1;TP53
EPISODIC ATAXIA	0.0043896	0.1130336	FGF14;SLC1A3;CACNA1A
GASTROINTESTINAL SYSTEM DISEASE	0.0055301	0.1195355	RB1;NOTCH2;SLC22A5;JAG1;ATP8B1;WDR72;BRAF;NRG1;ARID1A;CYP7B1;AMACR;GDNF;APC;MYO5B;NF1;FLNA;KRAS;ITGB6;TP53;ODAPH;PTPN2
COFFIN-SIRIS SYNDROME	0.0055806	0.1195355	SOX11;ARID1A;ARID2;ARID1B
MONOGENIC DISEASE	0.0056676	0.1195355	F13A1;ANTXR2;HOXA13;GRIP1;LSM11;SALL1;SALL4;LAMP2;SACS;EP300;HMCN1;SLC12A6;ARID2;KRT6B;SCN1A;CACNA2D1;CASK;DNAJB13;SOX11;ACSL4;DICER1;LEMD3;FOXP2;STIM1;SMS;ANKS6;ADAM9;TP53;ALDH7A1;GNE;FBN2;NOTCH2;RNASEH2B;MAOA;SDC2;TSHZ1;CACNA1A;PIK3R1;ABHD5;C2;ATXN1;ATXN7;MAP2;RAD21;INPP5K;SLC19A2;SMAD2;KL;SRD5A3;FZD6;DCDC2;BRAF;A2ML1;HPS5;PUM1;GJB2;FGF14;MYO5B;NF1;SNAI2;TGFBI;BRWD1;TECPR2;RYR2;HEXB;HCCS;BICD2;MECP2;GHR;IFIH1;SPRED1;TRPS1;TCTN1;TMEM107;HYDIN;APPL1;FGG;VPS13A;AP3B1;CYP7B1;ARID1A;ANO5;ARID1B;PAX2;TGFBR1;ACTA1;F9;SETBP1;RASA2;DEPDC5;SOS1;FXN;COLEC10;COL11A1;DNAH5;LRP2;HDAC8;DCAF17;ARL13B;NSD1;FLNA;RAB28;PMS1;FKTN;PEX16;KCNB1;PTPN11;PEX13;ACVR2B;PNKD;DPY19L2;POLR3A;MLPH;APC;WT1;PPP2R2B;SNX14;KRAS

AUTOSOMAL GENETIC DISEASE	0.0062749	0.1195355	HEXB;F13A1;ANTXR2;HOXA13;BICD2;GHR;IFIH1;GRIP1;SPRED1;LSM11;SALL1;SALL4;TRPS1;SACS;EP300;HMCN1;SLC12A6;ARID2;KRT6B;APPL1;SCN1A;FGG;VPS13A;SOX11;AP3B1;DICER1;LEMD3;CYP7B1;ARID1A;ANO5;ARID1B;FOXP2;PAX2;TGFBR1;ACTA1;F9;SETBP1;STIM1;ANKS6;DEPDC5;FXN;TP53;ALDH7A1;GNE;FBN2;NOTCH2;RNASEH2B;COLEC10;SDC2;DNAH5;TSHZ1;CACNA1A;LRP2;PIK3R1;C2;DCAF17;ATXN1;ATXN7;MAP2;RAD21;NSD1;FLNA;PMS1;SLC19A2;FKTN;PEX16;SMAD2;KL;KCNB1;FZD6;DCDC2;BRAF;PTPN11;HPS5;PUM1;PEX13;ACVR2B;PNKD;GJB2;DPY19L2;FGF14;POLR3A;MLPH;APC;WT1;PPP2R2B;SNX14;MYO5B;NF1;SNAI2;TGFBI;TECPR2
RESPIRATORY SYSTEM CANCER	0.0063829	0.1195355	RB1;NF1;MAP3K8;BRAF;NRG1;KRAS;TP53
LUNG CANCER	0.0063829	0.1195355	RB1;NF1;MAP3K8;BRAF;NRG1;KRAS;TP53
DISEASE OF ANATOMICAL ENTITY	0.0072604	0.1271926	RB1;RPL31;COL12A1;RBPJ;ANTXR2;SLC4A4;ANTXR1;PDK3;HMCN1;SLC12A6;SCN1A;CUBN;CACNA2D1;WDR72;ACSL4;RUNX1;SFRP4;NHLRC2;TP53;FREM1;ALDH7A1;GNE;SLC22A5;IGSF1;GATA3;PIK3R1;PCDH19;C9ORF72;NCAPH;C2;PKHD1;CYP27B1;GRIN2A;SPAST;MAP2;ATP6V0A2;INPP5K;ATG7;SCN3A;BARD1;MSR1;CDK19;JAG1;DTNA;FZD6;BRAF;PUM1;GDF5;AIMP1;FAM111A;LACC1;CCDC88A;GJB2;AGO1;MYO5B;TNFSF4;ACER3;SNAI2;TECPR2;ODAPH;DENND1B;GABRB2;RYR2;HEXB;SLC7A14;KIF14;HCCS;GRIK2;SLC6A1;PHF6;JPH1;MECP2;LFNG;MED11;PCLO;SPTLC2;TRIM2;TCTN1;TET2;PAX6;CYP7B1;ANO5;PAX2;RHOB;VWA8;ZEB1;F9;KCNMA1;PIGK;RARB;TMEM106B;PLIN1;DEPDC5;PLCB1;RGR;YAP1;ECHS1;ATL3;STX17;COL11A1;FLNA;CTNNA3;MEF2D;GABRA1;KCNB1;KIDINS220;GABRA5;YIPF5;KITLG;DPY19L2;WT1;SNX14;KLHL7;MSRB3;FBXO28;F13A1;RORA;PRDM1;AHR;NR3C1;TNF;CDC14A;IGF1R;GRIP1;SMCHD1;SALL1;POFUT1;SALL4;SACS;EP300;MAP3K8;DENND5A;MAP3K7;KPNA3;KRT6B;CD96;KCNH5;ATP1B1;CACNB4;STIM1;MSH4;ANKS6;ADAM9;ROR1;IL6ST;FBN2;NOTCH2;GRIA2;CALCB;KMT2A;IREB2;AK2;CACNA1A;SLC1A3;ITPR3;ABHD5;PRPF8;FOXO1;MTM1;ATXN1;IRAK1;ATXN7;BAG3;KL;TOR1A;FANCM;SRD5A2;ATP8B1;SRD5A3;CRIM1;DCDC2;NEB;PTPRD;FGF14;AMACR;GDNF;GNAQ;CAPRIN1;DMXL2;COL5A2;NF1;TUBGCP4;TGFBI;MYD88;GSK3B;CELF2;HSPB8;FHL1;DOCK7;PTPN22;BICD2;GMPPB;ZIC2;GRM7;ZIC1;GATAD1;TMEM107;ITGB6;ZNF644;ATP7A;RUBCN;PDCD6IP;DCC;VPS13C;FGG;VPS13A;NRG1;ETV1;TENT5A;ARID1A;TGFBR1;DNAJC3;ACTA1;RP2;TRAF6;RBM20;FXN;HDAC4;BRAT1;ATP1A2;NDNF;LRP2;SELENOI;ACAN;DCAF17;ARL13B;CNR1;ERBB4;MXI1;RAB28;FKTN;SNCA;NTRK2;SLC35A3;AMFR;EYA4;SPRY4;SYT14;LAMB1;ATP2B1;DDHD1;EPOR;PNKD;POLR3A;MLPH;APC;PPP2R2B;KRAS;PTPN2
COLOBOMA	0.0074092	0.1271926	YAP1;SRD5A3;CRIM1;HCCS;PAX6;LRP2;PAX2;GRIP1;FAM111A;SALL1;ZIC2;SALL4;RARB;FREM1
BILIARY TRACT DISEASE	0.0090775	0.1377475	RB1;AMACR;ATP8B1;MYO5B;NF1;BRAF;KRAS;ARID1A;CYP7B1;TP53
CEREBELLAR DISEASE	0.0090775	0.1377475	ATXN1;FGF14;ATXN7;PPP2R2B;SNX14;ZIC1;SACS;CACNA1A;PUM1;FXN
BILE DUCT DISEASE	0.0090775	0.1377475	RB1;AMACR;ATP8B1;MYO5B;NF1;BRAF;KRAS;ARID1A;CYP7B1;TP53
INTELLECTUAL DISABILITY	0.0093614	0.1377475	MECP2;SETBP1;SMS;SOX11;ARID2;ARID1A;ARID1B
HEREDITARY ATAXIA	0.0103277	0.1467247	ATXN1;FGF14;ATXN7;PPP2R2B;SNX14;SACS;SLC1A3;CACNA1A;PUM1;FXN
AUTOSOMAL DOMINANT INTELLECTUAL DEVELOPME	0.0110814	0.1521846	SETBP1;SOX11;ARID1A;ARID2;ARID1B
MALE REPRODUCTIVE SYSTEM DISEASE	0.0119021	0.1581833	MSR1;DPY19L2;SRD5A2;WT1;MXI1;EP300;LRP2;TGFBR1

GENETIC DISEASE	0.0125399	0.1614517	F13A1;ANTXR2;HOXA13;GRIP1;SMCHD1;SALL1;LSM11;SALL4;LAMP2;SACS;EP300;SLC39A8;HMCN1;SLC12A6;ARID2;KRT6B;SCN1A;CACNA2D1;DNAJB13;CASK;SOX11;ACSL4;CHD1L;DICER1;LEMD3;FOXP2;STIM1;SMS;ANKS6;ADAM9;TP53;ALDH7A1;GNE;FBN2;NOTCH2;RNASEH2B;SLC22A5;MAOA;SDC2;TSHZ1;CACNA1A;ITPR3;PIK3R1;ABHD5;C2;ATXN1;ATXN7;MAP2;RAD21;ARSK;INPP5K;SLC19A2;SMAD2;KL;SRD5A3;FZD6;DCDC2;PHKB;BRAF;A2ML1;HPS5;PUM1;ARHGAP32;GJB2;HAL;FGF14;AMACR;NDUFAF6;MYO5B;DMXL2;CALU;NF1;NDUFAF3;SNAI2;TGFBI;BRWD1;TECPR2;GSK3B;RYR2;MOCS2;FASTKD2;HEXB;MIA3;MTR;PTPN22;HCCS;BICD2;MECP2;GHR;IFIH1;SPRED1;TRPS1;TCTN1;DBT;TMEM107;HYDIN;IDS;ATP7A;AASS;APPL1;FGG;VPS13A;PAX6;AP3B1;CYP7B1;ARID1A;ANO5;ARID1B;PAX2;TGFBR1;DNAJC3;ACTA1;SLC25A15;F9;SETBP1;RASA2;NDUFS1;L2HGDH;DEPDC5;SOS1;FXN;HDAC4;COLEC10;ECHS1;DNAH5;COL11A1;NDNF;LRP2;PPM1K;HDAC8;DCAF17;ARL13B;NSD1;FLNA;RAB28;SH2B3;PMS1;FKTN;PEX16;TRMT10A;KCNB1;YIPF5;SPRY4;PTPN11;PEX13;ACVR2B;PNKD;POLR3A;MLPH;DPY19L2;APC;WT1;PPP2R2B;SNX14;KRAS
BONE DETERIORATION DISEASE	0.0156847	0.1846315	ACAN;CYP27B1;COL11A1;IGF1R
DEGENERATIVE DISC DISEASE	0.0156847	0.1846315	ACAN;CYP27B1;COL11A1;IGF1R
BONE STRUCTURE DISEASE	0.0156847	0.1846315	ACAN;CYP27B1;COL11A1;IGF1R
URINARY BLADDER CANCER	0.0170466	0.1908654	RB1;KMT2A;EP300;ARID1A;TP53;RHOB
MIGRAINE	0.0175715	0.1908654	YAP1;CACNA1A;ATP1A2;MEF2D;SCN1A
CEREBELLAR ATAXIA	0.0176040	0.1908654	ATXN1;FGF14;ATXN7;PPP2R2B;SNX14;SACS;CACNA1A;PUM1;FXN
ORGAN SYSTEM CANCER	0.0193598	0.1971454	RB1;CALCB;KMT2A;LRP2;MXI1;EP300;MAP3K8;RUNX1T1;BARD1;MSR1;SRD5A2;TET2;BRAF;ETV1;NRG1;ARID1A;TGFBR1;RUNX1;RHOB;STIM1;WT1;NF1;KRAS;TP53;MYD88
AICARDI-GOUTIERES SYNDROME	0.0205126	0.1971454	IFIH1;RNASEH2B;LSM11
ARTHRITIS	0.0208543	0.1971454	COL11A1;PTPN22;PRDM1;GATA3;RBPJ;TNF;GDF5;RUNX1;LACC1;IRAK1;TRAF6;IL6ST;PTPN2
MALE REPRODUCTIVE ORGAN CANCER	0.0210543	0.1971454	MSR1;SRD5A2;WT1;MXI1;EP300;LRP2;TGFBR1
PROSTATE DISEASE	0.0210543	0.1971454	MSR1;SRD5A2;WT1;MXI1;EP300;LRP2;TGFBR1
PROSTATE CANCER	0.0210543	0.1971454	MSR1;SRD5A2;WT1;MXI1;EP300;LRP2;TGFBR1
RHEUMATOID ARTHRITIS	0.0232766	0.2125396	LACC1;IRAK1;TRAF6;PTPN22;PRDM1;GATA3;RBPJ;IL6ST;TNF;PTPN2;RUNX1
BLADDER DISEASE	0.0239054	0.2125396	RB1;KMT2A;EP300;ARID1A;TP53;RHOB
URINARY SYSTEM CANCER	0.0242460	0.2125396	RB1;KMT2A;WT1;EP300;ARID1A;TP53;RHOB
REPRODUCTIVE ORGAN CANCER	0.0249101	0.2138121	BARD1;MSR1;SRD5A2;WT1;MXI1;EP300;LRP2;TP53;TGFBR1
REPRODUCTIVE SYSTEM DISEASE	0.0258824	0.2176234	BARD1;YAP1;MSR1;SRD5A2;FANCM;LRP2;TGFBR1;DPY19L2;ERBB4;WT1;MSH4;MXI1;EP300;TP53
URINARY SYSTEM DISEASE	0.0335134	0.2760311	RB1;KL;KMT2A;DCDC2;ARID1A;PAX2;RHOB;PKHD1;GRIP1;CYP27B1;SALL1;WT1;EP300;ANKS6;TP53;FREM1
BONE DISEASE	0.0341689	0.2760311	NOTCH2;COL11A1;PTPN22;GATA3;PRDM1;RBPJ;TNF;IGF1R;LFNG;CYP27B1;ACAN;IRAK1;ZIC1;FLNA;MAP3K7;CD96;TENT5A;GDF5;ANO5;TGFBR1;RUNX1;SFRP4;LACC1;TRAF6;IL6ST;FREM1;PTPN2
PERVASIVE DEVELOPMENTAL DISORDER	0.0357682	0.2780475	KDM5A;MECP2;KDM5B;ASXL3;DYRK1A;ARID1B;SYN2
AUTISM SPECTRUM DISORDER	0.0357682	0.2780475	KDM5A;MECP2;KDM5B;ASXL3;DYRK1A;ARID1B;SYN2
CORE BINDING FACTOR ACUTE MYELOID LEUKEMIA	0.0390132	0.2976569	KRAS;RUNX1;RUNX1T1
CAKUT	0.0405473	0.3029677	GRIP1;SALL1;FREM1;PAX2

AUTOSOMAL DOMINANT CEREBELLAR ATAXIA	0.0451759	0.3029677	ATXN1;FGF14;ATXN7;PPP2R2B;SNX14;CACNA1A;PUM1
NEURODEGENERATIVE DISEASE	0.0472137	0.3029677	GSK3B;ECHS1;HEXB;HSPB8;IREB2;SLC1A3;CACNA1A;ITPR3;C9ORF72;BICD2;SELENOI;MED11;ATXN1;SPAST;PCLO;SPTLC2;ATXN7;ERBB4;CNR1;TRIM2;SACS;PDK3;ATP7A;SLC12A6;ATG7;KPNA3;SNCA;RUBCN;JAG1;KIDINS220;VPS13C;AMFR;VPS13A;SYT14;DDHD1;PUM1;CYP7B1;DNAJC3;CCDC88A;FGF14;PPP2R2B;SNX14;CAPRIN1;TMEM106B;NHLRC2;FXN;TECPR2
HEPATOBILIARY SYSTEM CANCER	0.0484402	0.3029677	RB1;NF1;BRAF;KRAS;ARID1A;TP53
BILE DUCT ADENOCARCINOMA	0.0484402	0.3029677	RB1;NF1;BRAF;KRAS;ARID1A;TP53
BILIARY TRACT CANCER	0.0484402	0.3029677	RB1;NF1;BRAF;KRAS;ARID1A;TP53
BILE DUCT CARCINOMA	0.0484402	0.3029677	RB1;NF1;BRAF;KRAS;ARID1A;TP53
CHOLANGIOCARCINOMA	0.0484402	0.3029677	RB1;NF1;BRAF;KRAS;ARID1A;TP53
BILE DUCT CANCER	0.0484402	0.3029677	RB1;NF1;BRAF;KRAS;ARID1A;TP53
ADENOCARCINOMA	0.0484402	0.3029677	RB1;NF1;BRAF;KRAS;ARID1A;TP53
BONE INFLAMMATION DISEASE	0.0485336	0.3029677	COL11A1;PTPN22;PRDM1;GATA3;RBPJ;TNF;GDF5;RUNX1;LACC1;IRAK1;TRAF6;IL6ST;PTPN2
DIABETES MELLITUS	0.0485336	0.3029677	GSK3B;TRMT10A;YIPF5;ITPR3;PTPN22;MIA3;IFIH1;DNAJC3;DCAF17;DMXL2;SH2B3;SLC19A2;APPL1

ARCHS4 tissues

Term	P-value	Adjusted P-	Genes
NEURONAL EPITHELIUM	1,60E-10	1,72E-09	TMEM41B;NEXMIF;ANKRD20A1;RBPJ;ANKRD20A2;ANTXR1;PPAT;DPYSL3;SOX6;NCKAP5;SRGAP2B;SCN1A;ANKS1B;SOX5;ARL10;EPHA5;WDHD1;CXADR;IAH1;TSPYL1;CACNA2D1;SLC6A15;RFX3;PRKCA;SOX11;ANK3;BCAN;DAAM1;ADGRB3;PRKD3;RUFY3;RFX4;ZNF711;NHLRC2;TP53;ALDH7A1;ASTN1;PFN2;SET;PCDH10;DCUN1D4;HSPA4L;CDCA7;STC1;PCDH19;C9ORF72;NCAPH;GJC1;ADH4;HMGXB4;MAP2;TSPAN6;RAD21;MIER1;GPM6B;BARD1;JAG1;DTNA;CADM1;CADM2;BCL11A;RAB3IP;PUM1;CCDC88A;OCLN;MEX3A;AGO1;CALU;ZNF135;SETD5;ZNF253;ZNF493;NCAN;KIF14;TMEM182;SLC6A1;PHF6;PCSK5;ROBO1;DMBX1;TMEM47;SYNCRIP;EPM2AIP1;NHSL1;RSRC1;TRIM2;TCTN1;GSG1;DLGAP1;AASS;RAB8B;RMI1;ZNF480;STRBP;TET1;PAX6;MTSS1;ZDHHC15;ZEB1;MMP16;AEBP2;HNRNPH1;TMEM33;CNEP1R1;MTF2;TMEM106B;ZNF117;L2HGDH;LRRC3B;RGR;ANGPTL1;YAP1;AMER2;PHLPP1;FAXC;COL11A1;MIPOL1;FAM184A;UBN2;KIAA1841;ZNF224;GPR155;AASDHPPT;RUNX1T1;SRSF12;GINS1;RFTN2;MYEF2;KCNB1;SORT1;KIDINS220;CSNK1A1;USP9Y;LSAMP;SORBS1;KBTBD6;DPY19L2;NFIA;ABI2;NFIB;ETNK1;KLHL7;YIPF6;TACC2;FRYL;ZNF333;NEUROG2;ZNF695;ZNF451;DENND5B;FAM13C;CCDC169;RASEF;ZBTB20;ARID4B;BZW1;IGF1R;SALL1;SALL4;SACS;ARID2;ARHGEF40;PPFIA2;GAS2L3;SLC38A1;ZNF680;SEMA6A;TMEM178B;SEMA6D;TTC7B;CASK;PIAS2;FOXP2;FAM227A;TERB2;RAB30;MELK;CACNB4;TBL1XR1;ADAM9;PHIP;AMPH;ANKRD40;SREK1;SRSF6;ANKRD17;GRIA2;PSMD12;KMT2A;RHOBTB3;CACNA1A;SLC1A3;ILDR2;NREP;PDS5B;GPATCH2L;SPATA6;RTN4;CAMSAP2;KCNV1;NEUROD4;FUT9;CNTNAP3B;ECT2;SRGAP1;LONRF2;MYH10;GRIA3;WNT3;ZNF662;SPECC1;ZBTB18;SMAD2;CBX5;POU2F1;GGT6;FANCM;CCDC14;MARCH6;SMAD5;DCDC1;PTPRD;MOB1B;UBE2W;LSM8;ARHGAP32;LRCH2;LIN28B;DLG1;PAN3;REEP3;NF1;MDM4;TUBGCP4;MAP3K13;BRWD1;KDM5A;KDM5B;USP37;CELF2;LRRC74B;DOCK7;ELAVL4;PSIP1;CHD1;ELAVL2;ELAVL3;SENP7;ZIC2;LRRTM3;SUMO1;TRPS1;ZNF649;PPP6R3;ZIC1;DBT;LRRTM2;HYDIN;ITGB8;CEP170;ZNF644;ZNF521;ADAMTS6;USP47;HMGCS1;CCDC34;DCC;H2AFZ;ETV1;OPRM1;ARID1A;ARID1B;PTBP2;TGFBR1;MMS22L;RBL1;DBF4;CREB1;SETBP1;PALLD;ZNF516;CDH12;LCORL;HDAC2;PLEKHH2;TTK;ATP1A2;CXXC4;PPM1E;TOB1;JAKMIP2;CNR1;ERBB4;ZNF624;TMEM209;SLIT2;PCBD2;E2F5;E2F7;NLGN4Y;MCOLN3;NEGR1;PCDH7;EYA4;KIAA1549L;SYT14;ZIC5;DDHD1;SOD2;ACVR2B;PTPN14;ACVR2A;DLK1;CTCFL;MAPK10;APC;SYT11;PPP2R2B;NOVA1;ASXL3;PI15;TRIM36;PAXBP1;PTPN4
PREFRONTAL CORTEX	1,52E-02	8,19E-01	FRMPD4;HHIP;NEXMIF;TRIL;ANKRD20A1;ANKRD20A2;SOGA1;PREX2;DPYSL3;SOX6;NCKAP5;SRGAP2B;SCN1A;ANKS1B;SOX5;ARL10;EPHA5;CXADR;UNC13A;CACNA2D1;SLC6A15;RFX3;PRKCA;SOX11;UNC5D;ANK3;GABRG1;BCAN;DAAM1;ADGRB3;RUFY3;RFX4;ZNF711;ALDH7A1;ASTN1;PFN2;NECAB1;PCDH10;HSPA4L;PCDH19;GJC1;ADH4;GRIN2A;MAP2;TSPAN7;KCNN3;SCN3A;GPM6B;NTNG1;EGR1;DTNA;CADM1;CADM2;BCL11A;SS18L1;ST8SIA3;RAB3IP;SLC16A14;CCDC88A;PEX5L;MEX3A;ANKRD20A4;XKR4;ZNF135;CPEB4;PCSK2;GABRB2;RYR2;ZNF493;NCAN;SLC7A14;GRIK1;TMEM182;GRIK2;SIPA1L2;SLC6A1;ROBO1;EPM2AIP1;NHSL1;PCLO;TRIM2;TCTN1;GSG1;DLGAP1;ZNF365;ZNF480;STRBP;PAX6;SYN2;PRSS12;MTSS1;ZDHHC15;RHOB;ZEB1;MMP16;CCKBR;HNRNPH1;L2HGDH;RGS7BP;LRRC3B;PLCB1;RGR;PDZRN4;AMER2;FAXC;COL11A1;PLPPR4;GDPD1;NAPG;MIPOL1;FAM184A;UBN2;KIAA1841;RICTOR;ZNF224;GPR155;RUNX1T1;SRSF12;P2RY12;RFTN2;GABRA1;MYEF2;MOG;KCNB1;KIDINS220;GABRA5;GABRA4;LSAMP;SORBS1;NMNAT2;KITLG;KBTBD6;NFIA;ABI2;NFIB;ETNK1;KLHL7;CAMK4;VWC2;TACC2;ZNF333;NEUROG2;ZNF695;CEP126;DENND5B;FAM13C;CCDC169;RASEF;KLHL32;ZBTB20;ARID4B;CELSR3;SLC8A1;IGF1R;GRIP1;SALL1;SACS;ARID2;PPFIA2;GUCY1A2;KCNH5;SEMA6A;TMEM178B;SEMA6D;TTC7B;CASK;ATP1B1;PIAS2;FAM227A;OLFM1;CACNB4;TBL1XR1;KIAA2026;SRSF6;DGKI;GRIA2;KMT2A;SLC1A1;CACNA1A;SLC1A3;ILDR2;NREP;PDS5B;SPATA6;LRRC55;RTN4;MCHR2;CAMSAP2;KCNV1;CDH20;FUT9;CNTNAP3B;SRGAP1;RAB6B;LONRF2;MYH10;GRIA3;WNT3;ZNF662;SPECC1;ZBTB18;POU2F1;GGT6;CCDC14;MARCH6;PTPRD;ARHGAP32;LRCH2;FGF14;DMXL2;CNTN1;NF1;TUBGCP4;MAP3K13;BRWD1;THRB;CELF2;LRRC74B;DOCK7;ELAVL4;PSIP1;RERG;HAPLN1;ELAVL2;ELAVL3;GRM3;SENP7;GRM5;CLEC1A;ZIC2;GRM7;LRRTM3;ZIC1;GDAP1L1;LRRTM2;HYDIN;ITGB8;CEP170;USP47;TRPC5;HMGCS1;DCC;GPX6;NRG1;ETV1;OPRM1;ARID1B;PTBP2;MMS22L;SETBP1;RASA1;CDH12;PPP1R12B;SOS1;COLEC12;PLEKHH2;CAMK2A;ATP1A2;PPM1K;CXXC4;PPM1E;TOB1;JAKMIP2;FBXL20;NWD1;HECTD2;CNR1;ERBB4;SLITRK4;REPS2;ZNF500;CACNG2;NTRK2;NEGR1;PCDH7;KIAA1549L;SYT14;CCK;DDHD1;SOD2;ACVR2B;ACVR2A;CTCFL;MAPK10;KLF7;APC;SYT11;PPP2R2B;NOVA1;ASXL3;TRIM36;PTPN4;MDGA2;KCNK2

FETAL BRAIN CORTEX	3,48E+06	1,25E+08	TMEM41B;NEXMIF;EHMT1;ANKRD20A1;RBPJ;SOGA1;DPYSL3;FAM122C;EPC1;NCKAP5;SRGAP2B;ANKS1B;SOX5;ARL10;EPHA5;CXADR;IAH1;TSPYL1;CACNA2D1;SLC6A15;RC3H1;PRKCA;SOX11;UNC5D;ANK3;THAP5;DAAM1;ADGRB3;RUFY3;RFX4;ZNF711;ALDH7A1;ASTN1;PFN2;SET;DCUN1D3;DCUN1D4;HSPA4L;C9ORF72;SMIM8;GJC1;INPP5B;ADH4;MAP2;ATP6V0A2;KCNN3;SCN3A;GPM6B;CADM1;BCL11A;MEX3D;PHC3;CCDC88A;MEX3A;ANKRD20A4;NDUFAF6;AGO1;MYO5B;PPP1R1C;XKR4;ZNF135;CPEB4;ZNF253;ZNF493;NCAN;SLC7A14;TMEM182;GRIK2;ROBO1;LETM2;DMBX1;NHSL1;PCLO;TRIM2;TCTN1;DLGAP1;ELOC;AASS;ZYG11A;SLC2A11;ZNF480;STRBP;ZDHHC13;PAX6;MTSS1;ZDHHC15;MMP16;AEBP2;HNRNPH1;TMEM33;MTF2;TMEM106B;ZNF117;L2HGDH;LRRC3B;RGR;AMER2;FAXC;FAM129A;GDPD1;NAPG;MIPOL1;PRAMEF1;UBN2;KIAA1841;RICTOR;ZNF224;GPR155;RUNX1T1;SRSF12;MYEF2;KCNB1;KIDINS220;CSNK1A1;LSAMP;SORBS1;NMNAT2;KBTBD6;NFIA;ABI2;NFIB;ETNK1;KLHL7;TACC2;CCL28;ZNF333;NEUROG2;ZNF695;ZNF451;DENND5B;FAM13C;CCDC169;RASEF;KLHL32;ARID4B;IGF1R;GRIP1;ARID2;PPFIA2;GUCY1A2;SLC38A1;KCNH5;SEMA6A;SEMA6D;TTC7B;MR1;PIAS2;FAM227A;TERB2;CACNB4;TBL1XR1;ANKRD40;SRSF6;STEAP2;GRIA2;DDX5;PSMD12;KMT2A;CACNA1A;NREP;PDS5B;GPATCH2L;LRRC55;CSF2RA;RTN4;KCNV1;NEUROD4;BTBD1;FUT9;CNTNAP3B;SRGAP1;MYH10;GRIA3;WNT3;ZNF662;SPECC1;ZBTB18;SMAD2;CBX5;GGT6;CCDC14;A2ML1;MARCH6;MARCH3;PTPRD;MOB1B;UBE2W;LRCH2;PAN3;DNAJC10;MDM4;TUBGCP4;MAP3K13;BRWD1;KDM5B;CELF2;LRRC74B;DOCK7;ELAVL4;PSIP1;EFCAB11;ELAVL2;ELAVL3;GRM3;SENP7;SUMO1;ZNF649;GDAP1L1;DBT;LRRTM2;HYDIN;ITGB8;CEP170;SLC37A3;HMGCS1;DCC;GPX6;NRG1;OPRM1;ARID1A;ARID1B;PTBP2;KLF17;MMS22L;SETBP1;LCORL;COLEC12;HDAC2;PPM1K;PPM1E;TOB1;JAKMIP2;GK5;FBXL20;NWD1;CNR1;ZNF506;REPS2;ZNF624;NUP43;PCBD2;RPP14;SNCA;MCOLN3;SLC35A3;KIAA1549L;SYT14;DDHD1;ACVR2B;PTPN14;ACVR2A;CTCFL;MAPK10;KLF7;APC;SYT11;PPP2R2B;NOVA1;ASXL3;TRIM36;PAXBP1;PTPN4;SSBP3
MYOBLAST	1,04E+09	2,81E+10	TMEM41B;HHIP;COL12A1;LDLRAD2;RBPJ;ANTXR1;ALCAM;BCAP29;SRGAP2B;SCN1A;IAH1;TSPYL1;PAQR5;FRS2;GTPBP8;SOX11;ACSL4;ACSL3;DKK1;RUNX1;CPED1;SFRP4;DYNC1LI2;THAP5;FEZ2;TLN2;TP53;BLZF1;PFN2;SET;DCUN1D4;C2CD4A;TWF1;TMTC1;PCDH19;SEC14L2;FXR1;ADH4;TSPAN6;SMIM11B;MIER1;DPH6;CGA;HOXC8;SNX6;MCTS1;EGR1;YES1;MEX3D;AIMP1;ASAH2B;TNFSF4;CALU;SNAI2;MRPS17;MOCS2;SLC7A14;OLA1;RAB22A;WDR43;EDA2R;DMBX1;ADAMTSL1;TMEM47;SYNCRIP;GLIPR1;NHSL1;GSG1;TNFAIP8L3;SDF2;RRAS2;AP3B1;ZDHHC15;FAM210A;ZEB1;BDH2;AEBP2;PSMA1;HNRNPH1;PSMA2;TMEM33;CNEP1R1;PIGK;STRAP;RGR;UMPS;ERGIC2;YAP1;ECHS1;ATL3;SLC20A1;SYNPO2;COL11A1;SRP72;PRAMEF1;KIAA1841;DTD1;KCNB1;SORT1;CSNK1A1;CAVIN2;PBDC1;YIPF5;MPP5;HIPK3;ITCH;FABP3;NFIA;NFIB;NFIC;STK17B;MSRB3;TACC2;CCL28;ZNF451;CEP126;CCDC169;WWC2;F13A1;ZBTB20;WBP1L;HNMT;ALDH1L2;FGF2;BZW1;SLC8A1;RGS4;LAMP2;SACS;KPNA4;KMT5A;ARID2;WASHC3;KIRREL1;DNAJB13;FOXP1;FAM227A;RAB30;TBL1XR1;ADAM9;ROR1;ANKRD40;SELENOT;IL6ST;SRSF6;DGKI;FBN2;NOTCH2;PSMD12;RHOBTB3;NREP;FOXO3;RTN4;MCHR2;CAMSAP2;KCNV1;BAG3;CNTNAP3B;ABL2;TBC1D12;SRGAP1;SLC38A2;WNT3;SMAD2;ATP8B1;CRIM1;NEB;A2ML1;SMAD5;DCDC1;MARCH3;UBE2W;DLG1;FGF14;CNIH1;REEP5;REEP3;COL5A2;UBA3;TGFBI;CNIH4;PBOV1;NFE2L2;LRRC74B;CBWD3;ADAMTS5;GRM7;SIX4;SUMO1;CASP3;PPP6R3;DBT;PHACTR2;EIF3CL;ITGB6;METTL15;ADAMTS6;NCK1;HMGCS1;COMMD10;GPX6;GPX8;NRG1;OPRM1;TGFBR1;DNAJC3;ACTA1;ASPH;PALLD;RASA1;CHMP3;USP40;B4GALT4;CHMP5;PLEKHH2;ATP10D;TAF9;TOB1;ZBTB41;ACAN;NWD1;PDLIM3;ELMSAN1;DNAJB4;NAA25;TMEM209;NUP43;APOPT1;S1PR3;PCBD2;PDLIM5;DCAF13;FKTN;SEPT10;MCOLN3;NEGR1;GALNT1;EYA4;LAMB1;ATP2B1;PTPN12;PTPN14;SHCBP1;CTCFL;KLF7;MLPH;DNAJA2;PDE7B;KCNK2;RECK

CINGULATE GYRUS	5,06E+08	1,09E+11	CHIC1;FRMPD4;HHIP;NEXMIF;TRIL;ANKRD20A2;RXFP1;PREX2;PDK3;SCN1A;ANKS1B;SOX5;EPHA5;UNC13A;PRKCB;CACNA2D1;PRKCE;SLC6A15;RFX3;UNC5D;ANK3;FNDC9;GABRG1;BCAN;ADGRB3;TLN2;ASTN1;TSPYL5;KIAA1211L;NECAB1;TSHZ3;PCDH10;MAOA;HSPA4L;TMTC1;PIK3R1;PCDH19;GRIN2A;MAP2;TSPAN7;PIP5K1B;KCNN3;PPARGC1A;SCN3A;GPM6B;JAG1;DTNA;CADM2;ST8SIA3;NR1D2;SLC16A14;HRNR;PEX5L;TECPR2;PCSK2;GABRB2;RYR2;NCAN;BHLHE41;SLC7A14;GRIK1;GRIK2;SLC6A1;JPH1;TMEM47;PCLO;TRIM2;SH3BGRL2;IDS;DLGAP1;ZNF365;APOBEC4;CYP7B1;SYN2;ANO5;CDYL2;RHOB;SLC7A4;MBLAC2;MMP16;CCKBR;PLSCR4;KCNMA1;RARB;RGS7BP;LRRC3B;PLCB1;PDZRN4;AMER2;SYNPO2;FAXC;PLPPR4;TMEM74;PURA;FAM184A;MGAT3;CTNNA3;MEF2D;RUNX1T1;P2RY12;GABRA1;MOG;KCNB1;SORT1;GABRA5;GABRA4;USP9Y;LSAMP;FMO2;SORBS1;NMNAT2;KITLG;FABP3;TMEM56;CAMK4;VWC2;IL17D;ZNF354B;CEP126;FAM13C;GALNT18;KLHL32;CELSR3;SLC8A1;AMOT;RGS4;ARHGAP44;GRIP1;RGS5;SALL1;ENPP4;NUDT12;PPFIA2;GUCY1A2;KCNH5;TMEM178B;ANKRD46;FBXW7;LMO4;SEMA6D;ATP1B1;CACNB1;OLFM1;ADCYAP1;CACNB4;AMPH;STEAP2;DGKI;GRIA2;GPR88;SLC1A1;SLC1A3;KLHL11;ILDR2;MCHR2;CAMSAP2;KCNV1;ARHGAP20;CDH20;FUT9;NACC2;RAB6B;LONRF2;GRIA3;KL;FAM199X;ESRRG;PTPRD;ARHGAP32;LRCH2;PTPRB;FGF14;HOOK1;CNTN1;ZNF777;CNTNAP3;THRB;MCTP1;HSPB8;PTPN20;ELAVL4;CLEC14A;HAPLN1;ELAVL2;ELAVL3;GRM3;SEPT8;GRM5;GRM7;LRRTM3;GRM8;ZIC1;GDAP1L1;LRRTM2;CEP170B;NPTN;SPX;UPP2;TRPC5;DCC;GPX6;LANCL1;ETV1;OPRM1;RTN4RL1;CDH12;SOWAHA;SSX2IP;HLF;PALM2;CAMK2A;SEMA3G;ATP1A2;NDNF;LRP2;CXXC4;PPM1E;DLL4;SV2C;HECTD2;CNR1;ERBB4;SLITRK4;REPS2;SLIT2;CACNG2;SNCA;NLGN4Y;NTRK2;NEGR1;PCDH7;KIAA1549L;CCK;ATP2B1;MAPK10;APC;SYT11;PPP2R2B;NOVA1;SYT10;ASXL3;BRMS1L;PI15;TRIM36;MDGA2;KCNK2
PANCREATIC ISLET	1,31E+11	2,37E+12	EIF4A2;TMEM41B;RPS6KA6;RINL;ALCAM;RASSF6;SEC62;SOX6;ANKS1B;UNC13A;IAH1;TSPYL1;CACNA2D1;ISL1;THAP5;DAAM1;RUFY3;GNE;TSPYL5;PFN2;SDC2;IGSF1;MYCBP2;HPN;HSPA4L;C2CD4A;INPP5B;ADH4;MAP2;SMIM11B;TSPAN7;MIER1;HAO1;DPH6;NTNG1;MSR1;EGR1;DTNA;CADM1;ABCA5;ST8SIA3;RAB3IP;PHC3;OCLN;XKR4;CPEB4;MRPS17;PCSK2;ZNF253;SLC7A14;GRIK2;RAB22A;LETM2;DMBX1;EPM2AIP1;PCLO;RSRC1;TRIM2;TCTN1;GSG1;MRC1;MUC13;SH3BGRL2;IDS;TNFAIP8L3;CTBS;SLC35G2;PAX6;ANO5;MTSS1;ZDHHC15;SLC7A8;AEBP2;TMEM33;CNEP1R1;KCNMA1;PIGK;TMEM106B;SGK3;L2HGDH;FAXC;MRPL57;NAPG;MIPOL1;PURA;KIAA1841;GPR155;RUNX1T1;MYEF2;KCNB1;CSNK1A1;TMEM176B;ELP4;NMNAT2;TMEM59;ETNK1;SNX14;NFIC;YIPF6;TACC2;CCL28;ZNF333;MPHOSPH6;CEP126;ERO1B;CCDC169;WWC1;RASEF;ZBTB20;DEUP1;IGF1R;RGS4;TMEM266;LAMP2;ZNF208;WASHC3;SNRPN;TMEM178B;TTC7B;ATP1B1;MR1;PIAS2;FOXP2;FOXP1;FAM227A;ZNF90;ADCYAP1;TMX4;CACNB4;TBL1XR1;ANKRD40;SLC25A53;SELENOT;SRSF6;STEAP2;GRIA2;PSMD12;SLC41A2;RHOBTB3;CACNA1A;REG3G;PDS5B;LPP;CSF2RA;RTN4;KCNV1;ADGRG2;COBLL1;HRH4;SRGAP1;LONRF2;MYH10;PDZD8;GRIA3;ZNF662;RAP1GAP2;KL;GGT6;DCDC2;ESRRG;ABHD18;TBCA;MARCH6;DCDC1;MOB1B;UBE2W;NR4A1;FGF14;REEP5;HOOK1;REEP3;CNTN1;MDM4;TUBGCP4;MAP3K13;GCLM;PBOV1;CPM;LRRC74B;CBWD3;ELAVL4;CBWD2;CBWD1;EFCAB11;GHR;DBT;GATAD1;NPTN;ITGB8;PHACTR2;MAN1A1;VPS13C;ETV1;DUSP28;ARID1B;DNAJC3;ASPH;PALLD;ZNF516;CDH12;CHMP3;SOWAHA;SSX2IP;COLEC12;PLEKHH2;CCDC186;PLAG1;DNAH5;GOSR1;PPM1K;CXXC4;TOB1;JAKMIP2;GK5;NWD1;ZNF506;REPS2;TMEM209;PCBD2;PDLIM5;ZNF500;POLR2K;CYB5A;MCOLN3;SLC35A3;CHURC1;MACC1;AMFR;SYT14;SEPT14;DLK1;CTCFL;MAPK10;FAM167A;SYT11;NOVA1;ASXL3

CEREBELLUM	1,84E+11	2,84E+12	PANK3;CCDC89;FRMPD4;NEXMIF;TRIL;COL12A1;PREX2;LURAP1L;DPYSL3;HMCN1;FBXO8;NCKAP5;SCN1A;ANKS1B;SOX5;EPHA5;UNC13A;PAQR5;CACNA2D1;ZFP3;GLCE;SLC6A15;EBF1;FRS2;WDR72;SOX11;UNC5D;ANK3;GABRG1;BCAN;SFRP4;UTS2B;ADGRB3;RUFY3;RFX4;ZNF711;TLN2;FREM1;ASTN1;PCDH10;C7ORF57;PCDH19;SEC14L2;PKHD1;EPB41L5;GRIN2A;MAP2;TSPAN7;PPARGC1A;SCN3A;GPM6B;FZD1;DTNA;ABCA5;CADM2;ST8SIA3;MEX3C;SLC16A14;HRNR;ASAH2B;MEX3A;XKR4;CPEB4;GABRB2;RYR2;NCAN;GRIK1;GRIK2;SIPA1L2;EFCAB7;SLC6A1;ROBO1;PCLO;TRIM2;DLGAP1;APOBEC4;TET1;PAX6;CYP7B1;SYN2;PAX2;TMEFF1;ZEB1;MMP16;KCNMA1;RARB;RGS7BP;LRRC3B;PLCB1;PDZRN4;AMER2;FAXC;COL11A1;PLPPR4;TMEM74;KCNA6;MIPOL1;MGAT3;CTNNA3;RUNX1T1;P2RY12;GABRA1;MYEF2;KIDINS220;GABRA4;DAB2IP;LSAMP;FMO2;NMNAT2;KITLG;NFIB;TMEM56;CAMK4;VWC2;IL17D;ZNF354C;ZNF354B;FAM13C;GALNT18;RASEF;F13A1;ZBTB20;RORA;CELSR3;SLC8A1;GRIP1;SACS;PLXNC1;NUDT12;PPFIA2;GUCY1A2;GAS2L3;KCNH5;ZNF680;SEMA6A;TMEM178B;SEMA6D;FOXP2;OLFM1;ADCYAP1;CACNB4;MSH4;AMPH;DGKI;GRIA2;CALCB;SLC1A1;SLC1A3;KLHL11;ILDR2;CAMSAP2;ARHGAP20;NEUROD4;CDH20;FUT9;RAB6B;LONRF2;GRIA3;ZBTB18;ZNF540;ESRRG;DCDC1;PTPRD;LRCH2;FGF14;IL7;DMXL2;COL5A2;CNTN1;SP5;SLC26A7;ZNF772;PTPN20;ELAVL4;RERG;HAPLN1;ELAVL2;ELAVL3;GRM3;ADAMTS5;CA1;GRM5;CALB1;ZIC2;GRM7;LRRTM3;TRPS1;GRM8;ZIC1;GDAP1L1;LRRTM2;TMEM107;HYDIN;MYBL1;ZNF521;ADAMTS6;TRPC5;DCC;ETV1;SETBP1;CDH12;LCORL;HLF;PALM2;IER5L;TTK;ATP1A2;NDNF;LIN9;LRP2;CXXC4;PPM1E;ZBTB41;PDLIM3;HECTD2;CNR1;ERBB4;SLITRK4;SLIT2;CACNG2;NTRK2;PCDH7;KIAA1549L;SYT14;ZIC5;MAPK10;KLF7;APC;SYT11;PPP2R2B;NOVA1;ASXL3;PI15;TRIM36;MDGA2;KCNK2
HUMAN EMBRYO	1,66E+12	0.0022466	TDRKH;TMEM41B;TRIL;ANTXR1;PREX2;BAIAP2L1;PPAT;DPYSL3;MYOZ3;HMCN1;SEPHS1;WDHD1;STARD4;CXADR;UNC13A;CACNA2D1;SLC6A15;SOX13;WDR72;SOX11;UNC5D;ACSL3;CD2AP;ZNF711;ALDH7A1;PFN2;NECAB1;L3MBTL3;SET;PCDH10;SDC2;IGSF1;DCUN1D4;CDCA7;STC1;NCAPH;GJC1;EPB41L5;MAATS1;HMGXB4;SPAST;TSPAN6;ARSK;DPH6;GPM6B;SLC19A2;BARD1;YES1;DDIAS;CADM2;ST8SIA3;INTS2;LARP4;MEX3D;MEX3C;MEX3A;FUBP3;AGO1;CALU;MRS2;ZNF253;RYR2;FASTKD2;KIF14;CTSV;PHF6;JPH1;WDR43;EDA2R;ROBO1;TMEM47;NHSL1;TRIM2;RBPMS2;SH3BGRL2;DLGAP1;JARID2;AASS;PARP1;RMI1;STRBP;RRAS2;TET1;SIRT1;ANO5;TMEFF1;FAM210A;SLC7A8;MMP16;CCKBR;SDHAF3;MED20;MTF2;SLC29A1;YAP1;FAXC;COL11A1;TMEM74;FAM184A;RUNX1T1;GINS1;GABRA1;MYEF2;GABRA5;SORBS1;NMNAT2;TTC9;FABP3;KLHL7;TRMT6;CENPQ;MPHOSPH6;ZNF695;TMEM167A;WWC1;WWC2;FGF2;CDC14B;GRIP1;SALL1;SALL4;SACS;OIP5;SLC39A1;CARD10;GAS2L3;SLC38A1;KIRREL1;SNRPN;SEMA6A;TMEM178B;CASK;ZNF90;HIC2;OLFM1;MELK;TMEM136;SMS;ANKS6;ROR1;FBN2;PSMD12;CALCB;CHRNA5;SLC1A3;NREP;CAMSAP2;FUT9;G3BP1;RPRD1A;ECT2;MYH10;WNT3;ARHGEF35;CBX5;POU2F1;FANCM;NFXL1;AZIN1;PTPRD;LRCH2;LIN28B;HOOK1;SP4;COL5A2;BRWD3;KDM5B;USP37;MTCL1;PSIP1;NXT2;ELAVL2;SPRED1;CALB1;ZIC2;SIX4;MDK;CASP3;ZNF649;GRM8;EIF3CL;POLK;SMIM15;HSDL1;HMGCS1;CCDC34;DESI2;COMMD10;GPX8;ETV1;PTBP2;TGFBR1;TRDN;ACTA1;DBF4;MCM5;NEMP1;HDAC2;PALM2;TTK;ATP1A2;LIN9;LRP2;PPM1E;MRM2;SELENOI;JAKMIP2;WDCP;SLIT2;E2F5;ADSS;E2F7;DCAF13;NLGN4Y;SEPT10;SPRY4;SYT14;ZIC5;ACVR2B;SHCBP1;PPP2R2B;SYT10;BRMS1L;DNAJA2;RBM41;TRIM36;TRIM37;KRAS;METAP1
CEREBRAL CORTEX	2,23E+11	0.0026783	CHIC1;FRMPD4;HHIP;NEXMIF;TRIL;ANKRD20A1;ANKRD20A2;RXFP1;PREX2;DPYSL3;NCKAP5;SCN1A;ANKS1B;SOX5;EPHA5;UNC13A;CACNA2D1;PRKCE;SLC6A15;UNC5D;ANK3;FNDC9;GABRG1;BCAN;ADGRB3;RUFY3;RFX4;ZNF711;TLN2;ASTN1;PFN2;KIAA1211L;NECAB1;PCDH10;PCDH19;NLK;SEC14L2;MAATS1;GRIN2A;MAP2;TSPAN7;KCNN3;PPARGC1A;SCN3A;GPM6B;NTNG1;DTNA;CADM1;CADM2;ST8SIA3;SLC16A14;CCDC88A;PEX5L;ANKRD20A4;PCSK2;GABRB2;RYR2;NCAN;BHLHE41;GRIK1;GRIK2;SIPA1L2;SLC6A1;JPH1;TMEM47;PCLO;TRIM2;RSPO3;DLGAP1;ZNF365;APOBEC4;PAX6;CYP7B1;SYN2;GNL1;ANO5;CDYL2;MMP16;CCKBR;KCNMA1;RGS7BP;LRRC3B;PLCB1;PDZRN4;AMER2;FAXC;COL11A1;PLPPR4;PURA;FAM184A;MGAT3;CTNNA3;SH3BGR;RFTN2;GABRA1;MOG;GABRA5;GABRA4;LSAMP;SORBS1;NMNAT2;TTC9;NFIA;TMEM56;CAMK4;VWC2;IL17D;CEP126;FAM13C;WWC1;KLHL32;ZBTB20;CELSR3;SLC8A1;AMOT;RGS4;ARHGAP44;RGS5;FGF7;SALL1;PPFIA2;GUCY1A2;KCNH5;TMEM178B;FBXW7;LMO4;SEMA6D;CYS1;CACNB1;OLFM1;ADCYAP1;RAB30;ARMCX1;CACNB4;KIAA2026;ANKS6;AMPH;DGKI;GRIA2;GPR88;SLC1A1;STK39;CACNA1A;SLC1A3;ILDR2;CAMSAP2;SNN;ARHGAP20;CDH20;FUT9;NACC2;RAB6B;LONRF2;GRIA3;WNT3;ZBTB18;ZNF540;ANKRD29;INHBB;PTPRD;ARHGAP32;LRCH2;FGF14;THRB;MTCL1;TCHH;ELAVL4;PSIP1;CLEC14A;ELAVL2;ELAVL3;GRM3;SEPT8;SENP7;GRM5;CALB1;ZIC2;GRM7;LRRTM3;GRM8;ZIC1;GDAP1L1;LRRTM2;CEP170B;HYDIN;SPX;UPP2;CCDC34;DCC;ETV1;TRDN;SETBP1;RTN4RL1;SOWAHA;SSX2IP;HLF;PALM2;CAMK2A;ATP1A2;LRP2;PPM1K;CXXC4;PPM1E;JAKMIP2;NWD1;HECTD2;CNR1;ERBB4;SLITRK4;CACNG2;SNCA;NLGN4Y;NTRK2;PCDH7;KIAA1549L;KCNJ16;CCK;MAPK10;DBNDD2;FAM167A;APC;SYT11;PPP2R2B;NOVA1;ASXL3;FSIP1;MDGA2;KCNK2

MIDBRAIN	8,94E+11	0.0096584	EIF4A2;GMFB;RPL31;WIPF2;NEXMIF;TRIL;CCP110;ANKRD20A1;DCAF7;RPS6KA6;LSM11;DPYSL3;EPC1;SEC62;SOX6;NCKAP5;SCN1A;ARL10;EPHA5;CXADR;EBF1;RFX3;SOX11;ANK3;BCAN;DYNC1LI2;ADGRB3;RUFY3;RFX4;ZNF711;ALDH7A1;ASTN1;PFN2;PCDH10;HSPA4L;ATXN7L3B;PCDH19;GJC1;MAATS1;GRIN2A;MAP2;TSPAN6;TSPAN7;KCNN3;SCN3A;GPM6B;JUN;JAG1;CADM1;CADM2;BCL11A;ST8SIA3;MEX3D;CCDC88A;MEX3A;XKR4;SETD5;GABRB2;LRRC34;NCAN;GRIK2;SLC6A1;TMEM47;EPM2AIP1;PCLO;TRIM2;CNOT6L;RHOB;RNF168;ZEB1;MMP16;GFRAL;L2HGDH;CLDN16;PDZRN4;AMER2;FAXC;PATE1;KCNA6;AKAP4;MIPOL1;CXCR2;RUNX1T1;RFTN2;GABRA1;MYEF2;KIDINS220;LSAMP;NMNAT2;NFIA;ABI2;NFIB;TACC2;NEUROG2;FOXA1;DENND5B;FAM13C;WWC1;ZBTB20;IGF1R;TMEM266;SALL1;CHCHD3;SALL4;ZNF208;MAEL;SLC16A7;ARHGEF40;PPFIA2;GUCY1A2;SEMA6A;TMEM178B;LMO4;SEMA6D;SHISA3;PIAS2;FOXP2;FOXP1;FAM227A;OLFM1;TERB2;SREK1;RAD54L2;SRSF6;GRIA2;CALCRL;KMT2A;CHRNA5;CACNA1A;SLC1A3;ILDR2;NREP;DTX4;LPP;RTN4;CAMSAP2;SNN;NEUROD4;FUT9;SRGAP1;MYH10;SMAD2;ANKRD29;CBX5;POU2F1;HOOK3;TBCA;MARCH6;GVQW2;DCDC1;PTPRD;BMP3;NR4A1;EBPL;MDM4;MAP3K13;GSK3B;DOCK7;CBWD3;ELAVL4;EFCAB11;ELAVL2;ELAVL3;GRM3;SENP7;CA1;GRM5;LRRTM3;GRM8;GDAP1L1;LRRTM2;HYDIN;ITGB8;ZNF521;HMGCS1;CCDC34;DCC;H2AFZ;RPL13A;ARID1A;PTBP2;KLF17;RCN2;SETBP1;SNURF;LUC7L2;COLEC12;HDAC2;CAMK2A;IER5L;ATP1A2;LRP2;PPM1K;CXXC4;PPM1E;JAKMIP2;PDLIM3;SV2C;CNR1;ERBB4;DNAJB4;SLITRK4;ZNF506;MXI1;SLIT2;PDLIM5;CACNG2;NLGN4Y;NTRK2;NEGR1;PCDH7;KIAA1549L;KCNJ16;SYT14;SOD2;ACVR2B;ACVR2A;MAPK10;SYT11;NOVA1;ASXL3;TRIM36;SSBP3
DENTATE GRANULE CELL	0.0024661	0.0242130	CHIC1;CCDC89;FRMPD4;HHIP;NEXMIF;TRIL;ANKRD20A1;ANKRD20A2;LURAP1L;CCDC96;SCN1A;ANKS1B;EPHA5;CXADR;UNC13A;CACNA2D1;PRKCE;SLC6A15;RFX3;UNC5D;ANK3;FNDC9;GABRG1;BCAN;ADGRB3;RUFY3;RFX4;ASTN1;TSPYL5;PFN2;KIAA1211L;NECAB1;ZNF391;IGSF1;C7ORF57;TMTC1;PCDH19;SEC14L2;MAATS1;GRIN2A;MAP2;TSPAN7;PIP5K1B;KCNN3;PPARGC1A;SCN3A;GPM6B;DTNA;CADM2;ST8SIA3;SLC16A14;HRNR;PEX5L;ANKRD20A4;MYO5B;PPP1R1C;FANK1;PCSK2;GABRB2;RYR2;NCAN;BHLHE41;SLC7A14;GRIK2;EFCAB7;SLC6A1;JPH1;PCLO;TRIM2;SH3BGRL2;RSPO3;DLGAP1;TNFAIP8L3;ZNF365;APOBEC4;CYP7B1;DUSP9;SYN2;ANO5;SLC7A4;MBLAC2;MMP16;CCKBR;PLSCR4;KCNMA1;RARB;PLIN1;RGS7BP;CLDN16;LRRC3B;PLCB1;ANGPTL1;PDZRN4;AMER2;SPESP1;FAXC;PLPPR4;PATE1;TMEM74;NAPG;FAM184A;CTNNA3;SH3BGR;P2RY12;GABRA1;GABRA5;GABRA4;LSAMP;NMNAT2;KBTBD3;FABP3;NFIA;NFIB;VWC2;IL17D;NEUROG2;FAM13C;GALNT18;KLHL32;ZBTB20;DEUP1;RGS4;RGS5;FGF7;MAEL;PPFIA2;GUCY1A2;KCNH5;ANKRD46;SEMA6D;ATP1B1;OLFM1;CACNB4;MSH4;AMPH;ZNF676;STEAP2;DGKI;GRIA2;SLC41A2;GPR88;SLC1A1;SLC1A3;ILDR2;GLYAT;LRRC55;CAMSAP2;KCNV1;ARHGAP20;CDH20;FUT9;TCP10L2;MORC1;RAB6B;LONRF2;GRIA3;ZNF540;KL;ANKRD29;CCDC13;PTPRD;LRCH2;FGF14;HOOK1;CNTN1;THRB;MCTP1;HSPB8;PTPN20;ELAVL4;ELAVL3;GRM3;GRM5;CALB1;GRM7;LRRTM3;GRM8;GDAP1L1;LRRTM2;HYDIN;SPX;UPP2;TRPC5;DCC;RTN4RL1;CDH12;SOWAHA;SSX2IP;HLF;DNAH5;PALM2;CAMK2A;ATP1A2;PPM1E;JAKMIP2;SV2C;HECTD2;CNR1;ERBB4;SLITRK4;CACNG2;SNCA;NTRK2;MCOLN3;NEGR1;PCDH7;EYA4;KIAA1549L;CCK;MAPK10;PPP2R2B;NOVA1;SYT10;ASXL3;BRMS1L;PI15;FSIP1;MDGA2;KCNK2
DORSAL STRIATUM	0.0062430	0.0561878	CHIC1;FRMPD4;HHIP;NEXMIF;TRIL;ANKRD20A1;ANKRD20A2;RXFP1;PREX2;NCKAP5;SCN1A;ANKS1B;SOX5;EPHA5;UNC13A;PRKCB;CACNA2D1;SLC6A15;UNC5D;ANK3;FNDC9;GABRG1;BCAN;ADGRB3;RFX4;TLN2;ASTN1;KIAA1211L;NECAB1;PCDH10;TMTC1;PCDH19;SEC14L2;MAATS1;GRIN2A;MAP2;TSPAN7;KCNN3;PPARGC1A;SCN3A;GPM6B;DTNA;CADM2;ST8SIA3;SLC16A14;HRNR;PEX5L;ANKRD20A4;STRN;PCSK2;GABRB2;RYR2;NCAN;BHLHE41;SLC7A14;GRIK1;GRIK2;SLC6A1;JPH1;TMEM47;PCLO;TRIM2;DLGAP1;ZNF365;APOBEC4;CYP7B1;SYN2;ANO5;CDYL2;TMEFF1;MMP16;PLSCR4;KCNMA1;RARB;RGS7BP;LRRC3B;PLCB1;PDZRN4;AMER2;FAXC;COL11A1;PLPPR4;PATE1;TMEM74;MIPOL1;PURA;FAM184A;MGAT3;CTNNA3;SH3BGR;P2RY12;RFTN2;GABRA1;MOG;KCNB1;GABRA5;GABRA4;DAB2IP;LSAMP;FMO2;SORBS1;NMNAT2;TMEM56;CAMK4;VWC2;IL17D;ZNF354B;CEP126;FAM13C;GALNT18;KLHL32;ZBTB20;FGF2;CELSR3;SLC8A1;AMOT;RGS4;ARHGAP44;GRIP1;RGS5;SACS;PPFIA2;GUCY1A2;TMEM178B;SEMA6D;CACNB1;OLFM1;CACNB4;MSH4;AMPH;ZNF676;DGKI;GRIA2;GPR88;SLC1A1;SLC1A3;KLHL11;LRRC55;CAMSAP2;ARHGAP20;CDH20;FUT9;CNTNAP3B;RAB6B;LONRF2;GRIA3;ZNF540;KL;NEB;PTPRD;ARHGAP32;LRCH2;PTPRB;FGF14;CNTN1;CNTNAP3;THRB;MCTP1;HSPB8;PTPN20;RERG;ELAVL3;GRM3;SEPT8;GRM5;CALB1;GRM7;LRRTM3;TRPS1;GRM8;ZIC1;GDAP1L1;LRRTM2;HYDIN;ITGB8;SPX;UPP2;TRPC5;DCC;CDH12;SOWAHA;HLF;DNAH5;PALM2;CAMK2A;PKD1L1;ATP1A2;LRP2;CXXC4;PPM1E;NWD1;SV2C;CNR1;ERBB4;REPS2;CACNG2;NTRK2;PCDH7;EYA4;KIAA1549L;CCK;ZIC5;ATP2B1;MAPK10;DBNDD2;APC;SYT11;PPP2R2B;SYT10;ASXL3;PI15;PDE7B;FSIP1;MDGA2;KCNK2

PLACENTA (BULK)	0.0214217	0.1779650	EIF4A2;ANTXR1;PREX2;EFR3A;HMCN1;DIP2B;FBXO9;FRS2;ACSL4;DICER1;ANK3;ISL1;CD2AP;UHMK1;CPED1;NHLRC2;BCORL1;GNE;MTMR1;PCDH10;MAOA;MYCBP2;GATA3;HSD11B1;ZNF703;PIP5K1B;CGA;JAG1;SIAH2;SIAH1;PPBP;VASN;ICK;MYO5B;ACER3;CALU;CPEB4;MTMR10;HEXB;TFCP2L1;NHSL1;MRC1;RSPO3;CNOT6L;SLC2A11;DUSP1;RRAS2;AP3B1;DUSP9;PRSS12;CDYL2;RHOB;SLC7A4;SLC7A8;RARB;ZNF117;CLDN16;PLCB1;VGLL1;ANGPTL1;YAP1;SLC20A1;SPESP1;SYNPO2;AKAP4;ADCY7;PURA;SCEL;NSD1;MGAT3;MBNL3;SORT1;USP9X;DAB2IP;SORBS1;MPP5;HIPK3;ESYT2;TRIP12;TACC2;ZNF354B;ZNF451;PRDM6;WWC1;LTN1;F13A1;WBP1L;AHR;HOXA13;IGF1R;AMOT;GRIP1;PTAR1;SLK;EP300;MAP3K8;SLC39A8;SLC39A1;KRT6B;GUCY1A2;SLC38A1;KIRREL1;SEMA6A;CD93;SEMA6D;LMO2;TTC7B;DYRK1A;TC2N;FAM91A1;FBXW2;HIC2;SMS;RAD54L2;IL6ST;UTRN;FBN2;NOTCH2;INO80D;MINDY2;SLC41A2;RHOBTB3;ILDR2;DTX4;PRPF8;RHOBTB1;CAMSAP2;HMBOX1;ATXN1;ATXN7;COBLL1;UBR5;RAB6B;SLC38A2;MYH10;RAP1GAP2;KL;ARHGEF35;SRD5A2;SEC16A;CRIM1;ESRRG;NEB;UBE2A;CALHM3;AZIN1;PTPRB;LIN28B;LRCH1;COL5A2;NF1;SLC26A7;TGFBI;BRWD1;EIF4G2;CPM;HSPB8;AMD1;FHL1;TCHH;CLEC14A;HAPLN1;ADAMTS5;CLEC1A;AP1G1;TMEM107;CEP170B;PHACTR2;MAN1A1;ITGB6;ADAMTS6;EGFL6;FGG;TGFBR1;TGFBR3;MMRN1;PALLD;ADGRF4;RASA1;PPP1R12B;MCTP2;NFAT5;COLEC10;CCDC186;BRAT1;ATP10D;SEMA3G;NDNF;LRP2;DLL4;HECTD1;CLMN;SAP30L;ASCC3;PDLIM5;SEPT10;FEM1B;AMFR;GALNT1;CCDC179;SPRY4;LAMB1;ACVR2B;PTPN14;DLK1;SGPP1;FAM167A;ASXL3;PI15
SPINAL CORD	0.0258226	0.1859230	CNTF;FRMPD4;NEXMIF;TRIL;COL12A1;ANKRD20A2;ANTXR1;SOGA1;PREX2;DPYSL3;HMCN1;NCKAP5;SCN1A;SOX5;EPHA5;UNC13A;CACNA2D1;SLC6A15;EBF1;SOX11;UNC5D;FNDC9;ISL1;GABRG1;BCAN;ADGRB3;RFX4;HOXB2;AGTR2;TLN2;FREM1;HOXB6;ASTN1;HOXB5;TSHZ3;PCDH10;TSHZ1;C7ORF57;PCDH19;EPB41L5;MAATS1;GRIN2A;MAP2;TSPAN7;KCNN3;PPARGC1A;SCN3A;HOXC8;GPM6B;ZBTB7C;JAG1;DTNA;CADM2;ST8SIA3;SLC16A14;HRNR;PEX5L;MEX3A;RYR2;NCAN;GRIK1;GRIK2;SIPA1L2;SLC6A1;ROBO1;ADAMTSL1;TMEM47;PCLO;TRIM2;DLGAP1;CYP7B1;DUSP9;SYN2;ANO5;PAX2;TMEFF1;MMP16;KCNMA1;RARB;RGS7BP;LRRC3B;PDZRN4;YAP1;AMER2;FAXC;COL11A1;PLPPR4;TMEM74;FAM184A;MGAT3;ZNF229;CTNNA3;RUNX1T1;P2RY12;GABRA5;DAB2IP;LSAMP;SORBS1;NMNAT2;VWC2;IL17D;ZNF354C;NEUROG2;FAM13C;GALNT18;KLHL32;CELSR3;AMOT;GRIP1;SALL1;MPZ;PPFIA2;GUCY1A2;KIRREL1;KCNH5;KIRREL2;SEMA6A;TMEM178B;SEMA6D;SHISA3;CACNB1;OLFM1;ADCYAP1;ZNF436;AMPH;DGKI;GRIA2;CALCB;KLHL14;GPR88;SLC1A1;SLC1A3;ILDR2;DTX4;LRRC55;CAMSAP2;SNN;ARHGAP20;NEUROD4;CDH20;FUT9;RAB6B;LONRF2;GRIA3;INHBB;ESRRG;PTPRD;ARHGAP32;LRCH2;FGF14;GPSM1;PTPN20;ELAVL4;ELAVL2;ELAVL3;GRM3;ADAMTS5;GRM5;ZIC2;GRM7;LRRTM3;GRM8;ZIC1;GDAP1L1;LRRTM2;CEP170B;HYDIN;ZNF521;TRPC5;EGFL6;DCC;NRG1;SETBP1;RTN4RL1;PALM2;CAMK2A;SEMA3G;IER5L;ATP1A2;NDNF;LRP2;CXXC4;PPM1E;DLL4;SV2C;FAM168A;CNR1;ERBB4;SLIT2;CACNG2;NTRK2;PCDH7;EYA4;KIAA1549L;CCK;ZIC5;SEMA4G;MAPK10;FAM167A;SYT11;PPP2R2B;NOVA1;SYT10;ASXL3;PI15;TRIM36;MDGA2;KCNK2
SPINAL CORD (BULK)	0.0258226	0.1859230	CNTF;FRMPD4;NEXMIF;TRIL;COL12A1;ANKRD20A2;ANTXR1;SOGA1;PREX2;DPYSL3;HMCN1;NCKAP5;SCN1A;SOX5;EPHA5;UNC13A;CACNA2D1;SLC6A15;EBF1;SOX11;UNC5D;FNDC9;ISL1;GABRG1;BCAN;ADGRB3;RFX4;HOXB2;AGTR2;TLN2;FREM1;HOXB6;ASTN1;HOXB5;TSHZ3;PCDH10;TSHZ1;C7ORF57;PCDH19;EPB41L5;MAATS1;GRIN2A;MAP2;TSPAN7;KCNN3;PPARGC1A;SCN3A;HOXC8;GPM6B;ZBTB7C;JAG1;DTNA;CADM2;ST8SIA3;SLC16A14;HRNR;PEX5L;MEX3A;RYR2;NCAN;GRIK1;GRIK2;SIPA1L2;SLC6A1;ROBO1;ADAMTSL1;TMEM47;PCLO;TRIM2;DLGAP1;CYP7B1;DUSP9;SYN2;ANO5;PAX2;TMEFF1;MMP16;KCNMA1;RARB;RGS7BP;LRRC3B;PDZRN4;YAP1;AMER2;FAXC;COL11A1;PLPPR4;TMEM74;FAM184A;MGAT3;ZNF229;CTNNA3;RUNX1T1;P2RY12;GABRA5;DAB2IP;LSAMP;SORBS1;NMNAT2;VWC2;IL17D;ZNF354C;NEUROG2;FAM13C;GALNT18;KLHL32;CELSR3;AMOT;GRIP1;SALL1;MPZ;PPFIA2;GUCY1A2;KIRREL1;KCNH5;KIRREL2;SEMA6A;TMEM178B;SEMA6D;SHISA3;CACNB1;OLFM1;ADCYAP1;ZNF436;AMPH;DGKI;GRIA2;CALCB;KLHL14;GPR88;SLC1A1;SLC1A3;ILDR2;DTX4;LRRC55;CAMSAP2;SNN;ARHGAP20;NEUROD4;CDH20;FUT9;RAB6B;LONRF2;GRIA3;INHBB;ESRRG;PTPRD;ARHGAP32;LRCH2;FGF14;GPSM1;PTPN20;ELAVL4;ELAVL2;ELAVL3;GRM3;ADAMTS5;GRM5;ZIC2;GRM7;LRRTM3;GRM8;ZIC1;GDAP1L1;LRRTM2;CEP170B;HYDIN;ZNF521;TRPC5;EGFL6;DCC;NRG1;SETBP1;RTN4RL1;PALM2;CAMK2A;SEMA3G;IER5L;ATP1A2;NDNF;LRP2;CXXC4;PPM1E;DLL4;SV2C;FAM168A;CNR1;ERBB4;SLIT2;CACNG2;NTRK2;PCDH7;EYA4;KIAA1549L;CCK;ZIC5;SEMA4G;MAPK10;FAM167A;SYT11;PPP2R2B;NOVA1;SYT10;ASXL3;PI15;TRIM36;MDGA2;KCNK2
